# Vortex Motions in the Solar Atmosphere: Definitions, Theory, Observations, and Modelling

**DOI:** 10.1007/s11214-022-00946-8

**Published:** 2023-01-06

**Authors:** K. Tziotziou, E. Scullion, S. Shelyag, O. Steiner, E. Khomenko, G. Tsiropoula, J. R. Canivete Cuissa, S. Wedemeyer, I. Kontogiannis, N. Yadav, I. N. Kitiashvili, S. J. Skirvin, I. Dakanalis, A. G. Kosovichev, V. Fedun

**Affiliations:** 1 Institute for Astronomy, Astrophysics, Space Applications and Remote Sensing, National Observatory of Athens, GR-15236 Penteli, Greece; 2 Department of Mathematics, Physics and Electrical Engineering, Northumbria University, Newcastle upon Tyne, NE1 8ST UK; 3 School of Information Technology, Deakin University, Geelong, Australia; 4 IRSOL–Istituto Ricerche Solari “Aldo e Cele Daccò”, Università della Svizzera italiana (USI), Via Patocchi 57, 6605 Locarno-Monti, Switzerland; 5 Leibniz-Institut für Sonnenphysik (KIS), Schöneckstrasse 6, 79104 Freiburg i.Br., Germany; 6 Instituto de Astrofísica de Canarias, 38205 La Laguna, Tenerife Spain; 7 Departamento de Astrofísica, Universidad de La Laguna, 38205 La Laguna, Tenerife Spain; 8 Center for Theoretical Astrophysics and Cosmology, Institute for Computational Science (ICS), University of Zurich, Winterthurerstrasse 190, 8057 Zürich, Switzerland; 9 Rosseland Center for Solar Physics, University of Oslo, P.O. Box 1029 Blindern, NO-0315 Oslo, Norway; 10 Institute of Theoretical Astrophysics, University of Oslo, P.O. Box 1029 Blindern, NO-0315 Oslo, Norway; 11 Leibniz-Institut für Astrophysik Potsdam (AIP), An der Sternwarte 16, 14482 Potsdam, Germany; 12 Max Planck Institute for Solar System Research, Justus-von-Liebig-Weg 3, 37077 Göttingen, Germany; 13 Present Address: Indian Institute of Science Education and Research, Thiruvananthapuram, Kerala 695551 India; 14 NASA Ames Research Center, Moffett Field, CA 94035 USA; 15 Plasma Dynamics Group, Department of Automatic Control and Systems Engineering, The University of Sheffield, Mappin Street, Sheffield, S1 3JD UK; 16 Present Address: Centre for Mathematical Plasma Astrophysics, Mathematics Department, KU Leuven, Celestijnenlaan 200B bus 2400, B-3001 Leuven, Belgium; 17 Department of Physics, New Jersey Institute of Technology, Newark, NJ 07102 USA

**Keywords:** Vortex flows, Sun, Solar atmosphere, Magnetohydrodynamic waves

## Abstract

Vortex flows, related to solar convective turbulent dynamics at granular scales and their interplay with magnetic fields within intergranular lanes, occur abundantly on the solar surface and in the atmosphere above. Their presence is revealed in high-resolution and high-cadence solar observations from the ground and from space and with state-of-the-art magnetoconvection simulations. Vortical flows exhibit complex characteristics and dynamics, excite a wide range of different waves, and couple different layers of the solar atmosphere, which facilitates the channeling and transfer of mass, momentum and energy from the solar surface up to the low corona. Here we provide a comprehensive review of documented research and new developments in theory, observations, and modelling of vortices over the past couple of decades after their observational discovery, including recent observations in $\text{H}\alpha $, innovative detection techniques, diverse hydrostatic modelling of waves and forefront magnetohydrodynamic simulations incorporating effects of a non-ideal plasma. It is the first systematic overview of solar vortex flows at granular scales, a field with a plethora of names for phenomena that exhibit similarities and differences and often interconnect and rely on the same physics. With the advent of the 4-m Daniel K. Inouye Solar Telescope and the forthcoming European Solar Telescope, the ongoing Solar Orbiter mission, and the development of cutting-edge simulations, this review timely addresses the state-of-the-art on vortex flows and outlines both theoretical and observational future research directions.

## Introduction

Vortices, naturally appearing in water and air flows, have always stimulated the imagination, craftsmanship, and art of many cultures and civilizations worldwide. Represented as spiral patterns and associated with different meanings and symbolisms (e.g., the Universe, harmony, the Sun, growth etc), they are one of the most enduring and dominating symbols in history. They appear as a motif in petroglyphs as far back as the Neolithic period (e.g. in a celtic tomb at Newgrange, Ireland) and hieroglyphs, in ornamentations such as ancient Greek and Roman jewelry, as dominating facial and body tattooing symbols in civilizations like the Maori people in New Zealand, or even as eye-catching features in modern art (e.g. “The Starry night” painting by Van Gogh). Vortex motions have been mathematically described even in ancient times by mathematicians such as Archimedes and Theodorus of Cyrene, while during the Renaissance, vortices have inspired artists such as Leonardo da Vinci in his studies of fluid flows, and philosophers and scientists such as René Descartes (aka Cartesius), who used them to model the dynamics of the solar system.

In physics, vortex motions, poetically described by Kuchemann (1965) as the *“sinews and muscles of fluid motions”*, are the subject of research in several disciplines involving fluid dynamics and turbulent motions such as atmospheric studies, aerodynamics, hydrodynamics, etc. They are important in understanding the complex evolution of physical phenomena on our planet (ocean water and atmospheric air circulation, hurricanes and cyclones, dust devils) but also in several of our technological endeavors that require good knowledge of fluid dynamics and their turbulent effects, most notably aviation. It is worth noting that, apart from Earth, vortices are prominent long-living features in the atmospheres of other planets such as the permanent Great Red Spot on Jupiter, the polar vortices of Venus, the great white spots of Saturn, the Martian dust devils, etc. There exist several formal definitions for vortices that were inspired by human visual perception of natural swirling motions. Lugt (1979) suggested that *“A vortex is the rotating motion of a multitude of material particles around a common centre”*. More helpful in our effort for detecting a three-dimensional vortex motion is Robinson (1990), who suggested the more elaborate but somewhat self-referential definition that *“A vortex exists when instantaneous streamlines mapped onto a plane normal to the vortex core exhibit a roughly circular or spiral pattern, when viewed from a reference frame moving with the center of the vortex core”*.

Despite the considerable progress and thriving literature concerning vortices in physical systems, in particular the terrestrial and planetary atmospheres, only little attention has been paid in the past to vortex motions in the solar atmosphere, although relevant theory to such motions exists now for several decades (e.g., Stenflo 1975; Schüssler 1984; Bünte et al. 1993). The recent boost in solar vortex studies at multiple scales was triggered by the advent of novel solar instrumentation, permitting high-resolution and high-cadence observations, combined with state-of-the-art simulations of equivalent spatial and temporal resolution. Both observations and simulations helped us in addressing the small scales involved in the generation of the majority of solar vortical motions. In the past decades, it has been shown that such motions are ubiquitous and present across a broad range of temporal and spatial scales on the solar surface^1^ and atmosphere, up to the corona, with the smaller ones mainly resulting from turbulent convection and its interaction with magnetic fields. They are thought to play an important role in the plasma dynamics at different solar atmospheric layers, and to provide a mechanism for channeling mass, momentum and energy from the photosphere higher up into the atmosphere.

Large vortex flows of supergranular scale exist in quiet Sun regions (Chian et al. 2020) and also in active regions—in the latter case resulting from the emergence of large magnetic flux ropes and often visually seen as rotation in sunspots (e.g., Brown et al. 2003; Gopasyuk and Kosovichev 2011). Both are related to large-scale subsurface or surface flows and motions, mainly associated with the physics of large-scale magnetic flux emergence in the case of active regions. These are not going to be discussed here. Instead, this review summarises the past efforts and new developments in theory, observations, and modelling of small-scale vortices related to convective turbulence at granular scales.

Such vortices, found in observations and simulations, are linked to various phenomena of the solar atmosphere and have therefore received diverse names in the literature. However, the connection, similarities, and differences between reported vortex-related phenomena are not always obvious despite the fact that some of them rely on the same underlying physics. This review attempts the first systematic and comprehensive overview of solar vortices in our effort to better understand the generation, governing physics and properties of vortices in the solar atmosphere.

The review is organised as follows: in Sect. 2, we provide and discuss different definitions of phenomena, commonly referred to as vortices in solar physics, that have appeared in the literature from analyses of swirling motions across multiple spatial scales and solar atmospheric layers. In Sect. 3, we discuss vorticity and related equations, including the MHD fundamentals of vorticity, while Sect. 4 provides an overview of methods for the detection of photospheric and chromospheric vortex motions. A variety of physical mechanisms of vortex formation in the solar atmosphere is presented in Sect. 5. Section 6 reviews observations and properties of vortex flows from below the solar photosphere (by helioseismology) through the solar atmospheric layers up to the corona. Numerical simulations of vortex motions, both magnetic and non-magnetic, are the subject of Sect. 7. Section 8 provides observational evidence of vortex-related oscillations and waves, an overview of simulations of waves within vortex motions, and spectropolarimetric diagnostics of simulated vortex flows. Section 9 addresses non-ideal effects in vortex flows. Finally, a summary and discussion is given in Sect. 10 and open issues and an outlook to future advances in solar vortex research are discussed in Sect. 11.

## Definitions and Nomenclature

Rotational or “vortex motions” in fluid dynamics have been historically first investigated by Helmholtz (1858) while a few years later Thomson (a.k.a. Lord Kelvin) (1869) in a paper on vortex motion analyzed cyclic irrotational motion in multiply-connected regions and provided elegant proofs for some of the theorems proposed by Helmholtz. By the end of the 19th century, a collective view of the current knowledge of vortex motions in hydrodynamics and the associated mathematics has been compiled by Lamb (1895, chapter VII). Since then, vortex motions and respective definitions of vortex motions in hydrodynamics have been the subject of several studies. Some of the most recent works concern a general classification of three-dimensional flow fields (e.g., Chong et al. 1990), vortex definition, identification and analysis of vortical structures (e.g., Jeong and Hussain 1995; Kida and Miura 1998; Haller 2005), and more recently, the identification of Lagrangian coherent structures (Haller 2015) related to the detection of vortices.

Despite the vast literature on vortex motions in hydrodynamic and magnetohydrodynamic contexts, it proved to be difficult to reach a strict definition of a vortex or a swirling motion, and even more difficult to relate this phenomenon to motions in solar and space plasmas. The very nature of vortex flows dictates that the phenomenon is scale-invariant, so that there should be no restriction regarding the apparent size of the vortex flow (Requerey et al. 2018). As a result, a wide variety of observed phenomena could be characterised by the same principles that underpin vortex physics. Yet, many of these phenomena are labelled differently depending on the instrument or the wavelength range used to observe them.

For practical purposes and in view of application to the solar atmosphere, we here generally define a vortex flow as follows: *“A vortex is the collective motion associated with the azimuthal component (*$\phi $*) of a vector field (e.g. true velocity or magnetic field) or its observational counterparts (such as trackable motions of features associated with radiation intensity or magnetic field) about a common centre or axis.”* In detection efforts, the flow region can be described as a vortex if this region i) has a high local vorticity, ii) is persistent in time, iii) is bounded, e.g. has a convex shape,^2^ and iv) exhibits circular motion. We note, however, that although this description pertains to the majority of discussed vortex flows in this review, there exist exceptions (such as, e.g., irrotational vortex flows) that do not satisfy, at least not fully, one or more of the above criteria.

Below, we provide a brief overview of the ever-growing, wide-ranging nomenclature that has been attributed to tornado-like flows, swirls, or vortices in a solar context in recent decades. Each of these items is accompanied by a contextual image with short description in Tables 1–4.

**Table 1:**
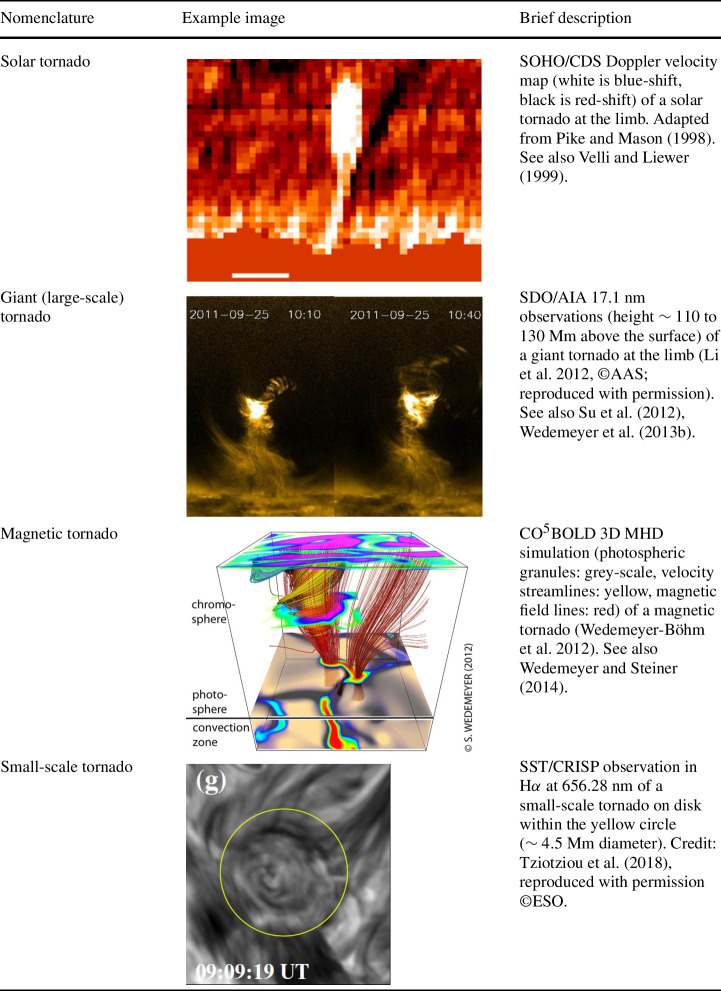
Classification of vortex motions as “tornadoes” in the solar literature (see main text for further details). We refer the reader to Sect. 10.1 for recommendations on used nomenclature

Since Pettit (1932), the term “tornado” has been repeatedly used to describe vortical phenomena on the Sun, particularly in relation to prominences. More recently, a number of quite different phenomena, both observed and simulated, have been classified in the solar literature as “tornado” (see Table 1 for context images), including:

- *Solar tornado*: A rotating (macrospicule-like) solar feature ($\sim 10''$ wide) with a shape that visually resembles a terrestrial tornado although the physics is different. First observed by Pike and Mason (1998) in the O v spectral emission line of the transition region, recorded with SOHO/CDS (Harrison et al. 1995). These features exhibit blue- and red-shifted emission on either side of a central axis extending above the solar limb and their rotation velocities increase with height.
- *Giant tornado*: Li et al. (2012) and Wedemeyer et al. (2013b) observed this type of feature in the SDO/AIA 171 Å channel and interpreted it as elongated, and apparently rotating structure. According to Wedemeyer et al. (2013b), they preferentially appear in groups and form the “legs” of prominences, thereby serving as plasma sources/sinks, while Li et al. (2012) link the observed vortex flows within the prominence body with the untwisting and expansion of its helical magnetic field structure. Most tornadoes have lengths in the range of $10''$ to $100''$ ($\sim 7~\text{Mm}$–75 Mm) and widths extending mostly from 1.5 Mm to 11.6 Mm. Doppler shifts around $20~\text{km}\,\text{s}^{-1}$ are measured that imply, for an observed radius of $\sim 5~\text{Mm}$, an angular velocity of $0.004~\text{rad}\,\text{s}^{-1}$ (Wedemeyer et al. 2013b). They may be closely associated with the previously described “Solar tornado” on a larger scale. Su et al. (2012) proposed they could be explained by a rotating magnetic structure (see later description for cyclones) driven by underlying photospheric vortex flows. We note, however, that there exist contradictory reports that come to the conclusion that giant tornadoes are non-rotating structures and do not represent true vortical mass flow (e.g., Schmieder et al. 2017b).
- *Magnetic tornado*: The theoretical concept of a coherent, rotating magnetic field structure (Wedemeyer-Böhm et al. 2012) that extends from the solar surface into the upper solar atmosphere (see also Wedemeyer and Steiner 2014). It is driven by swirling downflows (bathtub effect) in the surface layers of the convection zone, acting on magnetic field concentrations in the photosphere. In the chromosphere, magnetic tornadoes have diameters on the order of $4''$, with examples ranging from $2''$ to almost $8''$, and Doppler shifts corresponding to upward velocities of typically $4~\text{km}\,\text{s}^{-1}$ with even larger peak values.
- *Small-scale tornado*: To date, these have only been observed on-disk, most clearly in chromospheric spectral channels such as $\text{H}\alpha $, as a long duration persistent swirling flow (Tziotziou et al. 2018). The reported vortex has a cross-sectional diameter of $\sim 4.5~\text{Mm}$ with notable substructure of several smaller-scale chromospheric swirls (see next paragraph) within it and with considerable oscillatory behaviour, including rotational and swaying motions (Tziotziou et al. 2019). The observed vortex structure spans at heights up to the low corona but contrary to similarly sized magnetic tornadoes, does not seem to be magnetically driven as no related photospheric magnetic bright points have been observed.

A number of quite similar phenomena that are both observed and simulated have been classified as “swirl”. Generally, a swirl can be defined as *“a spiral pattern of (almost) circular shape that gives the impression of rotation”*. There are a number of these events reported in the literature (see also Table 2 for context images), such as:

- *Chromospheric swirl*: A term first used by Wedemeyer-Böhm and Rouppe van der Voort (2009) for the on-disk appearance of rotating features, discovered in narrow-band images in the Ca ii 8542 Å spectral line of a quiet Sun region inside a coronal hole. These swirls feature dark and bright rotating patches, which can consist of arcs, spiral arms, rings or ring fragments. They exhibit Doppler-shifted outflows of typically $2\text{--}4~\text{km}\,\text{s}^{-1}$. The typical diameter of swirls was initially reported as 1.5 Mm but larger examples have been observed since (Wedemeyer-Böhm et al. 2012). Chromospheric swirls are closely associated with tight groups of photospheric bright points that move relatively to each other. They are most likely the same objects as small-scale swirls (next item below) and the observational chromospheric signature of magnetic tornadoes.
- *Small-scale swirl*: A term, found in Shetye et al. (2019) for swirls observed in the chromospheric spectral lines of Ca ii, 854.2 nm and $\text{H}\alpha $, 656.28 nm with typical diameters of 1.5–2 Mm and relatively short lifetimes, that is interchangeably used with the term Chromospheric swirl.
- *Magnetic swirl*: Theoretical concept derived from three-dimensional numerical simulations of an open magnetic flux tube, embedded within a magnetohydrostatic and gravitationally stratified solar atmosphere, as reported by Chmielewski et al. (2014) and Murawski et al. (2018). The magnetic field at the base of the flux tube, corresponding to the top of the photosphere, is driven and twisted thereby exciting Alfvén waves, which propagate into the solar corona (Chmielewski et al. 2014). The initial twist of the magnetic field propagates outwards in a swirl-like manner. Such magnetic swirls have also been identified in self-consistent, realistic, numerical simulations of the solar atmosphere by Battaglia et al. (2021). The concept of magnetic swirls is essentially identical to that of magnetic tornadoes.
- *Swirling downdraft—non magnetic bright point*: Theoretical concept of swirling downdrafts that form in the deep photosphere as a consequence of angular momentum conservation, reminiscent of the water swirl in the outlet of a bathtub (bathtub effect). The centrifugal force of this swirl forms a funnel shaped depression in the optical surface of the Sun, causing a locally enhanced emission, called non-magnetic bright point, similar to but weaker than in the case of a magnetic bright point. First described by Moll et al. (2011b), Calvo et al. (2016) give a statistical analysis and coin the term.

**Table 2:**
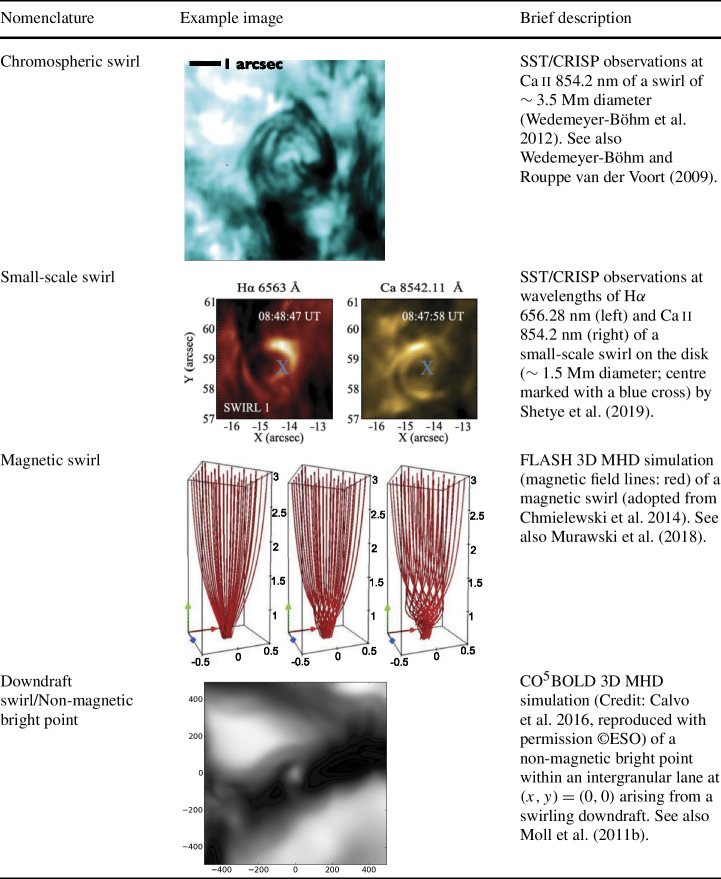
Classification of vortex motions as “swirls” in the solar literature (see main text for further details). We refer the reader to Sect. 10.1 for recommendations on used nomenclature

A number of simulated and observed phenomena have been classified with the “vortex” label or derivatives of this term (see Table 3 for context images). With the exception of the first item, the photospheric intensity vortices, they are all essentially theoretical concepts derived from simulations including:

- *Photospheric intensity vortices/swirls*: These vortices are attributed to the photospheric intergranular lanes and are not necessarily co-located with photospheric bright points or magnetic field concentrations. They were first observed in large numbers, via automated detection methods, by Giagkiozis et al. (2018) from SST/CRISP data. They suggest that at any time there are $1.48 \times 10^{6}$ events on the solar surface, implying that these vortices cover 2.8% of the total solar surface. They identified pairings of positive and negative vortices of similar characteristics in terms of lifetimes (many events lasting $\sim 8~\text{s}$), size scales (most events are around 560 km in diameter), and lateral motions (on average 0.42 km/s). Bifrost simulations (Liu et al. 2019b) theoretically confirmed this large number density and other observed properties of photospheric vortices. CO^5^BOLD simulations of Calvo et al. (2016) show a similarly large number density (0.0712 to 0.189 per $\text{Mm}^{2}$) of non-magnetic bright points, which they associate with photospheric vortices. Photospheric vortices have previously been associated with coronal heating (Zirker 1993) and kinematic modelling of vortices in the solar photosphere was carried out by Simon and Weiss (1997).
- *Vortex tube*: Muthsam et al. (2010) first found from a magnetic field-free simulation of the solar surface layers strong, rapidly rotating vortex tubes of small diameter ($\approx 100~\text{km}$) generated by the downdrafts and ascending into the photosphere, often in an arclike fashion. Moll et al. (2011b) confirmed this picture using the concept of swirling strength.
- *Horizontal vortex tube*: Vortex tubes that form in simulations of the solar surface layers at the boundaries of granules, approximately lying in a plane, parallel and beneath the visible solar surface (Steiner et al. 2010). Their observable signature are granular lanes consisting of a bright rim and a trailing dark edge that move from the granular boundary into the granule itself. In some cases, they transport magnetic fields into the granule interior (Fischer et al. 2020).
- *Magnetised vortex tubes*: Ubiquitous magnetized vortex tubes in simulations of the solar surface generated by the Sun’s turbulent convection in subsurface layers (Kitiashvili et al. 2013). The swirling vortex tubes (resembling tornadoes) penetrate into the solar atmosphere, capture and stretch background magnetic field, and push the surrounding material up, generating shocks.
- *Kinetic (*$K$*-)vortex*: $K$-vortices are coherent, twisted structures in the photospheric velocity field (see Fig. 17). They are detected based on Instantaneous Vorticity Deviation (IVD) or Lagrangian-Averaged Vorticity Deviation (LAVD) methods (Silva et al. 2020, 2021).
- *Magnetic (*$M$*-)vortex*: Coherent, twisted magnetic structure in photospheric magnetic fields, identified by Silva et al. (2021) (see Fig. 17). A formal definition for magnetic vortices is introduced based on the Integrated Averaged Current Deviation (IACD) method (Rempel et al. 2019). The IACD method together with an identification technique for kinetic vortices have been applied to realistic magneto-convection simulations with the MURaM code (Vögler et al. 2005). Magnetic vortices are distinguished from magnetic tornadoes by the magnetic field rather than the fluid exhibiting vortex behaviour in the solar atmosphere.

**Table 3:**
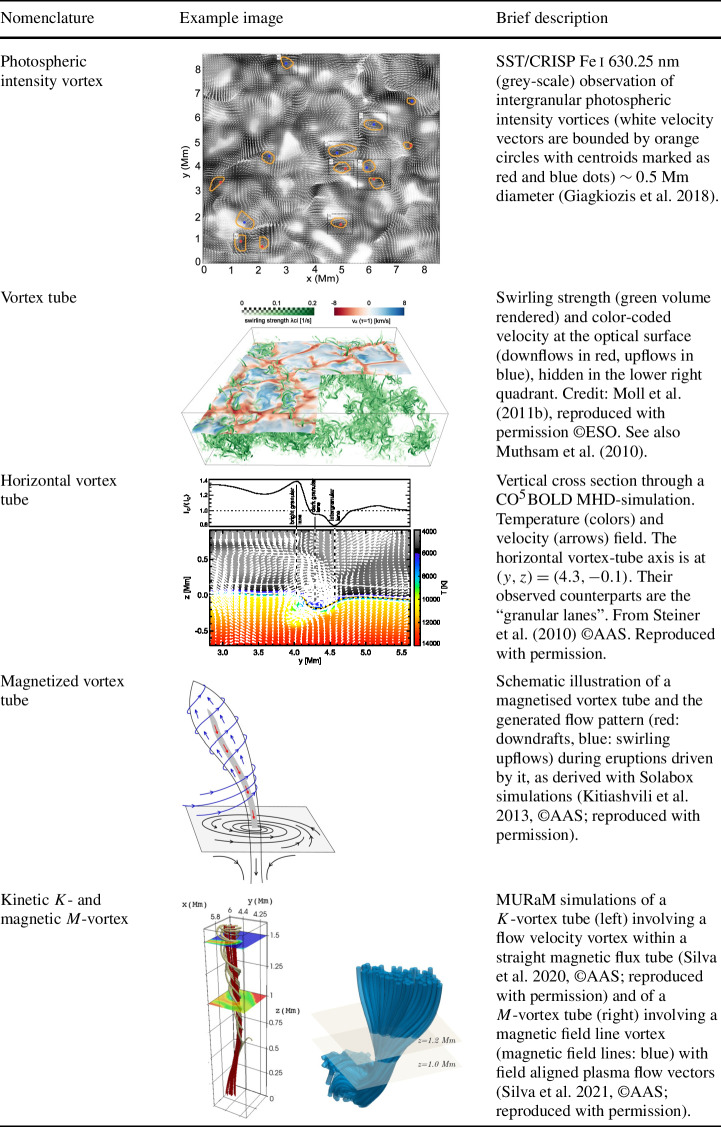
Classification of vortex motions as “vortices” in the solar literature (see main text for further details). We refer the reader to Sect. 10.1 for recommendations on used nomenclature

A number of observed phenomena labelled without using the terms tornado, swirl, or vortex, are also distinctive manifestations of vortical motions in the solar atmosphere (see Table 4 for context images). These are:

- *Whirlpools*: These were observed at disk centre at high spatial resolution with the SST, as a photospheric signature of convectively driven vortex flows by Bonet et al. (2008). They observed $0.9\times 10^{-2}$ vortices per $\text{Mm}^{2}$, with a lifetime of $\sim 5~\text{min}$, and with no preferred sense of rotation. The vortices appear to outline the supergranulation and the mesogranulation boundaries. They distinguish from photospheric intensity vortices (Table 3, first item) in that they are traced via the collective motion of photospheric bright points. They are characterized by a vortical flow field within which bright points appear as an advected substructure.
- *Rotating magnetic network fields (or EUV cyclones)*: EUV cyclones, observed in corresponding SDO/AIA and SDO/HMI channels, appear to be rooted in rotating network magnetic fields (RNFs) (Zhang and Liu 2011). They can last for more than ten hours and may be associated with EUV brightenings (microflares) and even with EUV waves. Zhang and Liu (2011) found 388 RNFs in an area of $800''\times 980''$ near disk center where no active region was present.

**Table 4:**
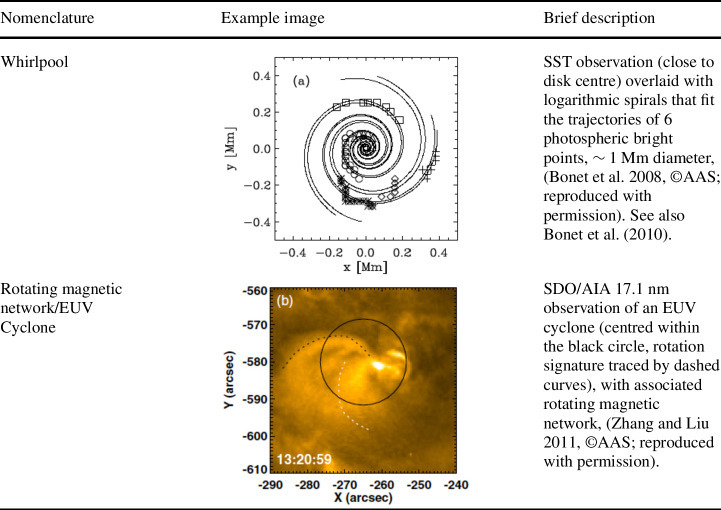
Other classifications of vortex motions in the solar literature (see main text for further details) not included in the previous tables. We refer the reader to Sect. 10.1 for recommendations on used nomenclature

Similarities and differences between the various phenomena listed in Tables 1 to 4, as well as recommendations on the nomenclature will be discussed in Sect. 10.1. Sections 5–7 give details on the observation and physics of these and additional vortical phenomena.

## Fundamental Equations

This section treats two characteristic quantities that are commonly used for the description of vortical mass motions, which are the vorticity and the swirling strength. It also presents different forms of the equations that governs their evolution.

### Vorticity and Helicity

Vorticity is a vector field that provides a measure of the rotationality of fluid motion. It requires knowledge of the velocity vector field, either taken directly from simulations or derived by application of correlation tracking techniques to observational images. Vorticity is defined as the curl of velocity $\boldsymbol{v}$,

1 $$ \boldsymbol{\omega} = \nabla \times \boldsymbol{v} \,. $$

The direction of the vorticity vector indicates the rotation axis according to the right-hand rule, while its magnitude is related to the strength of the angular velocity. For a rigid-body like rotation, $|\boldsymbol{\omega}| = 2 \varOmega $, where $\varOmega $ is the local angular velocity of the fluid elements. Hence, one can use vorticity as a criterion to detect vortices and describe their orientation in space.

We note that vorticity is different from helicity (Moreau 1961; Moffatt 1969), which is an invariant measure of the linkage and/or knottedness of vortex lines in the flow and is defined as

2 $$ H_{v}=\int _{V} \boldsymbol{v} \cdot (\nabla \times \boldsymbol{v}) {\mathrm{d}}V . $$

However, in solar physics, it is mostly magnetic helicity that is used, which is a conserved property associated with the distortion (twist, writhe) and linkage of the magnetic field lines compared to their potential-energy state (e.g., Berger 1984; Moffatt and Ricca 1992; Berger 1999; Brandenburg and Kerr 2001), defined as

3 $$ H_{M}=\int _{V} \boldsymbol{A} \cdot (\nabla \times \boldsymbol{A}) {\mathrm{d}}V, $$

where $\boldsymbol{A}$ is the magnetic vector potential. Nonetheless, vortical motions exerted at the footpoints of closed magnetic field lines can generate helicity by amplifying their twist and writhe. On the other hand helicity can generate vorticity during events, such as reconnection, that force a sudden reconfiguration of the magnetic field topology (mainly untwisting), towards its relaxation to lower and closer to potential magnetic free energy levels.

The vorticity $\boldsymbol{\omega}$ can be interpreted as a measure of the effective local angular velocity of the fluid elements. Let us take a small circle of radius $r$, then the average angular velocity of the fluid along the circumference of this circle is

4 $$ \frac{1}{2 \pi r}\oint \frac{\boldsymbol{v}}{r}\cdot \mathrm{d} \boldsymbol{l} \, , $$

where $\mathrm{d}\boldsymbol{l}$ is an infinitesimal element of the circle. Using Stokes’ theorem, one obtains

$$ \frac{1}{2 \pi r^{2}}\oint \boldsymbol{v}\cdot \mathrm{d}\boldsymbol{l} = \frac{1}{2 \pi r^{2}}\int _{S}\left ( \nabla \times \boldsymbol{v} \right )\cdot \boldsymbol{n}\, \mathrm{d}S = \frac{1}{2} \langle {\omega} \rangle \,, $$

where $\mathrm{d}S$ is an element of the surface enclosed by the circle and $\boldsymbol{n}$ is a unit vector normal to that surface. From this simple calculation we have proven that vorticity is twice the effective local angular velocity of the fluid (Batchelor 2000). If the angular velocity $\boldsymbol{v}/r$ is constant along the circle and radius, then the fluid rotation follows that of a rigid body. In that case, the vorticity is uniform over the surface and its direction is parallel to $\boldsymbol{n}$.

Flows for which $\boldsymbol{\omega} = 0$ are dubbed irrotational. A particular example is given by a vortical flow for which the velocity field is inversely proportional to the distance from the vortex core. Such a flow can be described, in cylindrical coordinates $(r, \phi , z)$, by

$$ \boldsymbol{v} = \left ( 0, \frac{k}{r}, 0\right ) \,, $$

where $k$ is a constant and the $r$ coordinate measures the distance from the vortex center. By taking the curl in cylindrical coordinates of this velocity field, one finds that the vorticity $\boldsymbol{\omega}$ is zero everywhere in the domain of the velocity vector. Therefore, the flow is irrotational and called a *free vortex*, nonetheless, by definition, it is globally rotating around the $r=0$ axis (Acheson 1990).

On the other hand, vorticity does not always imply global rotation of the fluid. A simple example is given by shear flows. Consider the velocity field of a Couette flow in Cartesian coordinates defined by

$$ \boldsymbol{v} = \left ( 0, \xi x, 0 \right )\, , $$

where $\xi $ is a constant that characterizes the shear. Then, the vorticity is

$$ \boldsymbol{\omega} = \left ( 0, 0, \xi \right )\, , $$

which is not zero even though the flow does not rotate. More details on the inadequacy of the vorticity on the detection of vortices can be found in Jeong and Hussain (1995).

### Vorticity Equations

To study vortices in the solar atmosphere, it is necessary and convenient to derive an evolution equation for the vorticity so that different source terms for the vorticity generation can be investigated. To obtain the vorticity equation, we rewrite the standard MHD momentum equation in the form

5 $$ \frac{\mathrm{d}\boldsymbol{v}}{\mathrm{d}t}=-\frac{1}{\rho}\nabla (p_{ \mathrm{g}}+p_{\mathrm{m}})+\frac{1}{\rho}(\boldsymbol{B}\cdot \nabla ) \boldsymbol{B}\,. $$

Here, $\boldsymbol{B}$ is the magnetic field, $\boldsymbol{v}$ is the plasma flow velocity, $\rho $ is the plasma mass density, $\frac{\mathrm{d}}{\mathrm{d}t}=\frac{\partial}{\partial t}+ \boldsymbol{v}\cdot \nabla $ is the total material derivative, and $p_{\mathrm{g}}$ and $p_{\mathrm{m}}$ are the thermal gas and magnetic pressure, correspondingly.

Expanding the material derivative on left hand side of Eq. (5) and using the vector identity $(\boldsymbol{u}\cdot \nabla )\boldsymbol{u}=\frac{1}{2}\nabla \boldsymbol{u}^{2} - \boldsymbol{u}\times (\nabla \times \boldsymbol{u})$, it can be re-written as

6 $$ \frac{\partial \boldsymbol{v}}{\partial t}+\frac{1}{2} \nabla \boldsymbol{v}^{2} - \boldsymbol{v}\times \boldsymbol{\omega}=- \frac{1}{\rho}\nabla (p_{\mathrm{g}}+p_{\mathrm{m}})+\frac{1}{\rho}( \boldsymbol{B}\cdot \nabla )\boldsymbol{B}\,, $$

where $\boldsymbol{\omega}$ is the vorticity of the fluid. Next, taking the curl of the above equation and invoking the vector identity $\nabla \times \nabla \psi =0$, where $\psi $ is a scalar quantity, we obtain the evolution equation for the vorticity,

7 $$\begin{aligned} \frac{\partial \boldsymbol{\omega}}{\partial t} =& - \left (\boldsymbol{v} \cdot \nabla \right )\boldsymbol{\omega} - \boldsymbol{\omega}\left ( \nabla \cdot \boldsymbol{v}\right ) +\left ( \boldsymbol{\omega}\cdot \nabla \right )\boldsymbol{v} -\nabla \frac{1}{\rho} \times \nabla p_{ \mathrm{g}} \\ &{}-\nabla \frac{1}{\rho} \times \left [ \nabla p_{\mathrm{m}} - \left ( \boldsymbol{B}\cdot \nabla \right )\boldsymbol{B}\right ] +\frac{1}{\rho} \nabla \times \left [ \left (\boldsymbol{B}\cdot \nabla \right ) \boldsymbol{B} \right ]. \end{aligned}$$

In the above equation, the various terms on the right hand side of the equation correspond to the physical mechanisms of the generation or destruction of vorticity. The first line corresponds to pure hydrodynamics. Here, the source terms are from left to right the advection of vorticity by the velocity field $\boldsymbol{v}$, vortex stretching (for compressible fluids), tilting due to flow gradients, and baroclinic vorticity generation.

Likewise, we have separated the terms which include the magnetic field $\boldsymbol{B}$ into two expressions in the second line of Eq. (7). The first expression is similar in its form to the baroclinic term of the pure hydrodynamics part. The bracket in this expression, however, contains the difference between the magnetic pressure force $\boldsymbol{\nabla}p_{\mathrm{m}} = \boldsymbol{B}(\boldsymbol{\nabla} \cdot \boldsymbol{B})$ and the restoring force by magnetic tension $(\boldsymbol{B}\cdot \boldsymbol{\nabla})\boldsymbol{B}$. Therefore, this term is equal to zero if the magnetic field is force-free and can be neglected in those regions of the solar atmosphere where the magnetic field is expected to be so. The last term in Eq. (7) contains the curl of the restoring force of magnetic tension. For more details on the different terms and their relative importance, we refer readers to previous works in this direction (Stein and Nordlund 1998; Shelyag et al. 2011b; Canivete Cuissa and Steiner 2020).

Another form of the vorticity equation can be obtained by expanding the material derivative of the vorticity and using Faraday’s law to obtain

8 $$ \frac{\partial \boldsymbol{\omega}}{\partial t}= \nabla \times ( \boldsymbol{v}\times \boldsymbol{\omega})- \frac{\nabla p_{\mathrm{g}} \times \nabla \rho}{\rho ^{2}}+\nabla \times \bigg[\frac{\boldsymbol{j}\times \boldsymbol{B}}{\rho}\bigg]\,. $$

This form of the evolution equation of vorticity represents an analogy to the evolution equation for the magnetic field in MHD. In a uniform non-magnetic fluid this equation reduces to

9 $$ \frac{\partial{\boldsymbol{\omega}}}{\partial t}=\nabla \times ( \boldsymbol{v}\times \boldsymbol{\omega})\,. $$

Thus, in a uniform, non-magnetic fluid, vorticity evolves in a similar fashion as the magnetic field evolves in ideal MHD, that is according to the ideal induction equation

10 $$ \frac{\partial \boldsymbol{B}}{\partial t}=\nabla \times (\boldsymbol{v} \times \boldsymbol{B})\,. $$

Including a viscous dissipation term, the vorticity equation for a uniform, non-magnetic fluid may be re-written as

11 $$ \frac{\partial \boldsymbol{\omega}}{\partial t}=\nabla \times ( \boldsymbol{v}\times \boldsymbol{\omega})+\nu \nabla ^{2} \boldsymbol{\omega}\,. $$

Here, $\nu $ represents the uniform kinematic viscosity of the fluid. The ratio of the length scale $L$ corresponding to the first term (vorticity freezing) to the second term (vorticity diffusion) on the right hand side of Eq. (11) defines the fluid Reynolds number

12 $$ \mathrm{Re} = \frac{|< \boldsymbol{v}>| L}{\nu}\,. $$

Similarly, including resistive dissipation, the induction equation can be re-written as

13 $$ \frac{\partial \boldsymbol{B}}{\partial t}=\nabla \times (\boldsymbol{v} \times \boldsymbol{B})+\eta \nabla ^{2} \boldsymbol{B}\,. $$

Here, $\eta $ represents the uniform magnetic diffusivity of the medium. Although Eqs. (9), (11) and Eqs. (10), (13) look strikingly similar, the evolution of the vorticity is nonlinear because $\boldsymbol{\omega} =\nabla \times \boldsymbol{v}$, while the evolution of the magnetic field is linear. In analogy with the fluid Reynolds number, in a magnetized fluid, the magnetic Reynolds number, $\mathrm{Re}_{\mathrm{M}}$, is defined as the ratio of the magnetic flux freezing term and the magnetic flux diffusion term in the induction equation, given as

14 $$ \mathrm{Re}_{\mathrm{M}} = \frac{|< \boldsymbol{v}>|L}{\eta}. $$

When $\mathrm{Re}_{\mathrm{M}}\gg 1$, magnetic field lines are effectively frozen into the fluid, while when $\mathrm{Re}_{\mathrm{M}}\ll 1$, resistive dissipation dominates. In solar plasmas, $\mathrm{Re}_{\mathrm{M}}$ is usually much larger than 1 and therefore, resistivity can generally be ignored. However, there can be small-scale discontinuities, where $\mathrm{Re}_{\mathrm{M}}$ can be locally small enough so that magnetic reconnection can take place. Also in parts of the photosphere, the ionization degree can be very low and ambipolar diffusion can come into play (see Sect. 9).

### Swirling Strength

Another criterion that has been widely used in solar physical applications (e.g. Moll et al. 2011b; Kato and Wedemeyer 2017; Battaglia et al. 2021) is the swirling strength $\lambda $, proposed by Zhou et al. (1999). The main advantage of this quantity with respect to vorticity is that it is not affected by the presence of shear flows.

The swirling strength can be seen as a generalization of the vorticity and it is defined through the eigenanalysis of the velocity gradient tensor $\mathcal{U}_{ij} \equiv \partial _{j} v_{i}$,

15 $$ \boldsymbol{\mathcal{U}} = \begin{bmatrix} \partial _{x} v_{x} & \partial _{y} v_{x} & \partial _{z} v_{x} \\ \partial _{x} v_{y} & \partial _{y} v_{y} & \partial _{z} v_{y} \\ \partial _{x} v_{z} & \partial _{y} v_{z} & \partial _{z} v_{z} \end{bmatrix} \,, $$

which physically encodes local variations of the velocity field.

In a vortex region, the velocity gradient tensor has a complex conjugated pair of eigenvalues (Chong et al. 1990) and it can be diagonalized in the following form

$$\begin{aligned} \boldsymbol{\mathcal{U}} =& \underbrace{(\boldsymbol{u}_{\mathrm{r}}, \boldsymbol{u}_{+}, \boldsymbol{u}_{-})}_{\mathcal{P}} \begin{bmatrix} \lambda _{\mathrm{r}} & 0 & 0 \\ 0 & ~\,\lambda _{+} & 0 \\ 0 & 0 & ~\lambda _{-} \end{bmatrix} \underbrace{ (\boldsymbol{u}_{\mathrm{r}}, \boldsymbol{u}_{+}, \boldsymbol{u}_{-})^{-1}}_{ \mathcal{P}^{-1}}\,, \end{aligned}$$

where $\lambda _{r}$, $\lambda _{+}$, and $\lambda _{-}$ are the eigenvalues of $\mathcal{U}$, while $\boldsymbol{u}_{\mathrm{r}}$, $\boldsymbol{u}_{\mathrm{+}}$, and $\boldsymbol{u}_{\mathrm{-}}$ are the corresponding eigenvectors that form the change-of-basis matrix $\mathcal{P}$ and its inverse $\mathcal{P}^{-1}$.

The two complex eigenvalues, $\lambda _{+}$ and $\lambda _{-}$, can also be defined as $\lambda _{\pm} = \lambda _{\mathrm{cr}} \pm {\mathrm{i}} \lambda _{ \mathrm{ci}}$, where $\lambda _{\mathrm{cr}}$ and $\lambda _{\mathrm{ci}}$ are real parameters. Similarly, one can write the complex eigenvectors as $\boldsymbol{u}_{\pm} = \tfrac{1}{\sqrt{2}}\left (\boldsymbol{u}_{ \mathrm{cr}} + {\mathrm{i}}\boldsymbol{u}_{\mathrm{ci}}\right )$, where $\boldsymbol{u}_{\mathrm{cr}}$ and $\boldsymbol{u}_{\mathrm{ci}}$ are real vectors. Then, one can express the local streamlines in a curvilinear coordinate system $(y_{1}, y_{2}, y_{3})$ spanned by the real vectors $(\boldsymbol{u}_{\mathrm{r}}, \boldsymbol{u}_{\mathrm{cr}}, \boldsymbol{u}_{ \mathrm{ci}})$ as

16 $$\begin{aligned} \begin{aligned} y_{1}(t) &= y_{1}(0) \exp{ (\lambda _{\mathrm{r}} t) }\,, \\ y_{2}(t) &= \exp{ (\lambda _{\mathrm{cr}} t) }\left [ y_{2}(0)\cos{ ( \lambda _{\mathrm{ci}} t) } + y_{3}(0)\sin{ (\lambda _{\mathrm{ci}} t) }\right ] \,, \\ y_{3}(t) &= \exp{ (\lambda _{\mathrm{cr}} t) }\left [ y_{3}(0)\cos{ ( \lambda _{\mathrm{ci}} t) } - y_{2}(0)\sin{ (\lambda _{\mathrm{ci}} t) }\right ] \,, \end{aligned} \end{aligned}$$

where $y_{1}(0)$, $y_{2}(0)$, and $y_{3}(0)$ are determined by the initial conditions.

The strength of the local swirling motion is characterized by the imaginary part of the complex eigenvalues, $\lambda _{\mathrm{ci}}$, which is related to the period of orbit $T$ of a fluid particle in a purely rotational vortex by $T = 2\pi /\lambda _{\mathrm{ci}}$ (Zhou et al. 1999). Standalone, this quantity defines yet another criterion: the $\lambda _{\mathrm{ci}}$-criterion, which is usually used together with a minimum threshold value, $\lambda _{\mathrm{ci}} > \epsilon $, in order to discard weak vortex detections.

However, we can extract more information from the eigen-analysis of $\mathcal{U}$: the real eigenvector $\boldsymbol{u}_{\mathrm{r}}$ identifies the vortex axis, that is the direction around which the flow is rotating or spiraling. Therefore, one can define the swirling (strength) vector $\boldsymbol{\lambda} = \lambda \boldsymbol{u}_{\mathrm{r}}$, where $\lambda = \lambda _{\mathrm{ci}}$, which provides the strength, the direction, and the orientation of the vortex. However, this definition can not be directly applied since the orientation of $\boldsymbol{u}_{\mathrm{r}}$ is mathematically arbitrary. A necessary condition to ensure the correct orientation of the vortex axis (according to the right-hand rule) is to choose $\boldsymbol{u}_{\mathrm{r}}$ such that $\mathrm{Im}[\det (P)] > 0$, where $P = (\boldsymbol{u}_{\mathrm{r}}, \boldsymbol{u}_{+}, \boldsymbol{u}_{-})$ is the change-of-basis matrix (Canivete Cuissa and Steiner 2020). Finally, notice that Canivete Cuissa and Steiner (2020) define the swirling strength intensity as $\lambda = 2 \lambda _{\mathrm{ci}}$. The extra 2 factor ensures that, for a rigid-body rotation, both swirling strength and vorticity return the same value.

### Swirling Strength Equation

An evolution equation for the swirling strength has recently been derived by Canivete Cuissa and Steiner (2020). This equation is more reliable than the vorticity equation, Eq. (7), because the swirling strength is not affected by shears; however, it is also more involved analytically since it requires the computation of the eigenvalues and eigenvectors of the velocity gradient tensor $\mathcal{U}_{ij}$. Moreover, both criteria fail to detect vortical flows of very specific structure such as irrotational vortices with $\boldsymbol{v} = (0, v_{\phi}, 0)$ in cylindrical coordinates where $v_{\phi} \propto 1/r$. Nonetheless, irrotational vortices are difficult to realize in nature.

The swirling equation can be derived by taking the tensor product of the gradient operator $\boldsymbol{\nabla}$ with the ideal MHD momentum equation and by using the diagonalization properties of $\mathcal{U}$:

17 ddtλ={P−1[∇⊗(1ρ∇pg)]P}−2λλcrTλ1−2Im{P−1[∇⊗(1ρ∇pg)]P}22Tλ2−2Im{P−1[∇⊗(1ρ∇pm)−(∇1ρ)⊗(B⋅∇)B]P}22Tλ3+2Im{P−1[1ρ∇⊗((B⋅∇)B)]P}22Tλ4{P−1[∇⊗(1ρ∇pg)]P}−2Im{P−1[∇⊗(∇Φ)]P}22,Tλ5

where $\lambda = 2 \lambda _{\mathrm{ci}}$, $\mathcal{P}$ and $\mathcal{P}^{-1}$ are respectively the eigenvector matrix of $\mathcal{U}$ and its inverse, and $\lambda _{\mathrm{cr}}$ is the real component of the complex conjugate eigenvalues. For more details on the derivation of Eq. (17), the reader can refer to Canivete Cuissa and Steiner (2020).

The authors also separated the source terms according to their physical interpretation. Notice that the different terms being multiplied by $\mathcal{P}^{-1}$ and $\mathcal{P}$ between curly brackets are matrices, and that the subscript 22 refers to the matricial component of the resulting multiplication. The first one, $T^{1}_{\lambda}$, can be seen as a vortex stretching term, while $T^{2}_{\lambda}$ represents the generation of swirling strength by hydrodynamical baroclinicity. Similarly to Eq. (7), the magnetic contributions have been separated into two terms: the first one, $T^{3}_{\lambda}$, resembles in form the baroclinic term, and it is therefore dubbed as magnetic baroclinic term, while the second, $T^{4}_{\lambda}$, includes the magnetic tension effects. Finally, the last term of Eq. (17) has no analogue in the vorticity equation and it is associated with the potential of conservative forces. One can interpret the swirling equation as the vorticity equation, Eq. (7), expressed in the reference frame proper to the vortex: in fact, one can rewrite Eq. (17) in a more compact way,

$$ \frac{\mathrm{d}}{{\mathrm{d}}t}\lambda = - 2 \lambda _{\mathrm{cr}} \lambda + 2 {\mathrm{Im}}\left ( \mathcal{P}^{-1}\mathcal{M} \mathcal{P} \right )_{22}\,, $$

where ℳ is the transpose of the matrix one obtains by applying the gradient to the source terms of the ideal MHD momentum equation. Then one can see that the matrices $\mathcal{P}^{-1}$ and $\mathcal{P}$ are essentially performing a change of basis on the source terms from the standard Euclidean basis to the one defined by the eigenvectors of the velocity gradient tensor $\mathcal{U}$. In this new basis, the properties of the vortex are described by the eigenvalues while other characteristics of the flow, which do not contribute to the vortex, e.g. shears, are implicitly left out.

Using numerical modelling, Shelyag et al. (2011b) demonstrated that the magnetic baroclinic term does not make a significant contribution to the vorticity generation in the lower solar atmosphere and the solar interior. In regions where the dynamics are dominated by the magnetic field however, the magnetic tension term is the main source of vorticity. Canivete Cuissa and Steiner (2020) compared the terms of the vorticity and swirling equations of numerical simulations of the solar atmosphere. They found that, in the convection zone, the production of vertical vorticity is primarily due to the tilting term of Eq. (7), while the formation of vertical swirling strength is dominated by the hydrodynamic and magnetic baroclinic terms of Eq. (17), even though these two terms have often opposite signs, therefore canceling out each other. Moreover, they demonstrated that, in the photosphere and low-chromosphere, both magnetic baroclinic and magnetic tension terms are important for the production of swirling strength. Finally, Battaglia et al. (2021) used the swirling equation to prove that uprising pulses of vertical swirling strength, which form at photospheric levels and manifest as chromospheric swirls, are driven by magnetic tension forces, thus demonstrating their Alfvénic nature.

These examples demonstrate that it is of fundamental interest to reliably detect vortices and to characterize and study their dynamics through analysis of their evolution equations.

## Detection Methods

The proper detection of vortex flows is important for any subsequent statistical or physical analysis. Following the different definitions of vortex flows, both in hydrodynamic terms and solar physics terms (see Sect. 2), various detection methods have been proposed in the literature and applied to both observational and computational datasets. Below, we describe in detail the quantities and algorithms that have been extensively used in solar physics for the detection of vortices.

For completeness, we note that there exist a number of other detection methods that have not acquired much attention from the solar community, such as the Helicity method by Levy et al. (1990), the Predictor-Corrector method by Banks and Singer (1995), the Parallel Vectors method by Roth and Peikert (1998), the Combinatorial method by Jiang et al. (2002), and the Swirl Parameter method by Berdahl and Thompson (1993).

The majority of the available detection methods requires knowledge of the horizontal velocity field within the analysed field-of-view (FOV). Its derivation relies on Local Correlation Tracking techniques (LCT; November and Simon 1988), Coherent Structure Tracking (CST; Rieutord et al. 2007), Fourier Local Correlation Tracking (FLCT; Fisher and Welsch 2008), or on the use of more advance techniques involving convolutional neural networks (e.g., DeepVel, Asensio Ramos et al. 2017). In most numerical simulations, all three components of the velocity field are usually obtained at very high resolution and accuracy; therefore LCT techniques are redundant. Such techniques are usually applied to relevant 2D intensity maps or magnetograms that are obtained either from observations or numerical simulations with no provision of horizontal velocities. However, we note that in observations, when intensity maps are considered, LCT methods are not suitable for the detection of very small photospheric vortices that do not have associated rotating bright points. Moreover, they do not provide reliable detection of vortex flows seen in chromospheric spectral lines, such as $\text{H}\alpha $ and Ca ii 8542 Å, as the chromosphere appears highly dynamic. Hence, a different approach is necessary in these situations (see Sect. 4.8).

We note that proper visualization of vortex flows should take into account that vortex flows are an integral part of complex three-dimensional flow patterns that change significantly on short timescales. Plotting streamlines of a 3D flow field of a single 3D simulation snapshot implicitly assumes that the flow field is stationary, while in fact it changes in time. The resulting streamlines give a first impression of the vortex flow but must not be interpreted as trajectories of (test) particles. Realistic particle trajectories require that the full time-dependent 3D flow field is taken into account by tracking test particles in time. As shown by Shelyag et al. (2013) and Wedemeyer and Steiner (2014), the resulting trajectories may differ from the instantaneous streamlines.

In the following subsections, we introduce various criteria that have been used for the detection of vortices.

### Vorticity

Vorticity, defined as $\boldsymbol{\omega} = \nabla \times \boldsymbol{v}$ (see Sect. 3.1 for further details), is a natural quantity for vortex characterization. However, it is now well known that it is a less suitable criterion for their identification (see, e.g., Jeong and Hussain 1995), because vorticity cannot distinguish between actual swirling (vortical) motions and shear flows. For example, the vorticity magnitude is maximal at the wall boundaries in a Poiseuille flow because of the strength of the shears, even though the fluid does not rotate. Similarly, a non-zero constant vorticity characterizes a Couette flow even though the streamlines are all straight and parallel.

### Maximum Vorticity Method

The maximum vorticity method, introduced by Strawn et al. (1999), is motivated by the fact that most vortex identification techniques are not able to identify overlapping vortex cores with the same sense of rotation when the overall velocity field outlines one single rotational centre. In aerodynamics applications, each vortex center can have a great impact to devices like air foils because of its high velocity gradient relatively to the local flow. To address this problem, Strawn et al. (1999) define a vortex center as a local maximum of vorticity $\lvert \boldsymbol{\omega} \rvert $ in the plane perpendicular to the vorticity vector $\boldsymbol{\omega}$. In this way, shear flows should not affect the detection: although they generate high vorticity values, they should not produce local maxima.

As a first step, one computes the vorticity vector $\boldsymbol{\omega}$ on a uniform hexahedral grid. Then, one must analyse $3 \times 3$ blocks of grid cells, and check whether a local maximum of vorticity is present in one of the 4 vertices of the central face. If this is the case, the central face is selected for the next step, where the exact location of the maximum is computed. To do that, one describes the gradients of vorticity $\nabla{\lvert \boldsymbol{\omega} \rvert}$ using bilinear interpolation functions on the $2{\mathrm{D}}$ plane of the candidate face. The coefficients of the bilinear equations can be determined by the known values of $\lvert \boldsymbol{\omega} \rvert $ at the vertices of the face. Solving for $\nabla{\lvert \boldsymbol{\omega} \rvert} = 0$ results in the $2{\mathrm{D}}$ coordinates of the local maximum: if it lies within the face, then that point corresponds to a vortex core. In addition, Strawn et al. (1999) used threshold values to remove detections that fit the requirements only marginally. An algorithm employing the maximum vorticity method is outlined by Jiang et al. (2005).

The maximum vorticity method can be used to identify vortex centers in regions where multiple rotational flows are overlapping and the overall picture seems to describe a single vortical motion. Moreover, Strawn et al. (1999) also suggested that this method could also be employed to locally modify the grid resolution in adaptive mesh refinement codes in order to improve the characterization of vortices in simulations (see e.g., Kasmai et al. 2011).

### $\varGamma $-Functions Method

The $\varGamma $-functions method is a widely used method for identifying vortex centers and boundaries. The method was proposed by Graftieaux et al. (2001) to identify vortices in turbulent hydrodynamic flows but applies also to solar atmospheric plasma flows once the horizontal velocity field is obtained, for example, with LCT techniques (Welsch and Fisher 2008). The main principles of the $\varGamma $-functions method are as follows. Two functions $\varGamma _{1}$ and $\varGamma _{2}$ are defined that are used to identify vortex centers and boundaries, respectively. The function $\varGamma _{1}$ is defined according to:

18 $$ \varGamma _{1}(\boldsymbol{x}_{p}) = \frac{1}{|S|} \sum _{S} \frac{\left ((\boldsymbol{x}_{m} - \boldsymbol{x}_{p}) \times \boldsymbol{v}_{m}\right ) \cdot \mathbf{1}_{z}}{||\boldsymbol{x}_{m} - \boldsymbol{x}_{p}||_{2} \cdot ||\boldsymbol{v}_{m}||_{2}}. $$

Here, $S = \{ \boldsymbol{x}_{m} \, : \, ||\boldsymbol{x}_{m} - \boldsymbol{x}_{p}||_{2} \leq R \}$ is a disk of radius $R$ about the point $\boldsymbol{x}_{p}$, $||\cdot ||_{2}$ represents the Euclidean norm, $\mathbf{1}_{z}$ is a unit vector normal to the plane of $S$, and $|S|$ is the cardinality of $S$. $\varGamma _{1}$ defines a scalar field and achieves its maximum value of unity when $\boldsymbol{x}_{p}$ is at the center of an axisymmetric vortex.

The vortex boundary identification (see, e.g., Giagkiozis et al. 2018) uses the discrete version of $\varGamma _{2}$, defined as follows,

19 $$ \varGamma _{2}(\boldsymbol{x}_{p}) = \frac{1}{N} \sum _{S} \frac{\left ((\boldsymbol{x}_{m} - \boldsymbol{x}_{p}) \times (\boldsymbol{v}_{m} - \bar{\boldsymbol{v}}_{p})\right ) \cdot \mathbf{1}_{z}}{||\boldsymbol{x}_{m} - \boldsymbol{x}_{p}||_{2} \cdot ||\boldsymbol{v}_{m} - \bar{\boldsymbol{v}}_{p}||_{2}}, $$

where $\bar{\boldsymbol{v}}_{p}$ is the mean velocity in the neighborhood of the point $\boldsymbol{x}_{p}$. It has been shown by Graftieaux et al. (2001) that in the inner core of a vortex, the magnitude of $\varGamma _{2}$ is larger than $2/\pi $. Flows with values of $\varGamma _{2}$ lower than $2/\pi $ are dominated by strain, and pure shear is when $\varGamma _{2}$ is equal to $2/\pi $. These values were obtained by assuming an incompressible flow and $S\rightarrow 0$.

**Fig. 1:**
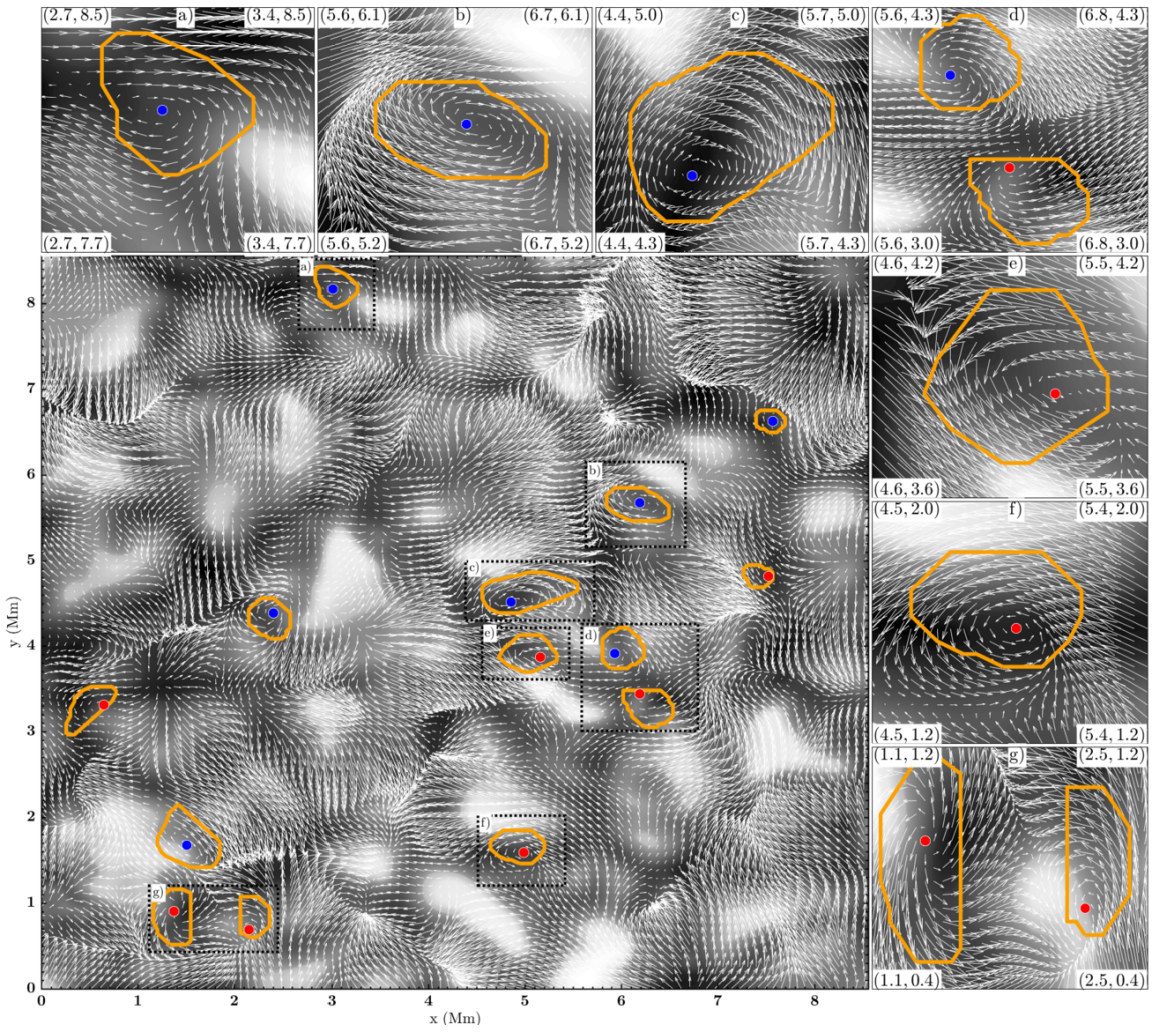
The LCT-estimated velocity field derived from the continuum intensity near Fe i 6302.5 Å (shown in grayscale). Red/blue circles denote the identified vortex centers corresponding to counter-clockwise/clockwise vortices and orange border lines show the respective vortex boundaries. These vortex centers correspond to the maximum magnitude of the $\varGamma _{1}$ function which achieves a value of unity at the center of an axisymmetric vortex (Graftieaux et al. 2001). By taking into account that perfect axisymmetric vortices are uncommon, the threshold for vortex-center identification was reduced to 0.9. The vortex boundary is defined by the $\varGamma _{2}$ function. The threshold values of $|\varGamma _{2}|$ are typically in the range 0.6–0.7, i.e., approximately $2/\pi $ (see, e.g., Graftieaux et al. 2001). From Giagkiozis et al. (2018)

An illustrative example of vortex identification using the $\varGamma $-functions method applied to the velocity field obtained using LCT of features in maps of the continuum intensity near the spectral line Fe i 6302.5 Å is shown in Fig. 1. This method has helped to identify larger numbers of photospheric vortices as determined from radiative intensity maps with much shorter lifetimes than previously reported (see Giagkiozis et al. 2018, and Sect. 6.2 for further details).

### Objective Lagrangian Vortex Detection

Mapping plasma flows is useful for the identification of coherent structures (CS) such as solar vortices of different types. The Lagrangian-averaged vorticity deviation (LAVD) method, which can be applied to uncover the material surfaces^3^ influencing the plasma and organizing the flow into the observed rotational/vortical patterns where the dynamics is dominated by vorticity, was developed by Haller et al. (2016). The authors define the coherent vortices objectively, i.e., by using the LAVD field

20 $$ \mathrm{LAVD}^{t_{0}+\tau}_{t_{0}}(\boldsymbol{x}_{0})= \int ^{t_{0}+ \tau}_{t_{0}}\left |\boldsymbol{\omega} (\boldsymbol{x}(t),t)- \langle \boldsymbol{\omega}(t) \rangle \right |\mathrm{d}t. $$

Here, $\tau $ is a given time interval. The choice of $\tau $ depends on the problem being studied, i.e., $\tau $ can be the lifetime of the vortex or time interval where interesting flow dynamics might take place. The local maxima of the LAVD field provide the candidates for centers of vortices. Furthermore, these local maxima remain unchanged under time-dependent rotations and translations of the coordinate frame. The vortex boundary is identified as the outermost convex closed contour (2D) or isosurface (3D) of the LAVD field. Physically, the vortex boundary defined by LAVD identifies the material surface where the particles experience twice the intrinsic dynamic rotation angle generate by the relative rotation tensor. The convex criterion is imposed to avoid wrong detections and also due to the fact that stable vortices tend to present convex shape. The LAVD method has been applied both to observations (Silva et al. 2018) and to MURaM simulations (Silva et al. 2020, 2021). The Lagrangian vortex definition becomes a Eulerian vortex definition by applying the limit of zero advection time ($\tau \rightarrow 0$). The Instantaneous Vorticity Deviation (IVD) method, which is based on this limit, can be used for the identification of vortices with short lifetimes. The IVD is defined as:

21 $$ \mathrm{IVD}\left ( \boldsymbol{x},t \right ) :=| \boldsymbol{\omega} \left (\boldsymbol{x},t\right ) - \langle{ \boldsymbol{\omega}}\left (t \right )\rangle |. $$

Here, $\langle \boldsymbol{\omega}(t) \rangle $ corresponds to the instantaneous spatial mean of the vorticity.

IVD-based vortices offer a systematic and fully frame-invariant way of tracking coherent velocity features that are consistent in time with coherent material vortices. This makes these Eulerian vortices and vortex centres a suitable, automated tool for deriving the vortex population within turbulent flow data. The deficiency of this method is that it is prone to noise and small temporal scale perturbations.

The Instantaneous Averaged Current Deviation method (IACD; Rempel et al. 2019; Silva et al. 2021) is conceptually similar to LAVD, but is applied to the magnetic current density field. This method is less prone to noise. Using this method, it is possible to identify the regions where the magnetic topology defines a coherent magnetic flux tube.

To avoid the possible false vortex detection and select the true vortices, Silva et al. (2018) proposed to apply the geometric verification of the streamlines of the displacement vector. The method ($d$ parameter) is based on the analysis of the particles displacement that (at the initial time) are located at every grid point and then re-positioned (e.g. advected) by the velocity flows during the given time interval.

### Finite Time Lyapunov Exponent Analysis

**Fig. 2:**
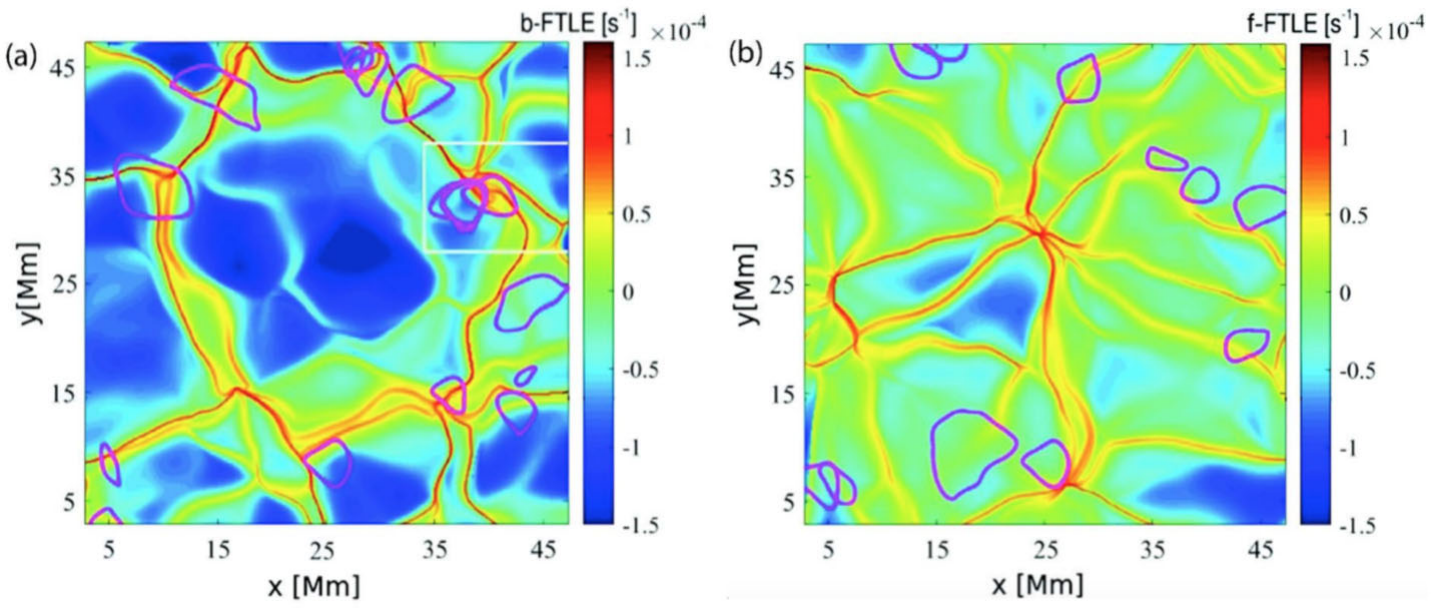
Demonstration of the FTLE analysis applied to 22 hours of solar disk centre continuum intensity observations by *Hinode*. Panels (**a**) and (**b**) show attracting and repelling flow regions in the solar photosphere, respectively. The detected vortex boundaries are marked in magenta. Reprinted figure with permission from Chian et al. (2020), ©APS

As mentioned above, material surfaces that influence the plasma and organize the flow into the observed patterns, can be uncovered with the methodology of Lagrangian coherent structures. However, the visual analysis of patterns of the plasma flow, i.e., velocity distributions, provides only limited information on the flow structure and its dynamics. The Finite Time Lyapunov Exponent method (FTLE; see Shadden et al. 2005) can be used to quantitatively characterise the amount of stretching about the particles’ trajectory. The panels of Fig. 2 depict attracting (a) and repelling (b) regions on the solar surface. These patterns were identified, respectively, by means of the backward (b-FTLE) and forward (f-FTLE) methods, characterizing the amount of stretching about the particles’ trajectory, which precisely defines the dynamics of the flow. The material surfaces define the dynamics of the flow in each region. Both b-FTLE and f-FTLE areas act as barriers to flows, i.e., they cannot be crossed by the tracers. The transport can only happen across saddle points, i.e., the regions where the b-FTLE and f-FTLE cross each other.

The application of combined Lagrangian methodologies provides a powerful new tool in solar physics which has recently been successfully applied to large-scale events (Roudier et al. 2021). Importantly, the theory of Lagrangian coherent structures provides the necessary framework for tracking energy transport during the lifetime of coherent plasma structures, even for plasmas which are in a highly dynamic and nonlinear state, e.g., intergranular turbulence, or, for larger structures, e.g., solar flares and coronal mass ejections (CMEs).

### Detection Criteria Based on the Velocity Gradient Tensor

A large family of criteria for vortex identification are based on the velocity gradient tensor $\mathcal{U}$, introduced in Sect. 3.3, and its eigen-analysis. The velocity gradient tensor can be decomposed into a symmetric and an anti-symmetric tensor, $\mathcal{U} = S + \varOmega $, where $S_{ij} = \frac{1}{2}\left ( \partial _{j} v_{i} + \partial _{i} v_{j} \right )$ and $\varOmega _{ij} = \frac{1}{2}\left ( \partial _{j} v_{i} - \partial _{i} v_{j} \right )$. In particular, $\varOmega $ is in one-to-one correspondence with vorticity $\boldsymbol{\omega}$ and is therefore called the vorticity tensor, while $S$ is the rate-of-strain tensor.

As we will see in the following subsections, most of the information carried by the velocity gradient tensor comes with its eigenvalues, $\lambda _{i}$, which can be computed through the characteristic equation

$$ \det \left ( \mathcal{U} - {\mathrm{I}}\lambda \right ) = 0\,, $$

that leads to

22 $$ \lambda ^{3} + P \lambda ^{2} + Q\lambda + R = 0\,, $$

where $P = -{\mathrm{Tr}}(\mathcal{U})$, $Q = \frac{1}{2}\left ({\mathrm{Tr}}(\mathcal{U})^{2} - {\mathrm{Tr}}( \mathcal{U}^{2})\right )$, and $R = -\det (\mathcal{U})$ are three invariants of the velocity gradient tensor $\mathcal{U}$.

#### $\varDelta $-Criterion

Chong et al. (1990) proved that a vortex core is characterized by the complex eigenvalues of the velocity gradient tensor, since those values imply a closed or spiraling streamline pattern in a co-moving reference frame. The $\varDelta $-criterion is the straight-forward application of this consideration.

One can prove from Eq. (22) that $\mathcal{U}$ can have either three real or one real and two complex conjugate eigenvalues (Chong et al. 1990). The condition to have complex eigenvalues is therefore given by the discriminant of Eq. (22),

23 $$ \varDelta = \left (\frac{\tilde{Q}}{3}\right )^{3} + \left ( \frac{\tilde{R}}{2}\right )^{2}\,, $$

where $\tilde{Q} = Q - P^{2}/3$ and $\tilde{R} = R + 2P^{3}/27 - PQ/3$. For incompressible flows, $P = -{\mathrm{Tr}}(\mathcal{U}) = 0$, hence $\tilde{Q}=Q$ and $\tilde{R}=R$. If $\varDelta > 0$, the velocity gradient tensor has two complex conjugate eigenvalues and the flow describes a vortex at that point.

#### $Q$-Criterion

The $Q$-criterion has been proposed by Hunt et al. (1988) and it is directly related to the second invariant of the velocity gradient tensor appearing in Eq. (22), $Q = \frac{1}{2}\left ({\mathrm{Tr}}(\mathcal{U})^{2} - {\mathrm{Tr}}( \mathcal{U}^{2})\right )$, which can be rewritten as

24 $$ Q = \frac{1}{2}\left ( \lVert \varOmega \rVert ^{2} - \lVert S \rVert ^{2} \right )\,, $$

where $S$ and $\varOmega $ are the symmetric and anti-symmetric versions of $\mathcal{U}$ defined above, and $\lVert \cdot \rVert $ denotes the Frobenius norm. Physically, $Q$ relates the strength of the shear strain to the vorticity rate: points with positive values of $Q$ are therefore identified as part of a vortex. In practice, one usually defines a threshold value $Q_{t}$ and defines vortex regions those for which $Q > Q_{t}$. Additionally, one also often requires the presence of a pressure minimum to be identified as a vortex core. The selection of the $Q_{t}$ threshold requires some trial and error with varying orders of magnitude. High/low values of $Q_{t}$ may result in, respectively, limited/excessive numbers of vortical structures (see also Fig. 32 for an example of appropriate values of $Q$ normalized to the local plasma-$\beta $).

The $Q$ and the $\varDelta $ criteria are related via Eq. (23). For an incompressible fluid, $Q > 0$ implies $\varDelta > 0$. However, the inverse is not true because of the second term, $(R/2)^{2}$. Therefore one can conclude that the $Q$-criterion is more restrictive than $\varDelta > 0$ (Jeong and Hussain 1995).

#### $\lambda _{2}$-Criterion

Jeong and Hussain (1995) realised that a criterion based on pressure minima can fail to identify a vortex core mainly because of two reasons: unsteady strains and viscous effects. Hence, they formulated the $\lambda _{2}$-criterion by neglecting these two effects on the evaluation of the pressure minimum.

By taking the gradient of the Navier-Stokes equations, one obtains

$$ \boldsymbol{\nabla}{\left (\frac{\mathrm{d}}{{\mathrm{d}}t}{ \boldsymbol{v}}\right )} = -\frac{1}{\rho}\boldsymbol{\nabla}{\left ( \boldsymbol{\nabla}{p}\right )} + \nu \boldsymbol{\nabla}{\left (\varDelta \boldsymbol{v}\right )}\,, $$

where $\varDelta = \boldsymbol{\nabla}^{2}$ denotes the Laplace operator. This can be rewritten in terms of the $S$ and $\varOmega $ components of the velocity gradient tensor as

25 $$ \frac{\mathrm{d}}{{\mathrm{d}}t}S - \nu \varDelta S + \varOmega ^{2} + S^{2} = - \frac{1}{\rho}\boldsymbol{\nabla}{\left ( \boldsymbol{\nabla}{p}\right )} \,. $$

On the left-hand side, the first term represents unsteady straining while the second one describes viscous effects, therefore one shall not consider them. On the right-hand side we find the Hessian matrix of the pressure $p$, whose eigenvalues characterize its stationary points. More precisely, a point is a local pressure minimum if $\boldsymbol{\nabla}{\left (\boldsymbol{\nabla}{p}\right )}$ has two positive eigenvalues. Then, from Eq. (25), one can define a vortex region as those points for which $\varOmega ^{2} + S^{2}$ has two negative eigenvalues. Considering that $\varOmega ^{2} + S^{2}$ is a symmetric matrix and therefore has real eigenvalues only, one can order them in the following way: $\lambda _{1} \geq \lambda _{2} \geq \lambda _{3}$. Then, a vortex core is equivalently defined by the requirement that $\lambda _{2} < 0$, hence the designation of $\lambda _{2}$-criterion.

As a final remark, let us notice that from Eq. (24) one can also write the $Q$-criterion as

$$ Q = \frac{1}{2}{\mathrm{Tr}}\left ( \varOmega ^{2} - S^{2} \right ) = - \frac{1}{2} \left ( \lambda _{1} + \lambda _{2} + \lambda _{3} \right )\,. $$

Hence the $Q$-criterion can be interpreted as the average excess of vorticity rate over the shear strain in all directions, while the $\lambda _{2}$-criterion only considers one eigen-plane (Jeong and Hussain 1995).

#### Swirling Strength or $\lambda _{\mathrm{ci}}$-Criterion

The swirling strength criterion is defined as the imaginary part of the complex eigenvalues of the velocity gradient tensor (Zhou et al. 1999). It can be seen as an improved version of the vorticity since it is not affected by shear flows (see Sect. 3.3 for further details).

**Fig. 3:**
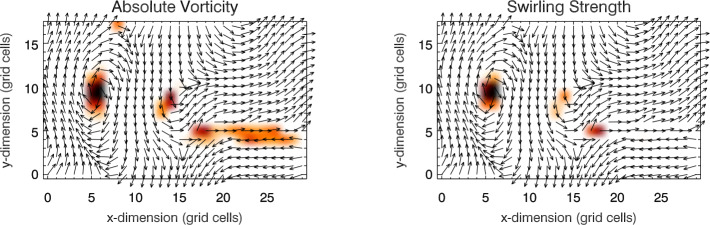
Top 5% of absolute vorticity (left panel) and swirling strength (right panel) within a simulated flow field from a MURaM simulation described in Yadav et al. (2020, 2021). While vortex regions are correctly identified, a region of extended vorticity at $x=16...27,~y=5$ corresponds to a shear flow, which can be mistaken as vortical flow when looking at the vorticity map alone

**Fig. 4:**
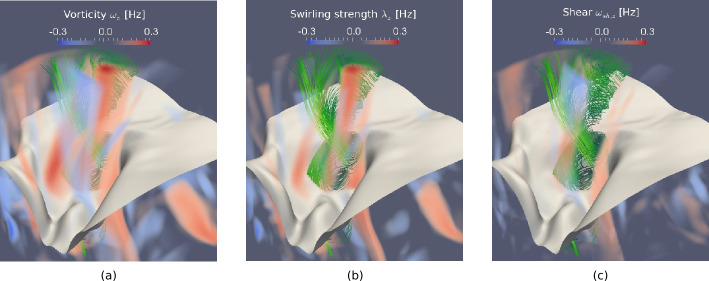
Vertical component of the vectors of vorticity (**a**) and swirling strength (**b**) in a small portion ($600 \times 600 \times 1800~{\text{km}}^{3}$) of a CO^5^BOLD simulation box. The plasma streamlines are rendered in green, while the gray sheet represents the optical surface $\tau _{500}=1$. Panel (**c**) represents the vertical component of the shear strength vector, $\boldsymbol{\omega}_{\mathrm{sh}} = \boldsymbol{\omega} - \boldsymbol{\lambda}$. Credit: Canivete Cuissa and Steiner (2020), reproduced with permission ©ESO

Figure 3 shows a comparison between the vorticity and swirling strength criteria of a two-dimensions flow field taken from a simulation. While the vortex regions are correctly identified, an extended region of shear flow can be mistaken as vortical flow when looking at the vorticity criterion alone. An example of the application of the swirling strength criterion to simulation data in three-dimensional space and its comparison to vorticity is given in Fig. 4. The three-dimensional rendering shows that the swirling strength successfully identifies the vortex depicted by the green streamlines, while according to vorticity there should be two counter-rotating vortices close to each other and more small-scale structure in the background. The structure that is not detected by the swirling strength is most probably due to shear flows. Panel (c) shows the shear strength $\boldsymbol{\omega}_{\mathrm{sh}} = \boldsymbol{\omega} - \boldsymbol{\lambda}$, which is a proxy for the presence of shear flows, and, as we can see, they are ubiquitous in that portion of the simulation box.

Using the same mathematical description, Battaglia et al. (2021) defined the magnetic swirling strength criterion, $\lambda ^{\mathrm{B}}$, which result from the eigenanalysis of the magnetic field gradient tensor, $\mathcal{M}_{ij} \equiv \partial _{j} B_{i}$. This quantity is not intended to detect swirling motions, but to identify twists in magnetic field lines. As for the swirling strength, one can define a magnetic swirling vector, $\boldsymbol{\lambda}^{\mathrm{B}} = \lambda ^{\mathrm{B}} \boldsymbol{u}_{\mathrm{r}}^{ \mathrm{B}}$, where $\boldsymbol{u}_{\mathrm{r}}$ is the real eigenvector of ℳ. Consequently, this vector provides the strength, the direction, and the orientation of the twist in the magnetic field.

#### Enhanced Swirling Strength

In order to correctly identify a vortex, Chakraborty et al. (2005) propose three requirements: the criterion should be Galilean invariant, the flow should be swirling in a reference frame moving with the vortex and the orbits of the fluid particles within the vortex should be compact. The swirling strength criterion fulfills the first two conditions, but yields no information regarding the compactness of the trajectories. This requirement is necessary to recognize the vortex from a dynamical point of view: If the fluid particles do not remain bounded in time, one can not define that structure as a coherent one.

The enhanced swirling strength criterion (Chakraborty et al. 2005) is an attempt to fulfill the three requirements using the swirling strength. Let us consider the motion of a fluid particle in the vortex plane, namely the plane spanned by the real eigenvectors ($\boldsymbol{u}_{ \mathrm{cr}}$, $\boldsymbol{u}_{\mathrm{ci}}$). Using Eq. (16) one can prove that after $n$ revolutions in that plane, two particles initially separated by $r_{0}$ will be separated by $r_{f}$ according to the following expression

$$ r_{f} = r_{0}\exp{\left ( 2\pi n \frac{\lambda _{\mathrm{cr}}}{\lambda _{\mathrm{ci}}} \right )}\,. $$

The ratio $\lambda _{\mathrm{cr}}/\lambda _{\mathrm{ci}}$ measures the spatial extent of the swirling motion, and is therefore named the inverse spiralling compactness ratio. If $\lambda _{\mathrm{cr}}/\lambda _{\mathrm{ci}} = 0$, the fluid particles follow perfectly circular trajectories, while if the ratio is positive (or negative) they are spiraling outwards (or inwards) in the vortex plane. Hence, Chakraborty et al. (2005) proposed to define vortex regions those for which the following criteria are satisfied

$$ \textstyle\begin{cases} \lambda _{\mathrm{ci}} \geq \epsilon \\ \lvert \frac{\lambda _{\mathrm{cr}}}{\lambda _{\mathrm{ci}}} \rvert \leq \delta \,, \end{cases} $$

where $\epsilon $ and $\delta $ are two positive thresholds. The first criterion makes sure that there exists a finite amount of swirling strength, the second that the particle trajectories remain compact.

### Rortex or Liutex

Local vortex-identification criteria, such as the ones derived from the velocity gradient tensor (see Sect. 4.6), are commonly used because of their simplicity and because they are not affected by the presence of shear layers. However, contamination due to rotation intrinsic shear (see below for a definition) is still possible.

Xu et al. (2019) proved, using the real Schur decomposition theorem, that for any velocity gradient tensor $\boldsymbol{\nabla}\boldsymbol{v}$ with one real and two complex conjugate eigenvalues, there exists an orthogonal matrix $\boldsymbol{Q}$ and a transposed quasi-triangular form $\boldsymbol{\nabla}\boldsymbol{V}$ such that

26 $$ \boldsymbol{\nabla}\boldsymbol{v} = \boldsymbol{Q}\, \boldsymbol{\nabla} \boldsymbol{V}\, \boldsymbol{Q}^{\mathrm{T}}\,, $$

where the orthogonal matrix $\boldsymbol{Q}$ represents a rotation operator. It transforms the original reference frame to a new one with the fluid rotation axis $\boldsymbol{e}_{\mathrm{r}}$ parallel to the new $\hat{z}$-axis. The unit vector $\boldsymbol{e}_{\mathrm{r}}$ corresponds to the normalized eigenvector associated with the real eigenvalue of $\boldsymbol{\nabla}\boldsymbol{v}$. Indeed, in the rotated reference frame, the velocity gradient tensor $\boldsymbol{\nabla}\boldsymbol{V}$ can be expressed as

27 $$ \boldsymbol{\nabla}\boldsymbol{V} = \begin{bmatrix} \lambda _{\mathrm{cr}} & -\phi & 0 \\ \phi + \varepsilon & \lambda _{\mathrm{cr}} & 0 \\ \xi & \nu & \lambda _{\mathrm{r}} \end{bmatrix} \,, $$

where $\lambda _{\mathrm{cr}}$ and $\lambda _{\mathrm{r}}$, already encountered in Sect. 4.6, are the real part of the complex conjugate eigenvalues and the real eigenvalue, respectively, and are related to the stretching and compressing components of the flow. The purely rotational component of the velocity gradient tensor is represented by $\phi $, while $\varepsilon $, $\xi $, and $\nu $ are the shearing parts. This shows that, even when shear layers are absent (i.e. $\xi , \nu = 0$), an intrinsic rotational shear ($\varepsilon $) can contaminate the criteria based on the eigenvalues of the velocity gradient tensor. Such an intrinsic rotational shear is for example present in Lamb-Oseen vortex models.

The Rortex criterion (also known as “Liutex”), defined as $R = 2\phi $, was introduced by Tian et al. (2018) and Liu et al. (2018), and it measures the strength of pure local rotation without shearing contamination. Therefore, it is the only quantity that can be trusted to infer properties of the rotational flow, such as the rotation period. The Rortex has also a vector form, $\boldsymbol{R} = R \boldsymbol{e}_{\mathrm{r}}$, which allows for the three-dimensional characterization of the rotational flow.

The original derivation proposed by the authors relies on the calculation of the orthogonal matrix $Q$, which can be tortuous and computationally expensive (see, e.g. Gao and Liu 2018). However, Wang et al. (2019) and Xu et al. (2019) derived an explicit and simple expression for the computation of the Rortex criterion, which reads as follows,

28 $$ R = 2\phi = \boldsymbol{\omega}\cdot \boldsymbol{e}_{ \mathrm{r}} - \sqrt{( \boldsymbol{\omega}\cdot \boldsymbol{e}_{ \mathrm{r}})^{2} - 4 \lambda _{ \mathrm{ci}}^{2}}\,, $$

where $\lambda _{\mathrm{ci}}$ is the imaginary component of the complex conjugate eigenvalues of the local velocity gradient tensor.

The Rortex criterion has been recently applied in the context of vortices in the solar atmosphere by Canivete Cuissa and Steiner (2022). They show that the Rortex criterion is the most reliable criterion for the extraction of physical information from vortical flows. Moreover, they use the Rortex criterion and the properties of the local velocity field to estimate the center of rotation of fluid parcels showing some degree of rotation in the neighboring flow. Vortices can then be identified by clusters of these estimated centers of rotation (or estimated vortex centers, EVCs), since all fluid parcels belonging to a vortex share a common curvature center.

### Morphological Methods

The derivation of the velocity field can be challenging, sometimes highly uncertain, or even impossible and the calculation of vorticity-based parameters computationally consuming. In such cases, approaches based on the morphological and geometrical characteristics of vortex flows are preferable. Morphology-based detection methods are frequently used in various scientific and engineering areas for the appropriate visualisation of velocity vector fields.

Sadarjoen and Post (1999) proposed two techniques for the detection of vortices based on the geometric properties of streamlines, applied to datasets of Computational Fluid Dynamics (CFD). The Curvature Centre Method attempts a detection of vortices in two-dimensional velocity vector fields by creating a grid of sample points for which one determines their centres of curvature of the corresponding streamline and finally grouping together areas of high point densities, where the centers of curvature accumulate. The Winding Angle Method selects and clusters together looping streamlines that form vortex areas, each of them approximated by an ellipse whose properties determine vortex-related physical properties such as size and orientation.

**Fig. 5:**
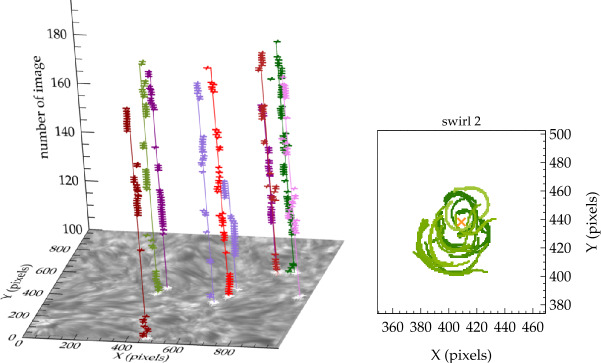
Left panel: Extracted swirl-related centers of curvature with the second-level clustering throughout the examined time interval. Right panel: Projected segments of the detected “swirl 2” (corresponding to the green line and points of the left panel) over the entire examined time interval and its mean centre (orange cross). Darker/lighter shading indicates earlier/later appearing segments in time. From Dakanalis et al. (2021)

The derivation of high point density areas formed by highly-curved features was recently implemented in a solar physical context by Dakanalis et al. (2021). They developed an automated chromospheric swirl detection method to exploit the spiral-like observational signatures of plasma motions in the higher layers of the solar atmosphere (e.g., observations in the spectral line core of $\text{H}\alpha $ and Ca ii 8542 Å). This detection technique tracks, simultaneously in space and time, highly-curved structures that have been traced in high-contrast, edge-enhanced (filtered) images. The process includes several stages such as image pre-processing for enhancing the edges of the features under study, local and adaptive thresholding for maintaining structures fainter than their local environment, and sequential intensity-based tracing of relevant segments. Curvature-related criteria and a minimum curvature radius are used to retain only highly-curved segments in each image, whose centers of curvature are clustered together with the use of an unsupervised machine learning technique to form high point-density vortex-related areas characterized as swirl candidates. The final classification of the acquired swirl candidates to detected swirls is performed with a second level clustering in time. It involves the use of observationally-driven temporal evolution criteria applied to the two-dimensional projection of all acquired swirl-candidate centres (see Fig. 5).

## Formation of Intergranular and Atmospheric Vortex Flows

The plasma flows at the solar surface (see footnote 1 for a definition) are in general divergent on the surface of convection cells, which are observed as bright granules. At the cell boundaries, the flows from neighbouring cells converge and are redirected downwards back into the convection zone, forming a network of darker intergranular lanes. The plasma can carry a net angular momentum that may arise randomly or may be induced by the differential rotation of the Sun (Bonet et al. 2010). It is usually rather weak until the plasma starts to densify as it cools and sinks down again in the intergranular lanes. There, conservation of angular momentum carried by the cooled plasma results in the formation of vortex flows that extend from the low photosphere into the top layer of the convection zone. This effect is also known as *bathtub effect* (Nordlund 1985) as it reminds of the swirling motion of water in the bathtub outlet. It is an integral part of hydrodynamic flows in stratified media and is thus expected in the context of stellar surface convection. In fact, such flows as a result of the bathtub effect had already been seen in early hydrodynamic simulations of solar surface convection by Nordlund (1985) who refer to these flows as *inverted tornado*. It should be noted that vortex formation via the bathtub effect works most efficiently at the vertices of intergranular lanes and in the absence of strong magnetic fields (see, e.g., Porter and Woodward 2000; Kitiashvili et al. 2012a; Steiner and Rezaei 2012; Wedemeyer and Steiner 2014, and references therein). These hydrodynamic vortex flows in the low photosphere are referred to as *intergranular vortex flows* (IVF) by Wedemeyer and Steiner (2014, cf. Sect. 2). Such IVFs occur also frequently in the simulations by Moll et al. (2011b). The horizontal extent of IVFs is naturally set by the size of intergranular lanes but their visible size has been estimated by Calvo et al. (2016) to be below 0.1 arcsec, which makes them difficult to be resolved with current solar telescopes. Photospheric vortex flows on different spatial scales larger than an intergranular lane have been observed (e.g., Brandt et al. 1988; Bonet et al. 2008, 2010; Vargas Domínguez et al. 2011) but are the result of the conservation of angular momentum for flows on accordingly larger spatial scales.

**Fig. 6:**
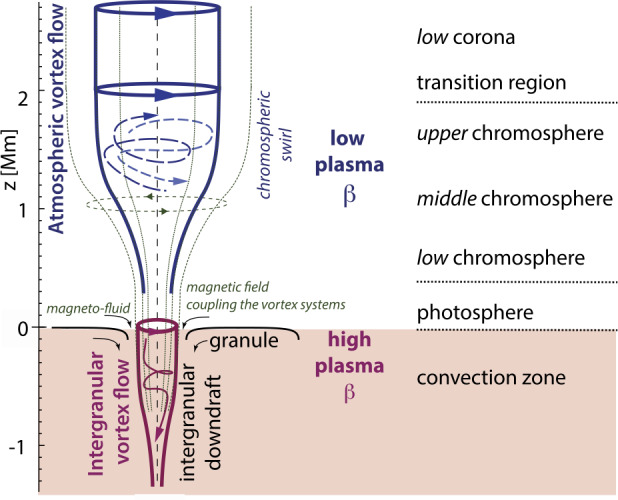
Schematic representation of the atmospheric vortex flow (AVF), also known as “magnetic tornado” forming in low plasma-$\beta $ conditions, on top of a photospheric intergranular vortex flow (IVF) that forms in denser, lower layers of high plasma-$\beta $ and drives the AVF above (see text for more information). The magnetic field (thin line) couples the two vortex flows. From Wedemeyer and Steiner (2014) by permission of Oxford University Press on behalf of the Astronomical Society of Japan

Wedemeyer and Steiner (2014) suggest that an IVF is an essential prerequisite for the formation of a *magnetic tornado*, a term coined by Wedemeyer-Böhm et al. (2012) and also referred to as *atmospheric vortex flow* (AVF) by Wedemeyer and Steiner (2014). The second ingredient needed for the formation of a *magnetic tornado* is a magnetic flux concentration that becomes spatially co-located with an IVF. Since the magnetic field is essentially frozen into the highly conductive plasma, the IVF then starts to rotate the photospheric part of the magnetic field concentration. The magnetic field itself mediates the rotation into the low plasma-$\beta $ domain in the upper layers ($\beta $ expressing the ratio of the plasma pressure to the magnetic pressure), where the situation is reversed. There, the plasma follows the rotating magnetic field structure, which in turn produces a (driven) atmospheric vortex flow in the chromosphere (see Fig. 6). It is this upper part of the magnetic tornado that leaves observable imprints in chromospheric diagnostics such as the infrared triplet lines of singly ionized calcium. This type of observable vortex flow is referred to as *chromospheric swirl* (Wedemeyer-Böhm and Rouppe van der Voort 2009).

Battaglia et al. (2021) find this magnetic mediation of rotation to proceed in the form of torsional Alfvén pulses that propagate from the surface layer of the convection zone to the chromosphere, thereby causing the AVF. They are less conclusive about the origin of the pulses. While they do not exclude IVFs as the origin, they find that magnetic baroclynic and tensions forces are as important as hydrodynamical effects in vortex forming regions. They also conjecture that a mere deformation of a magnetic flux concentration may be sufficient to trigger a torsional pulse, or that even magnetic reconnection with nearby flux concentrations of opposite polarity may play a role.

The overall lifetime of a magnetic tornado depends on the interaction of the driving mechanism such as an IVF with the photospheric footpoint of the magnetic field structure. As long as an IVF coincides with the footpoint, or when a sequence of Alfvén pulses of unidirectional rotation is excited in some other way, the chromospheric vortex flow can in principle persist. This also means a continuous spectrum of lifetimes can be expected, ranging from only partial rotations to several revolutions lasting for several minutes. Clearly, the dynamic timescales of the photospheric flow field, which is connected to the typical lifetimes of granules, determines how long a driver can exist and thus how long the chromospheric counterpart is rotating.

The formation of magnetic tornadoes strongly depends on the magnetic field environment. On the one hand, enough magnetic flux must be present so that significant flux concentrations can be formed but, on the other hand, too much magnetic flux and thus too high magnetic field strengths and filling factors in the chromosphere hamper the rotation of the chromospheric parts of the associated magnetic flux concentrations. Likewise, an open field topology seems to facilitate unimpeded rotation of magnetic tornadoes. In this sense, coronal holes seem to provide favorable conditions (see Sect. 6). Numerical simulations with an average magnetic field strength of $|B_{0}| =50~\text{G}$ seem to produce an increased number of magnetic tornadoes as compared to simulation runs with lower and higher $|B_{0}|$ values. Also magnetic fields of opposite polarity in the environments of a magnetic flux concentration may play a role and could possibly, by way of a photospheric reconnection event, excite torsional Alfvén pulses.

Steiner et al. (2010) found from observations in combination with numerical simulations both further detailed in Sects. 6.2 and 7.2, respectively that signatures of horizontally-oriented vortex tubes frequently appear at the boundaries of granules. The driving mechanism for this type of vortex flow is not magnetic nor a consequence of angular momentum conservation but very probably the hydrodynamic baroclinic effect (see fourth term on the right hand side of Eq. (7) or second term of Eq. (8)). It produces vorticity with a rate of $\dot{\omega}_{\mathrm{baroclinic}} = -\nabla (1/\rho ) \times \nabla p_{\mathrm{gas}}$ whenever the gradients of density and gas pressure are not parallel to each other. However, since both these gradients are in a gravitationally stratified atmosphere close to vertically directed, their cross product, and therefore the baroclinic vorticity, must be close to horizontal, which is the reason for the horizontal orientation of these vortex tubes. We note, however, that a rigorous analysis of radiation (magneto-)hydrodynamic convection simulations with regard to the driving mechanism of horizontal vortex tubes is presently still lacking. More on this type of vortex flows can be found in Sects. 6 and 7.

Concluding on the formation of vortices on the solar surface and in the atmosphere, we note that while the bathtub effect is initiated by random motion, other effects, like the baroclinic effect, generate vorticity. Currently available studies mainly focus on the magnetic origins of vorticity or on disentangling shear flows from vortex motions. Often, the vorticity equations (i.e., Eqs. (7) and (8)) are studied in the Lagrangian form, or, if not, the advection term is not explicitly analyzed. Presently, there exists no systematic study of vorticity transport in the solar upper convection zone and the photosphere. The precise origin of vortical motions in the solar atmosphere is often unknown. It requires a careful study of the various source terms of vorticity or swirling strength (Eq. (7) and Eq. (17), respectively) including the transport of vorticity or swirling strength from the convection zone into the photosphere. These are questions to be properly addressed in future works.

## Observations of Vortex Motions

In this section, an overview of observations of vortex motions in different layers of the solar atmosphere, from the photosphere up to the lower corona, and their observational properties is provided. Although this review mainly concentrates on small-scale vortex motion, a short discussion on observations of giant tornadoes is also included Sect. 6.4. Moreover, we also discuss the few helioseismic observations concerning large-scale, subsurface quiet-Sun vortex flows in Sect. 6.1. Observational signatures of vortex-related oscillations and waves are reserved for Sect. 8.1.

While Sect. 2 highlights in a concise manner the diversity of names given in the literature to the many vortical phenomena on the Sun, the previous section provides details on the physics of these phenomena and this and the following Sect. 7 give more details on the observations and simulations of such vortical phenomena, keeping the originally provided terminologies. As already mentioned in Sect. 2, similarities and differences between the various phenomena are discussed in Sect. 10.1 where recommendations on the nomenclature are also given.

### Helioseismic Observations

The subsurface vortex flows play a key role in many processes of solar dynamics and activity, from small-scale phenomena to the global Sun magnetism. The development of local helioseismology diagnostics such as time-distance helioseismology, ring-diagram analysis, and acoustic holography has opened new perspectives for understanding the solar subsurface dynamics and led to important discoveries. However, we note that such techniques can, for the moment, only be applied on large-scale subsurface vortex motions. This is due to theoretical limitations as the short lifetimes of spatially small vortices would require the use of short wavelength modes and short timeseries with the consequence of a low signal to noise ratio, detrimental for inferring small-scale vortical flows in helioseismic data. As an example of granular-scale solar $p$-mode analysis, we refer to Roth et al. (2010).

Helioseismic techniques have been mainly used for large-scale subsurface vortex flows in active regions (Zhao et al. 2001) that are often associated with the dynamics of fast-rotating sunspots (Zhao and Kosovichev 2003). There have been extensive analyses of active-region subsurface flows and their effect on large-scale flows and the global circulation of the Sun (e.g. Komm et al. 2011a; Kosovichev et al. 2018), their differences in flaring and non-flaring active regions (Komm et al. 2011b), studies of the solar vorticity on the global Sun scale (Zhao and Kosovichev 2004), and the association of large-scale vortex flow patterns with Rossby waves (Proxauf et al. 2018). However, the discussion of such large-scale vortex motions, mainly in active regions, is out of the scope of this paper and is not going to be further discussed.

In the quiet Sun, vortex flows were studied by Langfellner et al. (2015). Accordingly, solar supergranules exhibit a hemisphere-dependent preferred sense of rotation as a result of the Coriolis force acting on diverging horizontal flows. This rather weak effect was detected by measuring the vertical flow vorticity of the average supergranule at different latitudes, both for outflow and inflow regions. The vertical vorticity was measured by Langfellner et al. (2015) with two different techniques, time-distance helioseismology (TD) and local correlation tracking (LTC) of granules. They were applied on (corrected for center-to-limb variations) Doppler velocity and intensity images, respectively, from the Helioseismic and Magnetic Imager (HMI) on board the Solar Dynamics Observatory (SDO). A high correlation was found at large spatial scales between derived 8-hour TD and LCT maps of vertical vorticity. Tangential, vortical flows of $\sim 10~\text{m}\,\text{s}^{-1}$ in the clockwise direction were derived at 40 degrees latitude that are associated with the average supergranular outflow, while similar vortical flow velocity magnitudes were derived in inflow regions but in the anticlockwise direction. These velocities are much smaller than the average $300~\text{m}\,\text{s}^{-1}/200~\text{m}\,\text{s}^{-1}$ radial diverging flow component within outflow/inflow regions. Between −60 and 60 degrees, TD and LCT results are in excellent agreement. The LCT method revealed a full width at half maximum (FWHM) of the vorticity peak equal to 13 Mm (8 Mm) for outflows (inflows) that is larger than the 3-Mm spatial resolution of the LCT measurements. The vorticity peak itself, at 40 degrees latitude, is about $4 \times 10^{-6}~\text{s}^{-1}$ (clockwise) in outflows and about half the respective value in inflows (anticlockwise).

### Photospheric Observations

Almost all of the existing observational studies of vortex flows at the photospheric level are based on the visual inspection of the motion of magnetic bright points (hereafter BPs) or on tracking passively advected tracers and monitoring their evolution.

Horizontal velocity flows derived with LCT from observation in the continuum radiation of solar granulation at the Pic du Midi Observatory suggested an excess of vorticity in intergranular lanes, while several small-scale, sub-arcsecond ($\sim 0.5~\text{Mm}$) bright points located at supergranular boundaries were found to rotate about each other (Wang et al. 1995). These results were confirmed by Pötzi and Brandt (2005, 2007), using a similar analysis applied to granulation observations obtained with a broadband filter centred at 4690 Å with the Swedish Solar Telescope (SST) on La Palma. They too concluded that matter sinks down within vortices. Even earlier, a single vortex flow was observed by Brandt et al. (1988). The observations were obtained with the (0.5-m at the time) SST and a broad band filter centred at a wavelength of 4696 Å. The observed vortex flow was relatively large with a diameter of $\sim 5~\text{Mm}$ and persisted for the 1.5 hour duration of the movie. The horizontal velocity field was derived by LCT and an average flow speed of $0.67~\text{km}\,\text{s}^{-1}$ was found. A root-mean-square (r.m.s.) of the spectroscopically measured line-of-sight (LOS) velocities of $\sim 0.2$ to $0.3~\text{km}\,\text{s}^{-1}$ and a central vorticity of $\simeq 1.4\times 10^{-3}$ were measured. Large-scale, persistent vortex flows at supergranular junctions were also found by Attie et al. (2009). To derive the photospheric flows, they applied the ball tracking technique (Potts et al. 2004) to time series of continuum images obtained with the Solar Optical Telescope (SOT) of the Hinode satellite. Two long-lasting vortex flows were detected, one lasted more than 1 h, the other more than 2 h, while the influence of the flows extended beyond distances of $\sim 7~\text{Mm}$ and $\sim 10~\text{Mm}$, respectively, as measured from the centre of the vortices.

**Fig. 7:**
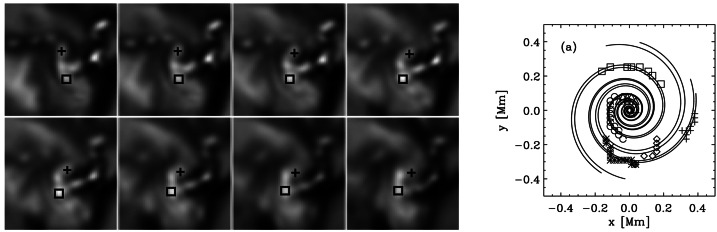
Left panel: Sequence of snapshots (top left to bottom right) separated by 15 s. With symbols are marked two sets of two BPs rotating with respect to each other and depicting a spiral pattern. Right panel: The logarithmic spiral fit (solid lines) for the trajectories of the observed BPs (symbols). From Bonet et al. (2008) ©AAS. Reproduced with permission

Convectively-driven vortex flows at smaller scales were first detected within intergranular lanes by Bonet et al. (2008) as swirling motions of BPs representing magnetic flux concentrations (see Fig. 7). They used high-resolution SST G-band images and, by visually tracing the trajectories of BPs, they found that they follow logarithmic spirals. The area of the swirling region was of the order of $0.5 \times 0.5~\text{Mm}^{2}$, the radius of the curvature was $\sim 100~\text{km}$ and the BPs’ proper motion velocities spanned from 1 to $4~\text{km}\,\text{s}^{-1}$. By identifying swirling motions over the entire FOV of the images and for the full time series that lasted for 31.5 minutes, they reported a mean lifetime of $\simeq 5.1\pm 2.1~\text{min}$ and a space-time density of $\simeq 1.8\times 10^{-3}$ vortices $\text{Mm}^{-2}\,\text{min}^{-1}$ or $\simeq 9\times 10^{-3}$ vortices $\text{Mm}^{-2}$ at any given time on the solar surface. As they argued, these numbers rather represent lower limits.

Bonet et al. (2010) analyzed time series of observations in the magnetic sensitive Fe i 5250.2 Å line obtained with the Imaging Magnetograph eXperiment (IMaX) on board of the 1 m balloon-borne SUNRISE I telescope. They first identified small-scale individual vortices by visual inspection of magnetograms. They then applied the LCT technique of November and Simon (1988) as implemented by Molowny-Horas and Yi (1994) to 5 different observational parameters, i.e., continuum intensity, line minimum intensity, LOS velocity, line width, and longitudinal magnetic field to measure the horizontal motions in sub-fields containing the vortex areas. Using the derived horizontal flow field, they tracked the evolution of passively advected corks (used as tracers) that ended up in sinkholes. They reported a space-time density of $\simeq 3.1 \times 10^{-3}$ vortices $\text{Mm}^{-2}\,\text{minute}^{-1}$ with a mean lifetime of $\simeq 7.9\pm 3.2~\text{min}$. Some of them, however, lasted longer than the 31.6 min time series, while several events showed a recurrent character. They obtained vorticities smaller than $6 \times 10^{-3}~\text{s}^{-1}$ corresponding to a period of rotation of $\sim 35~\text{min}$.

**Fig. 8:**
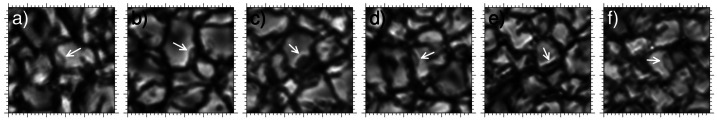
Six examples of granular lanes **a**) to **f**) marked with white arrows. Observations with IMaX on SUNRISE I in the continuum of Fe i 5250 Å. Adapted from Steiner et al. (2010) ©AAS. Reproduced with permission

Steiner et al. (2010) investigated the same IMaX/SUNRISE I data to find from maps of the continuum intensity near the Fe i 5250.2 Å spectral line that granules frequently show lanes at their boundary, composed of a leading bright rim and a trailing dark edge, which move together into the parent granule. Figure 8 shows six examples of them. Additional Dopplergrams indicate in combination with numerical simulations of solar surface convection that these granular lanes are the visible signature of horizontally oriented vortex tubes that form near the boundaries of granules (see Sects. 7.2 and 7.3 for further details on the simulations). We note that these are quite distinct and different from all the other, rather vertically oriented, vortices discussed in this section. Such observational signatures of horizontally-oriented tubes have recently also been reported by Tziotziou et al. (2018) in the wings of the $\text{H}\alpha $ and Ca ii 8542 Å lines depicting granulation (see their Fig. 6).

The observations by Steiner et al. (2010), which included the polarization signal, did not indicate any enhanced magnetic fields at locations of granular lanes. However, Fischer et al. (2020), using spectropolarimetric observations in the Fe i 6173 Å line, detected cases of granular lanes with significant linear polarization located at the aforementioned trailing dark edges. These measurements indicate magnetic field directed along the lane, hence along the vortex tube axes. They were further interpreted with the help of accompanying magnetohydrodynamic numerical simulations, which are discussed in Sect. 7.3.

Balmaceda et al. (2010) analyzed quasi-simultaneously taken ground-based observations from the SST (images in the G-band) and observations from space with instruments on-board the Hinode space observatory (filtergrams in CN and the core of the Ca ii H line with the Broadband Filter Imager (BFI), magnetograms in the Mg i b_2_ line with the Narrowband Filter Imager (NFI), and the Stokes parameters $I$, $Q$, $U$, and $V$ with the spectropolarimeter (SP)). The region observed was the same as the one investigated in the study of Bonet et al. (2008). SP data were inverted using the inversion code LILIA (Socas-Navarro 2001). From this, two-dimensional maps of the magnetic field strength $|\text{B}|$ were constructed. They identified magnetic flux concentrations that are dragged towards the center of convective vortex motions, which they observed in various wavelengths, hence, in different heights in the solar atmosphere. By computing horizontal proper motions, using the LCT technique, they detected two vortex-type events for which all horizontal velocity vectors in the neighborhood converged and which centres appeared to be the draining points for the magnetic flux concentrations. These events lasted for more than 20 min, affecting a circular area of diameter of $\sim 2~\text{Mm}$. They were well-correlated with the locations of bright points seen in G-band and CN images. Using the same dataset, Vargas Domínguez et al. (2015) analyzed a region characterised by vortex-type plasma motions. They followed the evolution of BPs, their intensity variations at various heights, and their magnetic field variations. They found highly dynamic, often fragmenting BPs and intensifying magnetic field strengths resulting from the coalescence of rotating magnetic flux concentrations during their dragging by formed convective vortex motions.

Vargas Domínguez et al. (2011) used the same SST dataset as Balmaceda et al. (2010), more specifically, the two time-series of G-band images. Their analysis is based on the LCT technique, as implemented by Molowny-Horas and Yi (1994), to compute proper motions of features in the observed FOV, and respective computations of the horizontal velocity field divergence. They identified the vortical flows by visual inspection of the horizontal velocity flow-maps, considering as a vortex region the region where velocity vectors change direction and converge towards a central point. They found that vortices were mainly located in the intergranular lanes, were associated with downdrafts, and had a radius of $241\pm 25~\text{km}$. Computing the vorticity of the horizontal flow they obtained a median value of $\sim 2.1 \times 10^{-3}~\text{s}^{-1}$. They also reported an occurrence of vortex-type events in the $69\times 69~\text{arcsec}^{2}$ FOV of $2.8 \times 10^{-2}$ vortices per $\text{Mm}^{2}$ and $3.1 \times 10^{-2}$ vortices per $\text{Mm}^{2}$, or space-time densities of $1.4 \times 10^{-3}$ vortices $\text{Mm}^{-2}\,\text{min}^{-1}$ and $1.6 \times 10^{-3}$ vortices $\text{Mm}^{-2}\,\text{min}^{-1}$ in the two time series, respectively.

Requerey et al. (2017) used two datasets of high quality spectropolarimetric observations from IMaX/SUNRISE I (the same data that were also used by Bonet et al. (2010) and Steiner et al. (2010)) to associate quantitatively mesogranular flows, convectively driven point-like sinks, and small-scale magnetic fields in the quiet-Sun. Via LCT, they obtained the horizontal velocity field and computed the flow divergence and the vertical vorticity in the FOV. By using Lagrange tracers (corks), they identified the mesogranular lanes and the central positions of sinks. They obtained a number density of $6.7 \times 10^{-2}$ converging flows per $\text{Mm}^{2}$. By analyzing the converging flows, they found that some converged radially while others followed a spiral path. The latter were categorized as vortex flows. They detected $2.4 \times 10^{-2}$ vortices per $\text{Mm}^{2}$ and a median absolute vertical vorticity of both clockwise and counterclockwise motions of $1.5 \times 10^{-3}~\text{s}^{-1}$. Using Stokes Inversions based on Response functions (SIR, Ruiz Cobo and del Toro Iniesta 1992), they estimated the vector magnetic field and the LOS velocities. They found that mesogranular lanes and converging flows were associated with long-lived downdrafts and that stronger magnetic fields were preferentially located at sinks. By studying individual events, they showed the important role that sinks can play in the evolution of quiet-Sun magnetic elements, through different processes, i.e., coalescence, cancellation, and fragmentation.

In a follow-up work Requerey et al. (2018) used a 24-hour, uninterrupted sequence of photospheric observations obtained with the narrowband filter imager (NFI) of SOT on-board the Hinode satellite, to investigate the evolution of magnetic network elements and their interaction with vortex flows at a supergranular vertex. NFI provided Stokes $\mathit{I}$ and $\mathit{V}$ filtergrams from which 2D intensity maps, longitudinal magnetograms, and Dopplergrams were constructed. They derived horizontal flows using the LCT technique (as implemented by Molowny-Horas and Yi 1994) in continuum intensity images, magnetograms, and Dopplergrams, and identified regions of downdrafts. They studied the evolution of a persistent vortex flow located at a supergranular vertex. The vortex was detected over the entire 24 h time period. It had a radius of 2.5 Mm, was cospatial with a downflow, and consisted of 3 recurrent vortices with a lifetime of $\sim 7~\text{h}$ each. The evolution of magnetic elements detected in the core of the vortices were strongly affected by the evolution of these vortices, more specifically, they were concentrated and evacuated when they were caught by the vortices and weakened and fragmented after the latter disappeared.

Giagkiozis et al. (2018) applied a fully automated vortex identification method based on $\varGamma $-functions (see Sect. 4.3) on velocities obtained with LCT from photospheric CRISP/SST Fe i continuum data. They detected intergranular intensity vortices (see Fig. 1) with lifetimes of $\sim 17~\text{s}$, much shorter compared to previously observed magnetic BP swirls. They suggested that, at any time, there were $1.48 \times 10^{6}$ small-scale vortices covering $\sim 2.8\%$ of the solar surface.

### Chromospheric Observations

Chromospheric detections of vortex flows are more sparse compared to photospheric ones and rely heavily on the high-quality and high temporal and spatial resolution observations of the modern era. All chromospheric detections up to now relied on observations with the Crisp Imaging Spectropolarimeter (CRISP) of the SST, which can provide very high-resolution observations due to the adaptive optics of the telescope and the application of image restoration techniques.

**Fig. 9:**
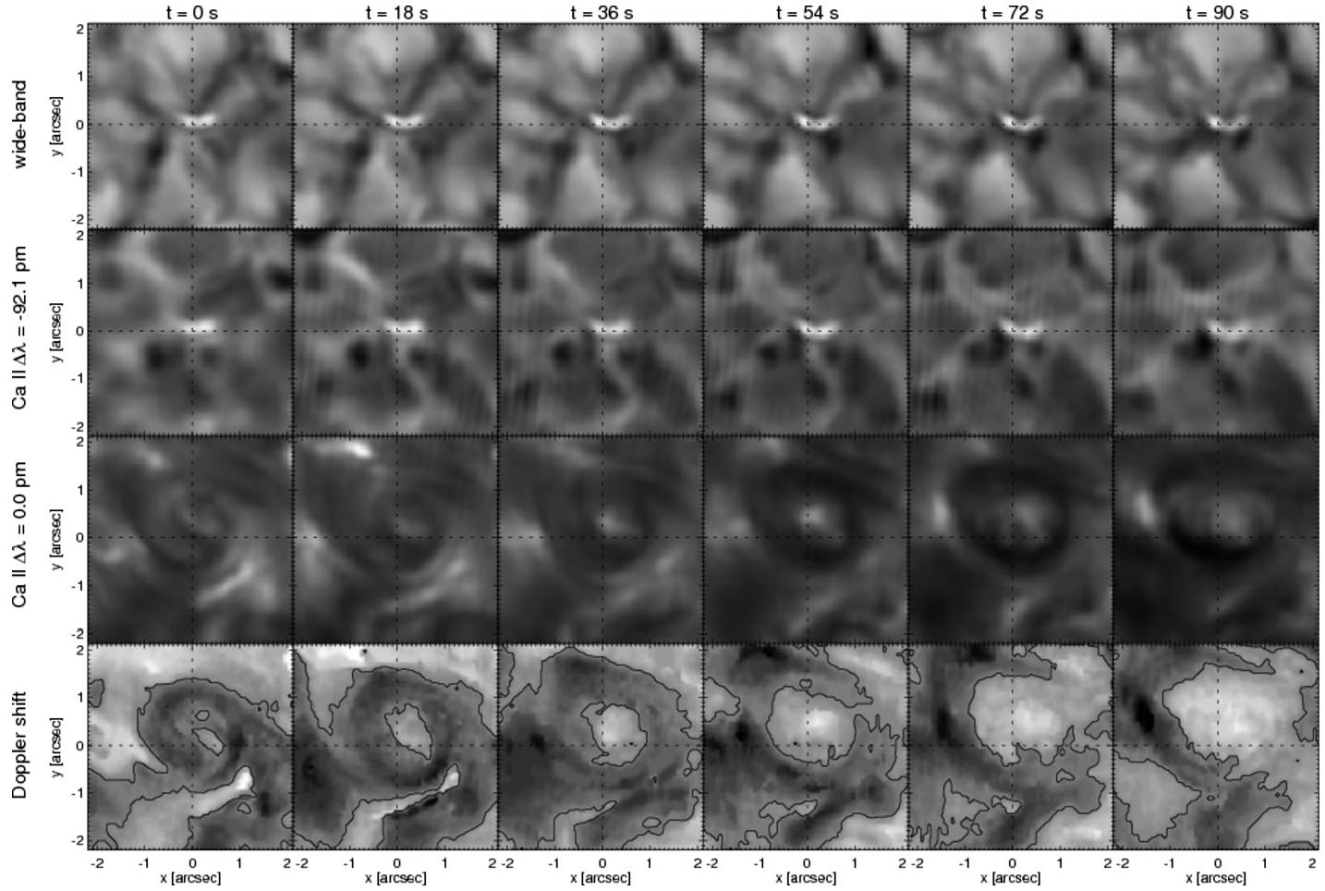
Snapshots from the temporal evolution (from left to right) of a chromospheric swirl seen in a wide-band filter centered on 854.2 nm, in narrow band filters of the Ca ii 854.2 nm line wing and the Ca ii line core, and of the Doppler shift (top to bottom rows, respectively). Black contours in the Doppler shift images indicate a zero Doppler shift value with grey scale shifts ranging from $-5.5~\text{km}\,\text{s}^{-1}$ (upflows) to $+5.5~\text{km}\,\text{s}^{-1}$ (downflows). Credit: Wedemeyer-Böhm and Rouppe van der Voort (2009), reproduced with permission ©ESO

Wedemeyer-Böhm and Rouppe van der Voort (2009) were the first to reveal the presence of conspicuous chromospheric swirling motions, which they termed chromospheric swirls. They studied two time series of spectral scans through the Ca ii IR line at 854.2 nm of a coronal hole region located close to the solar disk centre (see Fig. 9). The observations were acquired with CRISP. Chromospheric swirls appeared in line core maps as dark rotating patches, which together form arcs, spiral arms, rings or ring fragments. These rotating structures had typical diameters of $2''$, while finer sub-structure on scales down to $\sim 0.2''$, i.e., close to the telescope’s resolution limit, was observed. The chromospheric material was found to follow upward LOS motions, with Doppler velocities between 2 and $4~\text{km}\,\text{s}^{-1}$, but sometimes even stronger upflows up to $7~\text{km}\,\text{s}^{-1}$ were also observed. Since many of these chromospheric swirls were associated with groups of (photospheric) BPs moving with respect to each other and observed in wideband images that map the lower photosphere, it was suggested that the proper motions and interactions between BPs can produce twisted and braided magnetic configurations that could support chromospheric swirls (although no clear connection was found). They provided an estimate of the frequency of swirl event occurrence of $\sim 1.24\times 10^{-4}~\text{Mm}^{-2}\,\text{min}^{-1}$. In the same study, it was also stated that the low abundance of fibrils found in coronal holes favour the detection of swirling structures, which would be otherwise inhibited by the presence of these inclined structures that form the magnetic canopy (Kontogiannis et al. 2010, 2014).

**Fig. 10:**
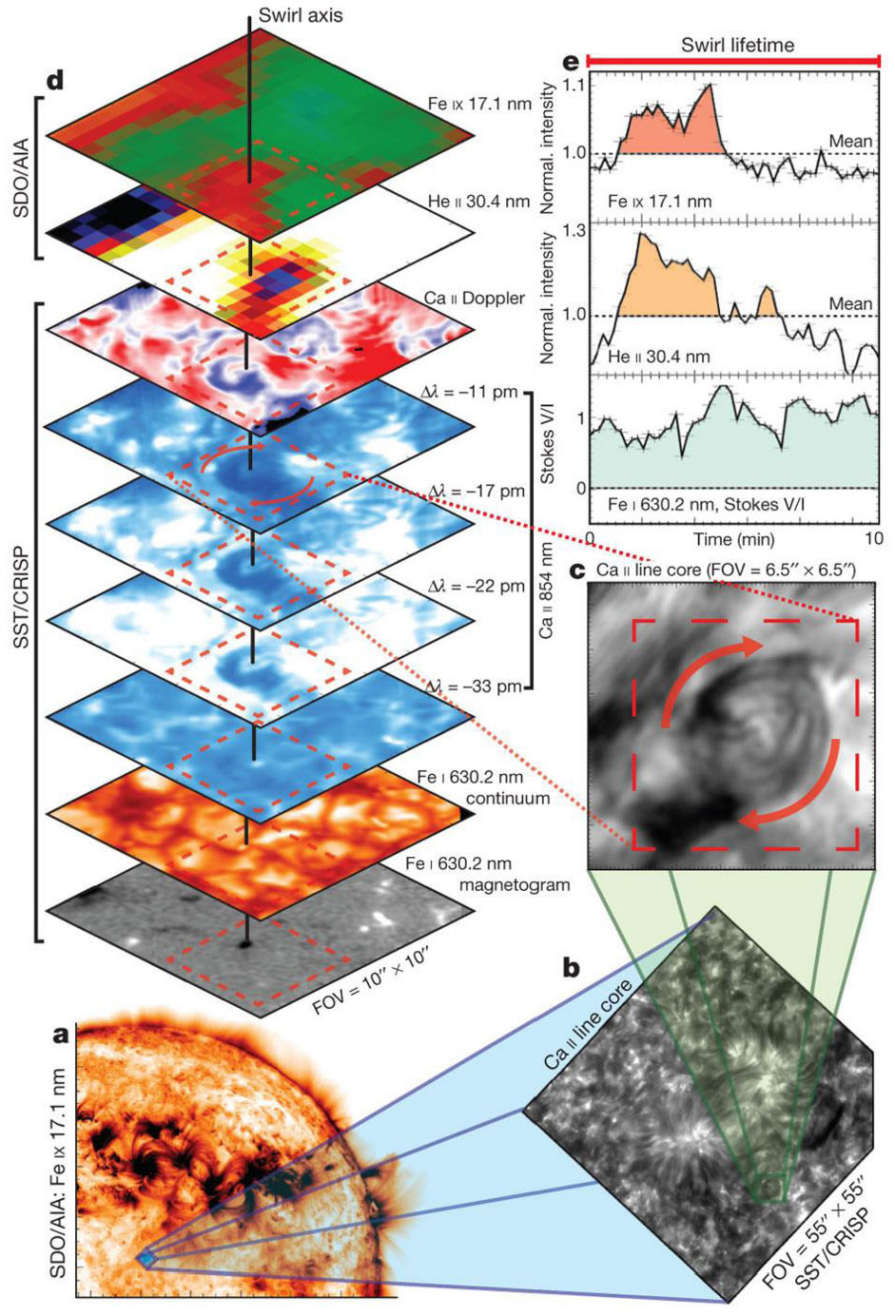
Co-aligned observations from CRISP/SST and AIA/SDO showing a chromospheric swirl (panel **c**) and its response up to the low corona (panels **d**). From Wedemeyer-Böhm et al. (2012)

The magnetic nature of the chromospheric swirls was further supported and explored by Wedemeyer-Böhm et al. (2012). They used a 55 minute-long time series of spectral imaging in 47 positions across the Ca ii IR line, and a cadence equal to 14 s. Their observations also included Fe i 630.2 nm photospheric magnetograms. Within this dataset they detected 14 swirls, with lifetimes around 12.7 min. Simultaneous SDO observations in 304, 171, 193, and 211 Å revealed a response in the transition region and the corona, in the form of brightenings associated with the fragmented rings seen in the chromosphere. All swirls had detectable associated emission in the 304 Å, but not all of them appeared in the hotter channels. This finding pointed to swirls being parts of coherent magnetic structures reaching various heights (Fig. 10). They further used CO^5^BOLD and BIFROST simulations to corroborate their findings in numerical experiments. They concluded that the magnetic field that is concentrated in the intergranular lanes, under the constant action of the granular velocity field and the photospheric oscillations, creates a coherent, roughly vertical, structure, which permeates the solar atmosphere and can channel plasma towards the upper layers through spiral trajectories. Gas parcels are moving upwards and downwards with a net upward mass flux propelled by the centrifugal force, while torsional Alfvén waves carry substantial amounts of Poynting flux to the lower corona. This finding established vortices and swirls as potential contributors to coronal heating.

The action of photospheric motions to chromospheric structures was studied by Morton et al. (2013). They used time series of G-band and $\text{H}\alpha $-core narrowband images, acquired with the Rapid Oscillations in the Solar Atmosphere (ROSA, for details see Jess et al. 2010), installed at the Dunn Solar Telescope, in Sacramento Peak (USA). LCT was applied to both G-band images to infer the photospheric flow field and to unsharp-masked and spatially filtered $\text{H}\alpha $ filtergrams to infer chromospheric motions. At the photospheric level, signatures of vortex flows were identified in the velocity vector plots. Their chromospheric counterpart exhibited a quasi-periodic torsional motion as a response to photospheric vortical motions. The period of this motion was between 120 and 180 s and the horizontal velocity amplitude exhibited a maximum of $7~\text{km}\,\text{s}^{-1}$. This torsional motion was found to drive transverse waves, which were supported by the nearby chromospheric fibrils.

Liu et al. (2019a) studied time series of Ca ii H of a quiet region close to disk center, taken with SOT onboard Hinode. Using an automated swirl detection method based on $\varGamma $-functions (see Sect. 4.3), they reported average values of $\sim 21~\text{s}$ for the swirl lifetime, $\sim 290~\text{km}$ for the effective radius, $1.8~\text{km}\,\text{s}^{-1}$ for the rotating speed, and an estimated population of swirls in the chromosphere higher than $3.7\times 10^{5}$. A correlation analysis with photospheric swirls seen in co-temporal G-band time series, and similar correlation analyses between swirls seen in other spectral lines of a co-temporal SST dataset, yielded small time lags. In combination with supporting MHD simulations, they interpreted this as evidence for Alfvén pulses, ubiquitously excited at the photosphere and travelling upwards, reaching the chromosphere. We note, however, that the used chromospheric proxy relates to the upper photosphere/lower chromosphere and that the height difference between Ca ii H and G-band is small, as the filter bandwidths are large (3 and 4 Å, respectively) and reverse granulation (Rutten et al. 2004) is clearly visible in Ca ii H.

The first detection of chromospheric swirls in the $\text{H}\alpha $ line was reported by Park et al. (2016), who examined coordinated observations of a quiet Sun region taken by SST/CRISP and by the Interface Region Imaging Spectrograph (IRIS, De Pontieu et al. 2014). They found a small scale swirl (up to 1 Mm on the image plane), which was simultaneously observed in the $\text{H}\alpha $, Ca ii 8542 Å and Mg ii k lines. The swirl consisted of spiral arms and exhibited expanding and swirling motions with apparent speeds up to 5 and $13~\text{km}\,\text{s}^{-1}$, respectively. Spectral analysis of the Mg ii k and the Mg ii subordinate lines, which map the upper chromosphere and transition region, showed strong upflows up to $8~\text{km}\,\text{s}^{-1}$ related to the spiral arms. During the upflows a temperature increase and heating signature were also derived from the spectral analysis of the same lines.

**Fig. 11:**
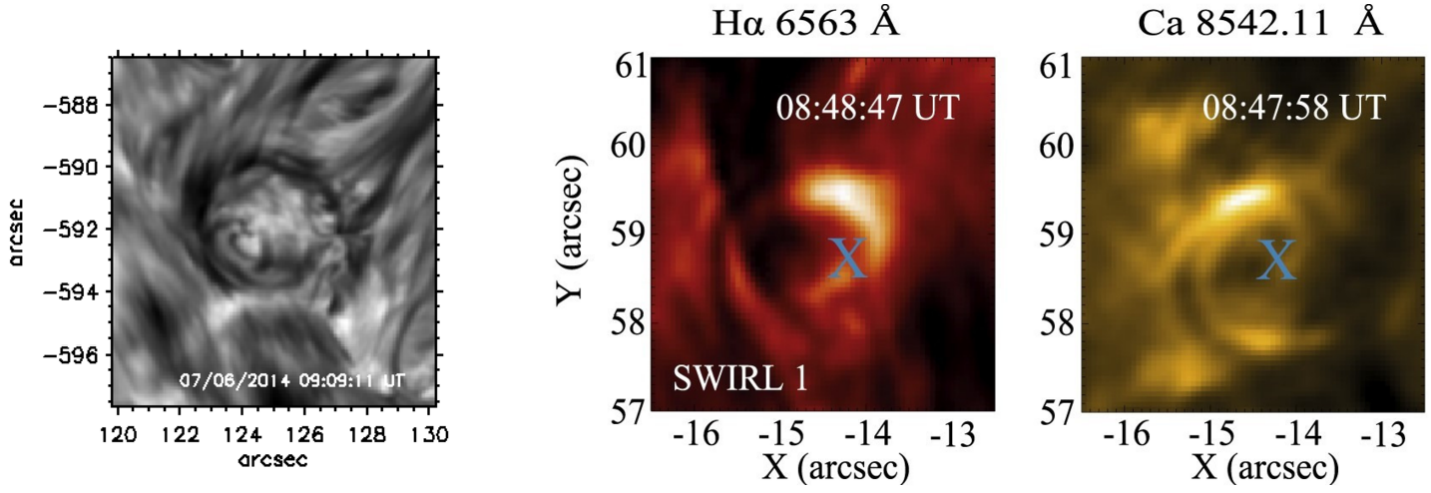
Left panel: Snapshot of the persistent small-scale tornado observed in $\text{H}\alpha $ line centre. Credit: Tziotziou et al. (2018), reproduced with permission ©ESO. Right panel: Snapshots of a swirl observed in $\text{H}\alpha $ and Ca ii 8542 Å. From Shetye et al. (2019)

Using the same dataset, Tziotziou et al. (2018) detected a persistent small-scale tornado in a quiet Sun region (see Fig. 11, left panel). The small-scale event had a duration that exceeded 1.7 h and a radius of $\sim 3''$. It was located in a supergranular cell and it had a clear interaction with neighboring chromospheric fibrilar structures. The vortex flow appeared very clearly in the $\text{H}\alpha $ line centre and close to line centre wavelengths, but it was barely seen in the $\text{H}\alpha $ wings. In the Ca ii 8542 Å line, instead, only a small dark patch is seen in the line centre and close to line centre wavelengths, which was interpreted as due to the different formation mechanisms of the two lines. Within the vortex flow a significant substructure was revealed. It manifested in the form of at least four small swirls, which were not always visible, but they had an intermittent character appearing and disappearing around the same location during the entire time series. Their morphological patterns varied with time and had the form of rings (either whole or fragments of them), spirals and spiral arms/arcs. These patterns were mostly associated with mean upwardly directed Doppler velocities of $\sim 3~\text{km}\,\text{s}^{-1}$ and sometimes reaching up to $8~\text{km}\,\text{s}^{-1}$. Intensity time-slice images revealed a mean velocity of $\sim 3~\text{km}\,\text{s}^{-1}$ for the radial expansion of the spiral flow. A swaying motion was also inferred. Detailed analysis showed that the dynamics of the observed vortex structure can be interpreted by the dynamics of a rigidly or quasi-rigidly, clockwise-rotating logarithmic spiral with a complementary swaying motion. Further analysis of the oscillatory behaviour in the vortex area showed a swaying period between 200–220 s and rotation periods varying with chromospheric height that are $\sim 215~\text{s}$ at the formation level of Ca ii IR and $\sim 270~\text{s}$ higher up at the formation height of the $\text{H}\alpha $ line centre (Tziotziou et al. 2019, more details in Sect. 8.2.2). No magnetic BPs were observed in the $\text{H}\alpha $ and Ca ii 8542 Å wings related to the vortex area. HMI/SDO LOS magnetograms showed very weak and noisy magnetic fields in the area of the vortex of strength within the $\sim 20\text{--}30$ Gauss HMI uncertainty. These indicated at certain time intervals the presence of weak magnetic flux concentrations that appeared systematically at specific locations within the vortex area. Based on this finding, as well as on the oscillatory behaviour it was conjectured that this structure was magnetically supported. Comparison of the chromospheric observations with simultaneous AIA/SDO UV and EUV observations showed that the vortex area appeared as an intensity decrease in the lower temperature AIA channels.

Further evidence on the magnetic nature of chromospheric swirls was provided by Shetye et al. (2019). They used simultaneous spectral imaging in $\text{H}\alpha $ and Ca ii IR (see Fig. 11, right panel), along with spectropolarimetry in one wavelength position of the photospheric Fe i 6302 Å line, taken with the CRISP instrument at the SST. The thirteen swirls they detected visually were all associated with photospheric magnetic field concentrations. Five of them were traced down to a single photospheric magnetic structure, while the rest were linked to two or more. However, no noticeable differences were seen between the two categories. The swirls had a circular pattern with a $\sim 2~\text{Mm}$ average diameter and a lifetime of $\sim 9$ to 10 min. They found radially expanding motions with speeds of $\sim 10$ to $20~\text{km}\,\text{s}^{-1}$, i.e., close to or higher than the local sound speed, as well as rotational motions with periods in the range from 100 to 150 s. Their observations validate the picture drawn by Wedemeyer-Böhm et al. (2012) and are in line with previous $\text{H}\alpha $ observations of swirls. Additionally, they found periodic intensity variations with a period of 180 s, that is consistent with the 3 min acoustic oscillations in the chromosphere. They also reported a decrease of the local oscillation periods from 180 s to 150 s which, as they suggested, could be attributed to a change in the dimensions of the acoustic cavity, an increase in temperature, or even additional magnetic effects (more details in Sect. 8). These findings suggest that in the presence of chromospheric swirls, the oscillatory properties of the chromosphere are modulated, but there were no indications of wave excitation due to the swirls.

Murabito et al. (2020) observed two structures with clockwise rotation in the Fe i 6173 Å and Ca ii 8542 Å lines, taken with the IBIS instrument. The two structures were associated with clear circular polarization signals both at the photosphere and at the chromosphere, the latter signifying the first swirl-associated polarization signal detected at a layer higher than the photosphere. Although no further analysis of the Stokes profiles was performed, the distribution of the polarization was interpreted as representing a non-potential, connected dipole, wherein the one polarity wrapped itself around the other, as a result of the swirling motion. A numerical experiment with the Lare3d MHD code was also performed to support this scenario.

Recently, Dakanalis et al. (2022) detected chromospheric swirls in $\text{H}\alpha $ CRISP/SST observations, using the automated morphological method of Dakanalis et al. (2021), and provided a detailed statistical analysis of various properties, including the derivation of their lifetime with a survival analysis based on the Kaplan-Meier estimator (Kaplan and Meier 1958) combined with a parametric model. They found considerably higher numbers of swirls than previous reports did: a mean number of $146\pm 9$ swirls at any given time, with a mean surface density of $\sim 0.08$ swirls per $\text{Mm}^{2}$ and an occurrence rate of $\sim 10^{-2}$ swirls per $\text{Mm}^{2}\,\text{min}^{-1}$. Their radii range between 0.5–2.5 Mm with a mean value of $1.3\pm 0.3~\text{Mm}$ and their lifetimes range between 1.5–33.7 min (the upper value corresponding to the duration of the observations) with a mean lifetime of $10.3\pm 0.6~\text{min}$. Their work suggests the ubiquitous presence of swirls in the solar chromosphere and that automated detection methods combined with higher-cadence/higher-resolution observations can lead to the detection of swirls of larger population, smaller-scale, and shorter-lifetime.

Table 5 summarizes the properties of small-scale vortices and chromospheric swirls derived from the photospheric and chromospheric observations presented and discussed in this and the previous subsection.

**Table 5:** Observations and properties of photospheric vortex flows and chromospheric swirls. Negative (positive) Doppler velocities correspond to downflows (upflows)

Reference	Duration	Diameter [Mm]	Average flow speed [km s^−1^]	Doppler velocity [km s^−1^]	Lifetime	Vorticity [s^−1^]	Space-time density [Mm^−2^ min^−1^]	Total number at any given time [Mm^−2^]
Photospheric observations of vortex flows – Properties								
Brandt et al. (1988)^a^	1.5 h	5.0	0.67		>1.5 h	1.4 × 10^−3^		
Bonet et al. (2008)^b^	33 min	≲1	1 to 4		5.1 ± 2.1 min		1.8 × 10^−3^	9 × 10^−3^
Attie et al. (2009)^c^	1 h & 4 h	15.5 & 21	0.2–0.3		>1 h			
Bonet et al. (2010)^d^	31.6 min				7.9 ± 3.2 min	<6 × 10^−3^	3.1 × 10^−3^	2.4 × 10^−2^
Balmaceda et al. (2010)^e^	20 & 30 min	1.8	1.5–3	-1.3–(-2)	>20 min			
Vargas Domínguez et al. (2011)^b^	2 × 20 min	0.5	0.48	-0.53	10–20 min	2.1 × 10^−3^	1.4 × 10^−3^	2.8 × 10^−2^
							1.6 × 10^−3^	3.1 × 10^−2^
Requerey et al. (2017)^d^	45.9 min	0.48	0.23			1.5 × 10^−3^		2.4 × 10^−2^
Requerey et al. (2018)^f^	24 h	5		-0.2–(-0.45)	3 consecutive vortices, 7 h each	1.4 × 10^−4^		
Giagkiozis et al. (2018)^g^	57 min	0.57	0.42		0.29 min		0.84	2.4 × 10^−1^
Steiner et al. (2010)^d^^,^^q^	53.9 min		1–4	0.8–1.4	1.5–6.5 min		2.8 × 10^−4^	
Chromospheric observations of swirls – Properties								
Wedemeyer-Böhm and Rouppe van der Voort (2009)^h^	33 min, 53 min	1.5	1.5–2	2–7			1.24 × 10^−4^	
Wedemeyer-Böhm et al. (2012)^i^	55 min	2.9 ± 1		4	12.7 ± 4 min			2 × 10^−3^
Morton et al. (2013)^j^	70 min	3	1.8 ± 1.4					
Park et al. (2016)^k^	49 min	0.6 & 1.4	13	<8				
Tziotziou et al. (2018)^l^	1.7 h	4.4		3–8	>1.7 h			
Liu et al. (2019a)^m^^,^^r^	151.8 min	0.58	1.8 ± 0.7		0.35 min			6.1 × 10^−2^
Shetye et al. (2019)^n^	58 min	2		2–6	9–10 min			
Murabito et al. (2020)^o^	45 min	2.2	<2		>45 min			
Dakanalis et al. (2022)^p^	33.7 min	2.6 ± 0.6			10.3 ± 0.6 min		∼10^−2^	∼8 × 10^−2^

^a^SST filtergrams at 4696 Å

^b^SST filtergrams at G-band

^c^Hinode/SOT filtergrams at blue continuum and G-band

^d^SUNRISE I/IMaX Fe i 5250 Å 4 Stokes parameters

^e^SST several lines, Hinode/BFI/NFI/SP

^f^Hinode/NFI Na i D_1_ Stokes I and V

^g^SST CRISP, Fe i continuum

^h^SST CRISP, Ca ii IR

^i^SST CRISP, Ca ii IR, Fe i, SDO AIA

^j^ROSA filtergrams in G-band and H*α*

^k^SST CRISP, Ca ii IR, H*α*, IRIS Mg ii

^l^SST CRISP, Ca ii IR, H*α*, SDO AIA and HMI

^m^Hinode/SOT filtergrams at G-band Ca ii H – SST CRISP, Fe i wide-band, Ca ii IR core, H*α* core

^n^SST CRISP, Ca ii IR, H*α*, Fe i

^o^IBIS, Ca iiIR, Fe i

^p^SST CRISP, H*α*

^q^Properties of horizontal vortex tubes at granular boundaries

^r^Observations mapping the upper photosphere/low chromosphere (see text)

### Transition Region and Coronal Observations

Accounting for all the observed properties of vortices, it is clear that vortex flows in the solar atmosphere have a significant vertical extent and can reach up to the corona. All detected swirls shown in Wedemeyer-Böhm et al. (2012) exhibited emission in the 304 Å channel of AIA, commonly associated with the transition region (TR), while for some of them, emission was seen also in the 171, 193, and 211 Å channels, corresponding to heights beyond the TR. These observations indicated that swirls are not merely chromospheric or photospheric features but can reach up to various heights, possibly contributing to the atmospheric mass/energy balance. In line with these findings, Tziotziou et al. (2018) observed a persistent quiet Sun tornado that was also clearly associated with emission seen in the AIA channels.

However, our understanding of the transition region and corona of chromospheric vortices is hindered by the limitations of EUV observations—chromospheric swirls are fine structures, below or, at best, marginally at the resolution capacity of current UV/EUV imaging facilities, which explains the limited number of pertinent studies. On the contrary, recent observations with SDO and IRIS, as well as older ones from instruments on Hinode, STEREO, and SUMER have focused on larger structures such as rotating jets and macrospicules, solar tornadoes, and “cyclones” (see Sect. 2 and Tables 1–4, for definitions and nomenclature).

One of the earliest mentions of solar tornadoes was based on a comprehensive survey of EUV spectroscopic observations by Pike and Mason (1998), who attributed the spectroscopic properties of macrospicules, observed with the Coronal Dynamics Spectrometer, to rotating plasma, accelerating with height. The helical motion of a macrospicule was also seen by Kamio et al. (2010) in simultaneous observations taken with Hinode/EIS and XRT, STEREO/EUVI, and SOHO/SUMER. These structures were found to rotate in the form of a vertical cylinder with no pronounced funnel-shaped expansion, contrary to what is seen in chromospheric swirls. Furthermore, given the relatively low spatial resolution of those instruments and the large spatial extent of macrospicules, the correspondence of these phenomena to the finer chromospheric swirls seen routinely in the quiet Sun is not obvious.

As already mentioned, the term “tornado” is often used to describe persistent large-scale structures observed in prominence legs, which resemble tornadoes on Earth. This resemblance is, however, only morphological, since solar tornadoes typically involve twisted magnetic fields and their physics greatly differs from that of terrestrial tornadoes. Additionally, it is not fully clear whether the apparent rotation of solar tornadoes represents actual rotating mass or the visual effect in the plane of sky of counterstreaming flows. We note, for historical reasons, that “tornado prominences” were first introduced and studied by Pettit (1941, 1943, 1946, 1950).

Okamoto et al. (2010) studied the rise of a twisted column of cool plasma, which led to the formation of a prominence and a coronal cavity. The structure was seen at the limb, in Ca ii H (Hinode/SOT), in EUV (STEREO/EUVI), and the soft X-rays (Hinode/XRT). In the SDO-era, Li et al. (2012) combined EUV imaging with ground based $\text{H}\alpha $ context imaging to find plasma movement along unstable, rising and expanding helical structures in the cavity of a prominence. Su et al. (2012) used observations from SDO and STEREO to study a filament/prominence from two different vantage points. They associated solar tornadoes with filament barbs and suggested that vortex flows at the legs of prominences, driven by photospheric motions, are playing a role in the filament formation. However, they cautioned that several aspects of this interpretation and association needed further clarification.

Rotating motions of plasma at the feet of quiescent prominence were also detected by Orozco Suárez et al. (2012) in the line of He i 10830 Å observed with the GREGOR Infrared Spectropolarimeter (GRIS, Collados et al. 2012). These persistently rotative motions had a LOS Doppler velocity equal to $6~\text{km}\,\text{s}^{-1}$, much lower than the $20~\text{km}\,\text{s}^{-1}$ derived from EUV imaging. The velocity pattern of a solar tornado in EUV was further studied by Levens et al. (2015). They used spectra from Hinode/EIS to deduce Doppler shifts, densities, non-thermal widths, and emission measure distributions. The plasma in the tornado exhibited rotational motion, lower density than the surroundings, and perhaps turbulence among other possible mechanisms, which contributed to increased spectral line widths. The DEM analysis showed that the tornado contained contributions from cooler plasma, compared to the main body of the prominence. Spectropolarimetry with THEMIS, coordinated with observations by SDO, IRIS, and Hinode was further employed by Levens et al. (2016b), who found no evidence of rotation of a tornado in the Mg ii k line. Its horizontal magnetic field was parallel to the limb and it exhibited oscillations in EUV. The same set of observations was later used to calculate correlations between different parameters of the tornado (Levens et al. 2017). A study of spectropolarimetric observations in the He i line of a large sample of tornadoes showed that these phenomena were not necessarily compatible with helical magnetic fields (Levens et al. 2016a).

Wedemeyer et al. (2013b) aimed to put the giant tornadoes into the context of a spectrum of vortical phenomena in the Sun, by studying a sample of 201 tornadoes in the 171 Å channel of AIA. Their results strengthen the association between these structures and filaments, indicating that tornadoes may act as sources and sinks of cool plasma, also producing twist and potentially acting as triggers for eruptions. The relation between tornadoes and solar eruptions has been supported by further EUV observations. Chen et al. (2017) used SDO time-series of images to show the triggering of a solar tornado, exhibiting blobs and spiral arms, after the interaction between jets and filaments, while Panesar et al. (2013) observed a series of three flares with SDO and STEREO, which produced a stable tornado nearby.

However, the controversy about giant tornadoes is not yet resolved. Panasenco et al. (2014) examined observations from SDO, along with STEREO and ground-based context imaging in $\text{H}\alpha $ from the Big Bear Solar Observatory (BBSO), to support that the observed rotation of tornadoes is an optical effect caused by the projection of counterstreaming motions and oscillations in prominence barbs. Magnetohydrodynamic oscillations were also suggested to play a role by Martínez González et al. (2016), who analyzed spectropolarimetric observations in the He i 10830 line. The derived magnetic field of the tornadoes was found to have a helical structure, but it was concluded that the rotation of the structure could be, at best, intermittent and not continuous. In context imaging in EUV, provided by SDO, and $\text{H}\alpha $ observations taken with the Multi-channel Subtractive Double Pass (MSDP) instrument in the Meudon Solar Tower, Schmieder et al. (2017a) also detected no signs of rotation and interpreted Doppler shifts as the result of oscillations. On the other hand, the Doppler shifts of Mg ii k (2796 Å) and Si i (1393 Å) lines (IRIS) and SDO observations of a tornado in a prominence leg by Yang et al. (2018) supported the scenario of rotating motions of cool plasma.

We are closing this account of transition region and coronal observations of vortex-like structures with a reference to intermediate scale structures called “cyclones” or, also, “solar tornadoes”, which are seen in on-disk EUV observations. These, often loop-like structures can be formed by persistent rotation of magnetic concentrations in the quiet-Sun (Zhang and Liu 2011; Yu et al. 2014). Although these structures exhibit some morphological similarities with swirls seen in the chromosphere (albeit considerably larger), their properties are outside the scope of this review.

## Numerical Simulations of Vortex Motions

The turbulent nature of solar convection causes multi-scale interaction of subsurface layers with the photosphere and the chromosphere. Ubiquitous small-scale vortices are thus generated by the turbulent flow beneath the visible surface, mainly gathered in the intergranular lanes and the borders of granules. These can further capture and amplify the local magnetic field and reach into chromospheric and higher layers, causing several interesting dynamical phenomena related to vortical motions.

Several advanced three-dimensional numerical simulations have been carried out specifically for the purpose of studying vortical motions in the solar atmosphere. These simulations are now realistic enough to be directly compared to high-resolution observations from the ground and from space. This is achieved by computing synthetic diagnostics from the models, which then can be compared with high-resolution observations. Simulations also serve for the planing of observational campaigns and for observational predictions. The similarities and discrepancies between observations and simulations help us to identify the essential physical mechanisms of mass and energy transfer in the solar atmosphere. In particular, numerical simulations help us to understand the origin, role, and significance of vortical motions in the solar atmosphere.

In this section we discuss simulations that self-consistently produce vortex motions within a (magneto)hydrodynamically simulated solar atmosphere. There exist, however, several simulations where the atmosphere and the magnetic topology of the structure (e.g. magnetic flux tube) are magneto-hydrostatically predefined within the simulation box and then evolved in time. Such simulations, for example by Fedun et al. (2011a,c), Shelyag et al. (2008, 2016), Chmielewski et al. (2014), Murawski et al. (2015, 2018), that mainly simulate the interaction of an arbitrary perturbation with a (magneto)hydrostatic equilibrium background, have been widely used for the study of excitation and propagation of different wave modes within vortex tubes. These kind of simulations are further detailed and discussed in Sect. 8.2.2.

Moreover, in this section we also discuss vorticity in jets and the initiation of quasi-periodic flow eruptions in vortex flux tubes as revealed from simulations.

### Basic Aspects of Numerical Simulations of Vortex Motions

As we have already seen in Sect. 6, there exist different types of vortices in the solar atmosphere. According to Wedemeyer and Steiner (2014) atmospheric vortex flows (AVF, also called “magnetic tornadoes”; Wedemeyer-Böhm et al. 2012) occur as a consequence of intergranular vortex flows (IVF) in the deep photosphere below. Simulations of photospheric vortices need the inclusion of the top layers of the convection zone and the photosphere and do not require magnetic fields. Pure hydrodynamical treatment in three spatial dimension is sufficient as these vortices form as a direct consequence of the angular momentum conservation and are thus an integral part of stellar surface convection. Simulations of magnetic tornadoes, however, require in addition a sufficiently large layer on top and magnetohydrodynamical treatment. The derivation of chromospheric synthetic diagnostics for comparison with observed chromospheric swirls as the one shown in Fig. 10c must include chromospheric layers beyond 1000 km above the optical continuum depth $\tau _{500} = 1$. Simulations with insufficient vertical height extent will thus not exhibit such swirls or be strongly hampered by the top boundary condition.

A detailed treatment of radiative transfer is desirable but not needed in order to produce vortex flows in simulations. Even simulations with frequency-independent (grey) radiative transfer produce vortex flows as a natural consequence of the simulation’s (magneto)hydrodynamics. The spatial resolution of the numerical grid, however, is crucial. It needs to be high enough to resolve the spatial scales on which vortices (in the photosphere) form. An overview over different published simulations that were used for the purpose of studying vortex flows is given in Table 6. All of these simulations include the top layers of the convection zone but with different depths for the lower boundary ranging from only $-800~\text{km}$ to $-5200~\text{km}$ below the bottom of the photosphere (defined as the height where the optical depth $\tau _{500} = 1$). For the magnetic tornadoes, it turns out that the generation of small-scale vortex flows in the deep photosphere is a very localized process that can take place in a comparatively shallow layer. Consequently, even simulations that extend only a few hundred kilometers into the convection zone do exhibit small-scale photospheric vortex flows. On the other hand, the generation of chromospheric swirls requires the top boundary to be located high enough and it requires the presence of photospheric magnetic flux concentrations. The upper boundaries of the magneto-hydrodynamical simulations listed in Table 6 vary from 600 km to 14 100 km. Chromospheric swirls can only develop in a simulation that includes a chromospheric layer with heights well above 1000 km. The upper boundary can have significant influence on the behaviour of the magnetic field in the chromosphere and thus on the dynamics of chromospheric swirls and should thus be placed well above the height range of interest. Therefore, some simulations listed in Table 6 can not fully develop chromospheric swirls but still do exhibit photospheric vortex flows.

**Table 6:** List of hydrodynamic and magnetohydrodynamic convective simulations, for which analyses of vortex motions in the simulated solar atmosphere was performed. The table highlights important differences between the simulation parameters. The various codes used (in alphabetical order) have references as follows: ANTARES (Muthsam et al. 2010), Bifrost (Gudiksen et al. 2011), CO^5^BOLD (Freytag et al. 2012), MANCHA3D (Khomenko and Collados 2006; Felipe et al. 2010; González-Morales et al. 2018), METEOSOL (Amari et al. 1999), MURaM (Vögler et al. 2005), SolarBox (Jacoutot et al. 2008). Updated table adapted from Wedemeyer and Steiner (2014)

Reference	MHD code	Initial magnetic field $|B_{0}|$	Height of lower boundary	Height of upper boundary
Simulations with no or weak magnetic field				
Muthsam et al. (2010)	ANTARES	–	$-450~\text{km}$	2313 km
Steiner et al. (2010)	CO^5^BOLD	10 G^a^	$-1400~\text{km}$	1400 km
Moll et al. (2011b)	MURaM	dynamo^b^	$-890~\text{km}$	510 km
Kitiashvili et al. (2011)	SolarBox	–	$-5000~\text{km}$	500 km
Freytag (2013)	CO^5^BOLD	–	$-1700~\text{km}$	1100 km
Moll et al. (2012)	MURaM	–	$-900~\text{km}$	800 km
Shelyag et al. (2011b)	MURaM	–	$-800~\text{km}$	600 km
Steiner and Rezaei (2012)	CO^5^BOLD	–	$-1400~\text{km}$	1400 km
Kitiashvili et al. (2013)	SolarBox	–	$-5200~\text{km}$	1000 km
Amari et al. (2015)	METEOSOL	dynamo^b^	$-1500~\text{km}$	15000 km
Calvo et al. (2016)	CO^5^BOLD	–	$-1400~\text{km}$	1400 km
Magnetohydrodynamic simulations				
Vögler (2004)	MURaM	200 G	$-800~\text{km}$	600 km
Carlsson et al. (2010)	Bifrost	40 G	$-1400~\text{km}$	14100 km
Shelyag et al. (2011b, 2013)	MURaM	200 G	$-800~\text{km}$	600 km
Steiner and Rezaei (2012)	CO^5^BOLD	50 G	$-1400~\text{km}$	1400 km
Moll et al. (2012)	MURaM	200 G	$-900~\text{km}$	800 km
Wedemeyer-Böhm et al. (2012)	CO^5^BOLD	50 G	$-2400~\text{km}$	2000 km
Kitiashvili et al. (2013)	SolarBox	10 G	$-5200~\text{km}$	1000 km
Kato and Wedemeyer (2017)	CO^5^BOLD	50 G	$-2400~\text{km}$	2000 km
Khomenko et al. (2018, 2021)	MANCHA3D	dynamo^c^	$-950~\text{km}$	1400 km
	MANCHA3D	10 G	$-950~\text{km}$	1400 km
Yadav et al. (2020, 2021)	MURaM	200 G	$-1500~\text{km}$	2500 km
Silva et al. (2020, 2021)	MURaM	200 G	$-1000~\text{km}$	600 km
Battaglia et al. (2021)	CO^5^BOLD	50 G	$-1300~\text{km}$	1500 km
Aljohani et al. (2022)	MURaM	200 G	$-1000~\text{km}$	600 km
Rajaguru et al. (2023)	CO^5^BOLD	50 G	$-1400~\text{km}$	1400 km

^a^Upflows carry horizontal magnetic field of 20 G strength into the simulation domain across the bottom boundary resulting in a mean vertical field of $\langle |B_{z}|\rangle \approx 10~\text{G}$ at the visible surface

^b^The magnetic field is self-consistently generated by a turbulent small-scale dynamo in the convective layers resulting in a mean vertical field of $\langle |B_{z}|\rangle \approx 25~\text{G}$ at the visible surface

^c^Same as in ^b^ but with $\langle |B|\rangle \approx 100~\text{G}$ at the visible surface

### Vortex Motions in Weak Magnetic Field or Field-Free Simulations

There exist a number of non-magnetic or weakly magnetic simulations (see also Table 6) that produce true non-magnetic vortices with true vortex flows. In these cases, the magnetic field is not strong enough to affect the flow significantly. Such true vortex flows often lead to the partial evacuation of the vortex tube—similarly to magnetic flux tubes—and ultimately to the formation of non-magnetic bright points (nMBPs). Observations of, e.g., Berger and Title (2001), or Langhans et al. (2002) suggest that nMBPs do exist and are similar in appearance to the known magnetic bright points but it is not clear if these specific examples were formed by vortices. Several magnetic field-free or weak field simulations, including simulations resulting in nMBPs, are discussed below.

Nordlund (1985) noticed from numerical simulations “a circular motion around the center of the downdraft, and the circular velocity is amplified as the downdraft narrows (‘bathtub’ or ‘inverted tornado’)” and he pointed to the centripetal force of that vortical flow. Like Brandt et al. (1988), he conjectured consequences of such ‘inverted tornadoes’ for the outer layers of the atmosphere and “that any vertical magnetic field lines in the surrounding photosphere must be carried towards, and ‘sucked into’, these downdrafts”. Stein and Nordlund (1998) pointed to the baroclinic term as the primary source of vorticity in this case. It appears whenever the gradients of gas pressure and density are not parallel to each other (see fourth term on the r.h.s. of Eq. (7)). It is particularly large at the edges of granules and the generated vorticity is advected to and further intensified within the downdrafts through conservation of angular momentum.

Simulations by Brandenburg et al. (1996) with an initially weak magnetic field suggested in downdrafts the formation of vertically-oriented vortex tubes with magnetic field wrapped around them, being amplified and maintained by local dynamo processes. Vortex buoyancy causes upwards flows in the cores of the extended downdrafts. Steiner et al. (2010) performed numerical simulation of solar surface convection with the CO^5^BOLD code (Freytag et al. 2012) to interpret observations with IMaX/SUNRISE I that showed granular substructure in the form of bright and dark lanes at the granular boundaries that move together into the host granule. From cross sections through the computational domain of the simulation, it was concluded that these granular lanes are the visible signature of horizontally-oriented vortex tubes forming at the boundaries of granules. The horizontal orientation of these vortex tubes indicates that the baroclinic term of the vorticity equation is probably their source as is explained in the last paragraph of Sect. 5. Numerical simulations by Moll et al. (2011b) also showed horizontal swirls located at the edges of the granules. Their three-dimensional structure consisted of bent and arc-shaped vortex tubes. Even earlier, rapidly rotating swirls that evolve near granular boundaries and form arc-like (thus mainly horizontal) vortices in the photosphere were reported of magnetic-free numerical simulations with the ANTARES code by Muthsam et al. (2010) and, with the Stagger code of Nordlund et al. (1994), by Stein and Nordlund (1998).

Moll et al. (2011b), using dynamo simulations of near-surface solar convection of Moll et al. (2011a) performed with the MURaM code (Vögler et al. 2005), first employed the concept of swirling strength to detect vortices in a solar physical context. They found vortex regions to form a network of highly-intertwined filaments and a depression of the optical depth surface at the photospheric foot-points of the vortex tubes. As an example, they selected a vertically oriented vortex with a diameter of $\sim 80~\text{km}$ protruding through the optical surface. They found a nearly-rigid horizontal velocity at its core (innermost 60 km) and the maximum vertical velocity occurring at the center of the vortex. Both, the gas pressure and mass density were found lower than the horizontal mean at the mean solar surface.

**Fig. 12:**
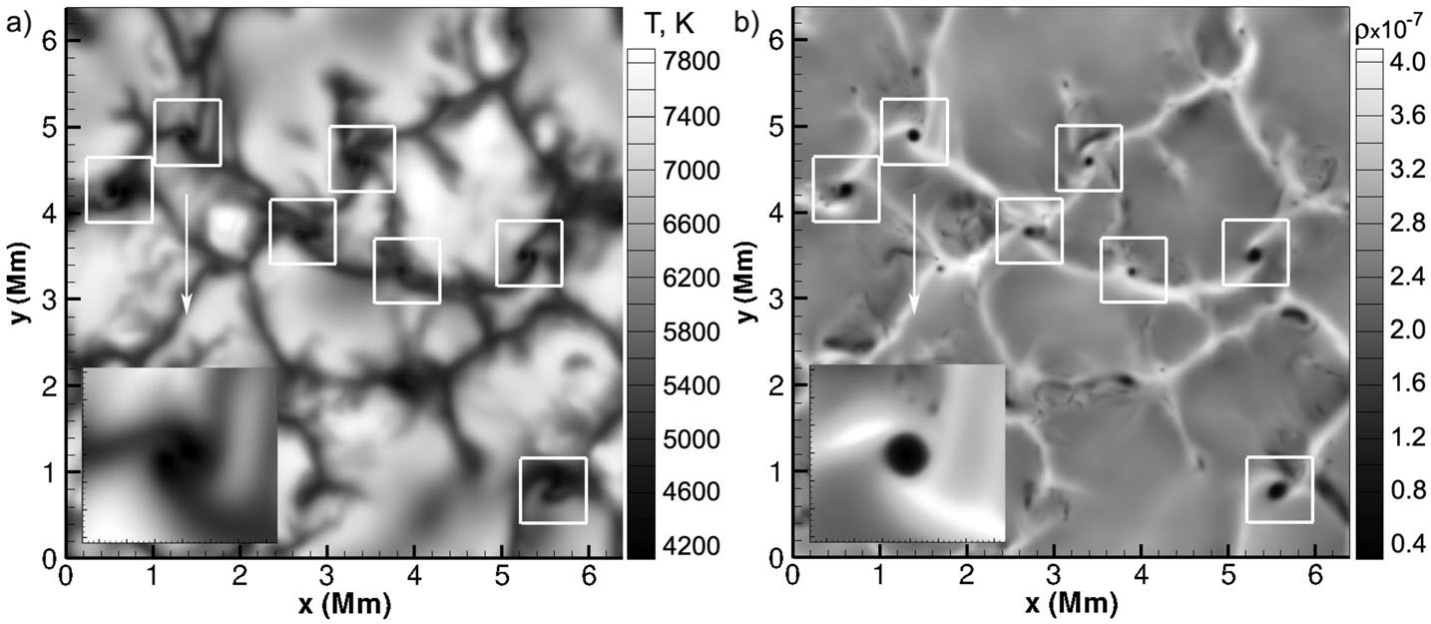
Snapshots of the simulated solar surface temperature (left panel) and density (right panel). The white squares indicate several vortex structures (whirlpools). These are located in the intergranular lanes. The left bottom corner images at each panel show the detailed structure of a magnified whirlpool. From Kitiashvili et al. (2011) ©AAS. Reproduced with permission

Similar findings, namely strong decreases of temperature, density, and pressure in the surface layers of vortex regions were also reported by Kitiashvili et al. (2011) resulting from non-magnetic simulations with the “SolarBox” code (Wray et al. 2015). Figure 12 shows snapshots of the temperature and density of the surface layers from their hydrodynamic run. They indicate several vortices, which structure can be described by common properties such as a low-temperature core, highly decreased density and gas pressure, strong downflows (up to $9~\text{km}\,\text{s}^{-1}$), and high-speed (often supersonic) horizontal velocities, up to $11~\text{km}\,\text{s}^{-1}$. We note that Kitiashvili et al. (2011) also investigated the process of acoustic waves excitation due to vortex interaction that is further discussed in Sect. 8.2.1.

**Fig. 13:**
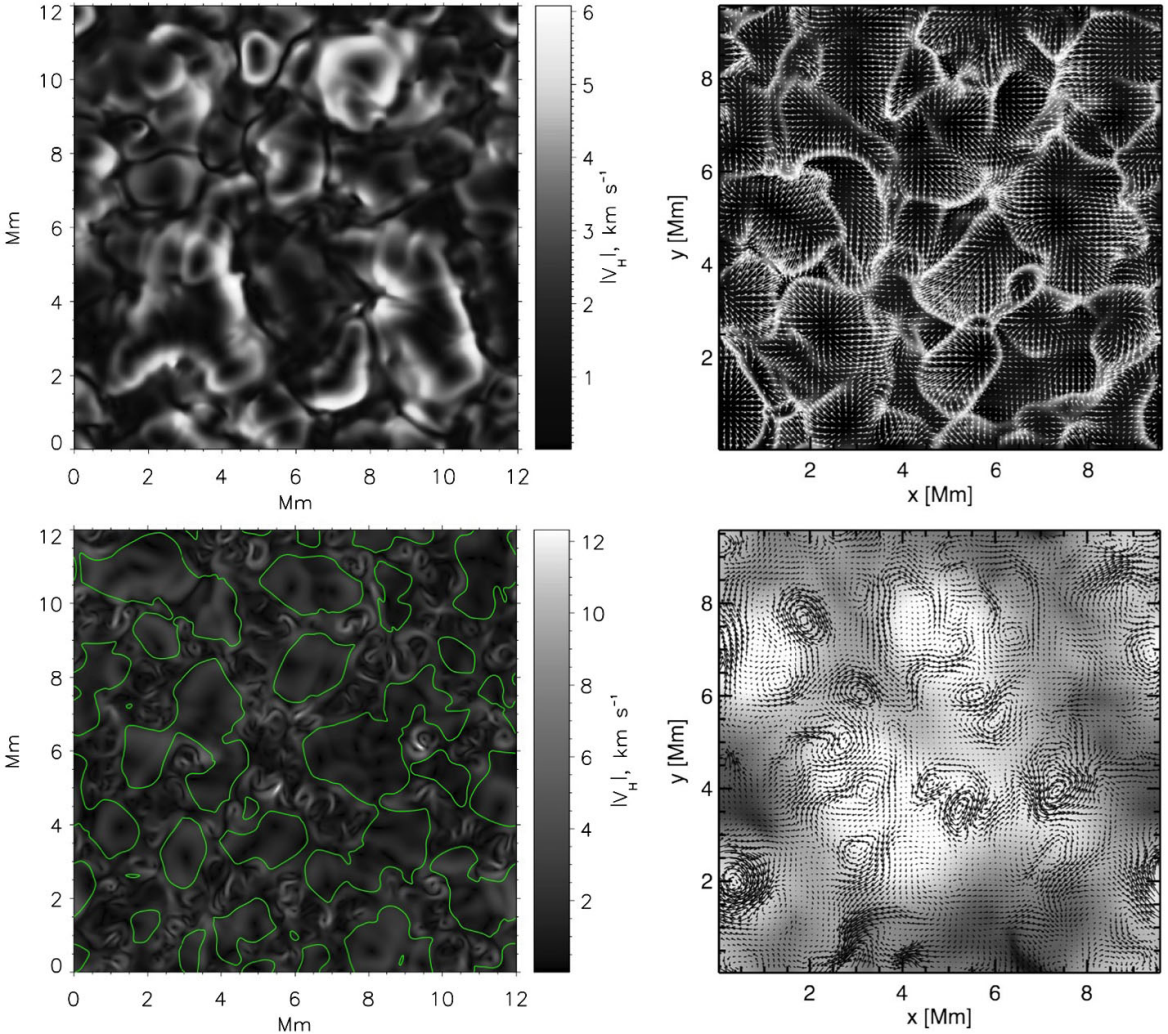
Comparison of non-magnetic (top row) with magnetic simulations (bottom row). The left column panels (Credit: Shelyag et al. 2011b, reproduced with permission ©ESO) show snapshots of the horizontal velocity component in the upper photosphere (contours enclose regions where the vertical magnetic field component is lower than 30 G). The right column panels, adapted from Steiner and Rezaei (2012), show snapshots of the velocity field at a hight of 1300 km above $\tau _{\mathrm{c}} = 1$. Both works suggest that vortices do not appear in the top photospheric and chromospheric layers of non-magnetic simulations but do appear very prominently in the presence of magnetic fields

Non-magnetic simulations by Shelyag et al. (2011b) with the MURaM code (see Fig. 13, left column) also showed the generation of vorticity at the visible solar surface of non-magnetic models, resulting from the hydrodynamic baroclinic term. However, small-scale vortices became much more prominently visible in the upper photospheric layers of respective magnetic simulations (further discussed in Sect. 7.3). Likewise, Steiner and Rezaei (2012) highlighted the absence and abundance of swirls in the chromospheric layers of their non-magnetic and magnetic models, respectively (see Fig. 13, right column). Near and beneath the solar surface, they obtained small-scale vortices within and close to intergranular lanes, very similar to those of Kitiashvili et al. (2011).

Following Calvo et al. (2016), decomposition of the advection term of the Eulerian momentum equation and substitution of a purely rotational velocity field with azimuthal component $v_{\theta}$ leads to a pressure balance equation in the purely hydrodynamic rotating funnel, which can be written as

29 $$ \frac{1}{\rho}\frac{dP}{dr} = \frac{v^{2}_{\theta}}{r}\,. $$

This equation demonstrates that in purely hydrodynamic vortices the pressure gradient is created by the centripetal force and leads to a partial evacuation of the funnel, somewhat similar to the action of the magnetic field in the case of a magnetic flux concentration. In some circumstances this leads to enhanced radiation intensity like in the case of magnetic bright points. Numerical simulations show that vortical downdrafts in intergranular lanes are indeed occasionally associated with enhanced radiative intensity (bright points) even in the absence of magnetic fields (or in the presence of very weak fields) as was first pointed out by Moll et al. (2011b). Their simulation showed that the normalized bolometric intensity is locally increased, making the vortex appear within the dark intergranular lane as a bright spot. Similarly, non-magnetic simulations by Freytag (2013) with the CO^5^BOLD code revealed the existence of bright centers of vortices (his Fig. 6). Calvo et al. (2016) have carried out a statistical analysis of these, so called, nMBPs based on two different magnetic field-free simulations with grid-cell sizes of 10 km and 7 km. The nMBPs in these simulations show a mean bolometric intensity contrast of approximately 20% (with respect to their intergranular surroundings), have a size in the range of 60 to 80 km, and show a depression of the unity optical depth isosurface by 80 to 100 km. Swirling downdrafts provide, by means of the centripetal force, the necessary pressure gradient for the formation of a reduced mass density funnel extending from the subsurface layers into the photosphere. We note that similar frequently occurring funnels that do not, however, manage to reach into the photosphere do not produce bright points.

Calvo et al. (2016) conclude that the resolving power of a 4 m-class telescopes, such as the Daniel K. Inouye Solar Telescope (DKIST), is needed for an unambiguous detection of these objects (see, e.g., Rast et al. 2021). As of yet, however, no detailed radiation transport modelling has been carried out for nMBPs and it is not clear if simulations of yet higher spatial resolution than those of Calvo et al. (2016) and Freytag (2013) will still expose nMBPs, as convergence of the size of their vortices was not yet achieved.

NMBPs caused by swirling downdrafts were also found in simulations of stellar atmospheres from spectral types F5 to K8 by Salhab et al. (2018). In particular the spectral type K2 showed most prominent nMBPs.

### Vortex Motions in Magnetoconvection Simulations

Apart from observations and magnetic field free numerical simulations, vortex flows have also been detected in magnetoconvection simulations generated with different radiative MHD codes (see Table 6). Despite the variety of numerical schemes and diffusivity mechanisms of these codes, they produce similar flow and magnetic field structures.

Vortex flows are generated in the photosphere as a result of turbulent convection and the bathtub effect (see Sect. 5) on the cooled plasma fluid sinking into the intergranular lanes. Therefore, pure hydrodynamic simulations, encompassing the surface layers of the convection zone ($\sim 1\text{--}2~\text{Mm}$ below the mean visible solar surface) and the photosphere ($\sim 100\text{--}200~\text{km}$ above the mean solar surface) are sufficient to create vortex flows in the photosphere. However, magnetic fields are required for the propagation of this vorticity to the outer solar atmospheric layers in the form of torsional Alfvén waves. These upwardly propagating MHD waves carry significant energy with them that can potentially be dissipated in the upper layers of the solar atmosphere, making vortex flows a potentially important source for heating of the solar atmosphere.

As mentioned in Sect. 6.2, the locations of swirling motions are first of all observed to be associated with intergranular lanes and a similar connection is found in simulations (Stein and Nordlund 1998; Nordlund et al. 2009; Kitiashvili et al. 2011; Shelyag et al. 2011b). The primary source of enhanced vorticity in intergranular lanes is the baroclinic term in the equation of vorticity (see Eqs. (7) and (8)). Baroclinicity becomes large near the edges of granules as well as in the downdrafts. The diameter (horizontal size) of vortices is strongly influenced by the spatial resolution and the vortex detection method. Since most (almost all) of the existing vortex detection methods in use for the analysis of numerical simulations make use of velocity gradients, they naturally select smallest possible vortex regions given by the spatial resolution of the simulation data. Therefore, Moll et al. (2011b) found vortices with a diameter of approximately 80 km, which is beyond the current observational limits. However, they could not detect larger vortices that have been widely observed and can be much larger than the typical width of intergranular lanes (Bonet et al. 2008; Brandt et al. 1988). Small-scale vortices of diameters less than 80 km in the photosphere are much smaller than previously observed photospheric vortices. However, being abundant throughout the solar surface in simulations of various magnetic environments, they appear much more frequent than the larger events, indicating that the net energy and mass transport by them may be significant in the energy and mass-flux balance of the solar atmosphere.

Shelyag et al. (2011b) carried out numerical simulations using the MURaM code and compared photospheric vortices in the non-magnetic and the magnetic cases. They performed a detailed analysis of vorticity, in particular on the different physical mechanisms that generate vorticity in the non-magnetized and the magnetized photosphere. They showed that vorticity generation in magnetic field concentrations is physically different from hydrodynamic simulations. In the non-magnetic case, vorticity is generated mainly by the baroclinic term, while in the magnetic case, it is magnetic tension that dominates over baroclinicity in the chromosphere. They also demonstrated a direct correspondence between photospheric vortices and the rotation of G-band bright points arising from strong intergranular magnetic flux concentrations. Although, their simulation box was limited up to 600 km above the mean visible solar surface, they detected vortex motions in the upper part of the box, hinting at chromospheric swirls and their connection with photospheric vortices.

**Fig. 14:**
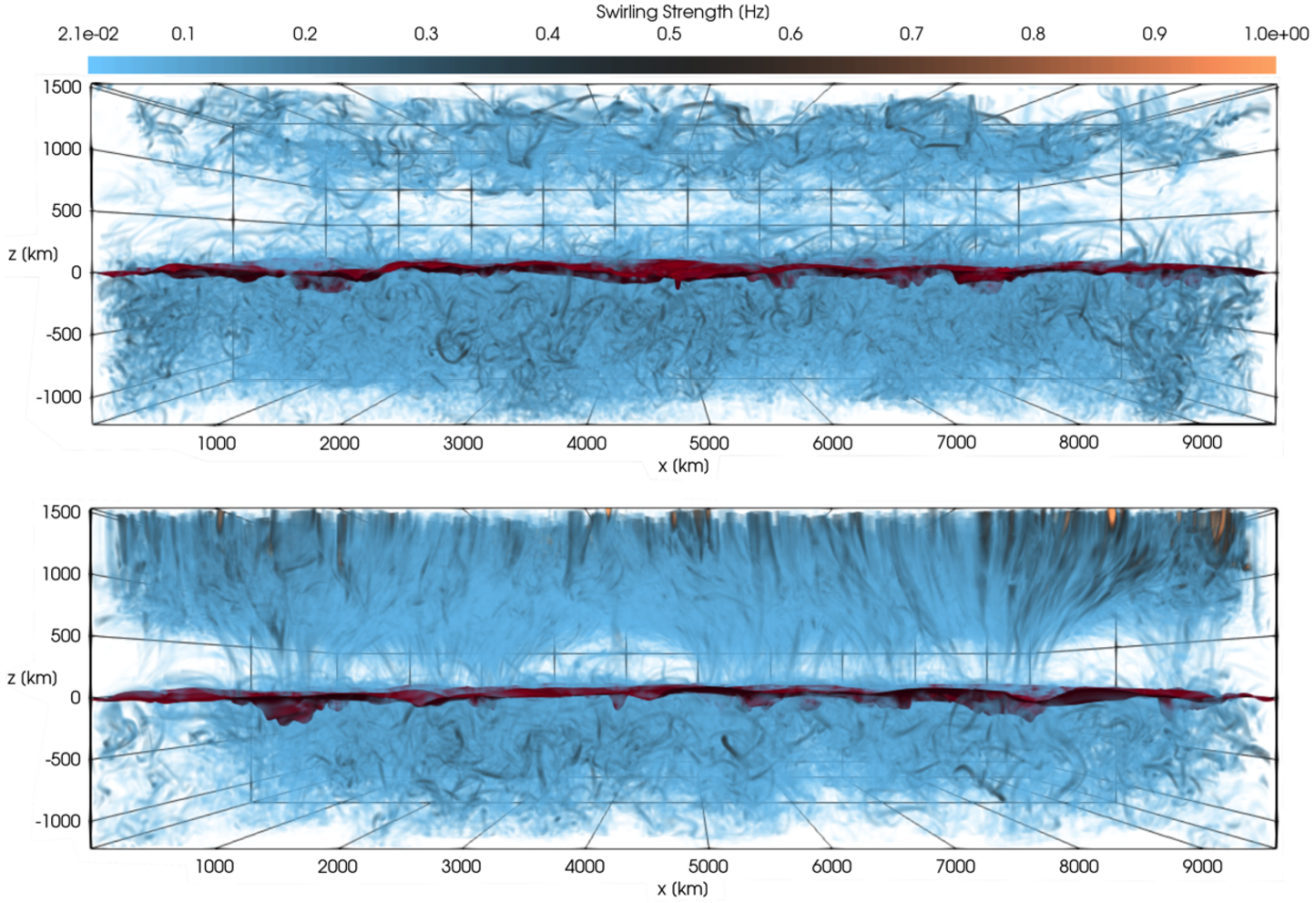
Swirling strength in a simulation domain of $9.6~{\text{Mm}}\times 9.6~{\text{Mm}}$ horizontal extent reaching from the top of the convection zone into the chromosphere over a height range of 2.8 Mm. The optical depth surface $\tau _{500} = 1$ (dark red surface) is located approximately in the middle of the height range. Top panel: magnetic field-free simulations. Bottom panel: Simulation that started with a homogeneous, unipolar magnetic field of a strength of 50 G. Credit: adapted from Battaglia et al. (2021), reproduced with permission ©ESO

Using the MURaM code, Moll et al. (2012) compared photospheric vortices of magnetic and non-magnetic simulations and discussed the associated local heating. They found the flow structures of vortices to be similar in the deep and the near-surface layers, however, in the upper photospheric layers, they appeared vastly different. In the non-magnetic simulations, vortices did not extend high into the atmosphere but bend near the optical surface, forming loops, whereas, in the magnetic case, vertically orientated vortices extend over the entire height range of the simulated photosphere (see also Fig. 14). Both these structures were found closely connected with local heating. In the non-magnetic case, shock heating was found the primary heating mechanism for local heating in the upper layers of the atmosphere, while this mechanism is suppressed in the magnetic case due to the presence of strong magnetic fields. On the other hand, in the magnetic case, a combination of Ohmic heating in the lower to middle photosphere and viscous heating in the upper photosphere was found as the dominating source mechanisms causing the local heating over vortex locations. They found that the presence of strong magnetic fields also affects the rotation velocity of vortices and the local pressure over vortices. They suggested that in the magnetic case, the locally lowered pressure over vortices is a combined results of magnetic flux concentrations and the centrifugal force. Also, the typical swirling periods are much smaller and vortices rotate faster in the magnetic case than in the non-magnetic case. These results are consistent with the findings by Shelyag et al. (2011b) who analyzed the generation mechanisms of photospheric vortices in magnetic and non-magnetic simulations and arrived at the conclusion that the vorticity is generated more efficiently in the magnetised model.

While vorticity is the standard quantity to study vortical flows in fluid dynamics, it does not distinguish shear flows from an actual vortical flow in the sense as defined in Sect. 2, and vice versa, actual vortical motion may exhibit vanishing vorticity as was demonstrated in Sect. 3.2. Swirling strength and the swirling strength vector as introduced in Sect. 4.6.4 are physical quantities that identify actual vortical flow much more precisely than vorticity does. The top panel of Fig. 14 displays the swirling strength in the computation domain of the purely hydrodynamic simulation of Calvo et al. (2016), which encompasses the height range from the top of the convection zone to the chromosphere over a height range of 2.8 Mm. Swirls of all directions can be seen in the convection zone and the tenuous top layers that represent the chromosphere. The stably stratified photosphere shows a distinct lack of swirls with the few ones having preferentially the form of arc-shaped and horizontally directed filaments close to the $\tau _{500} = 1$ surface. These largely represent the horizontally directed vortex tubes at the edges of granules discussed in Sect. 6.2 (Steiner et al. 2010). This picture changes drastically when analyzing a simulation with an initial homogeneous, unipolar magnetic field of a strength of 50 G (Fig. 14, bottom panel). The filamentary structure of the swirling strength is now shaped by the magnetic field; the filaments are largely aligned with the vertically directed magnetic field in the top layers. The funnel shaped bundles of swirling strengths in the photosphere coincide with the funnel shaped magnetic flux concentrations, suggesting that the swirls in the photosphere and the chromosphere are generated by the Lorentz force of the interacting plasma and magnetic fields.

**Fig. 15:**
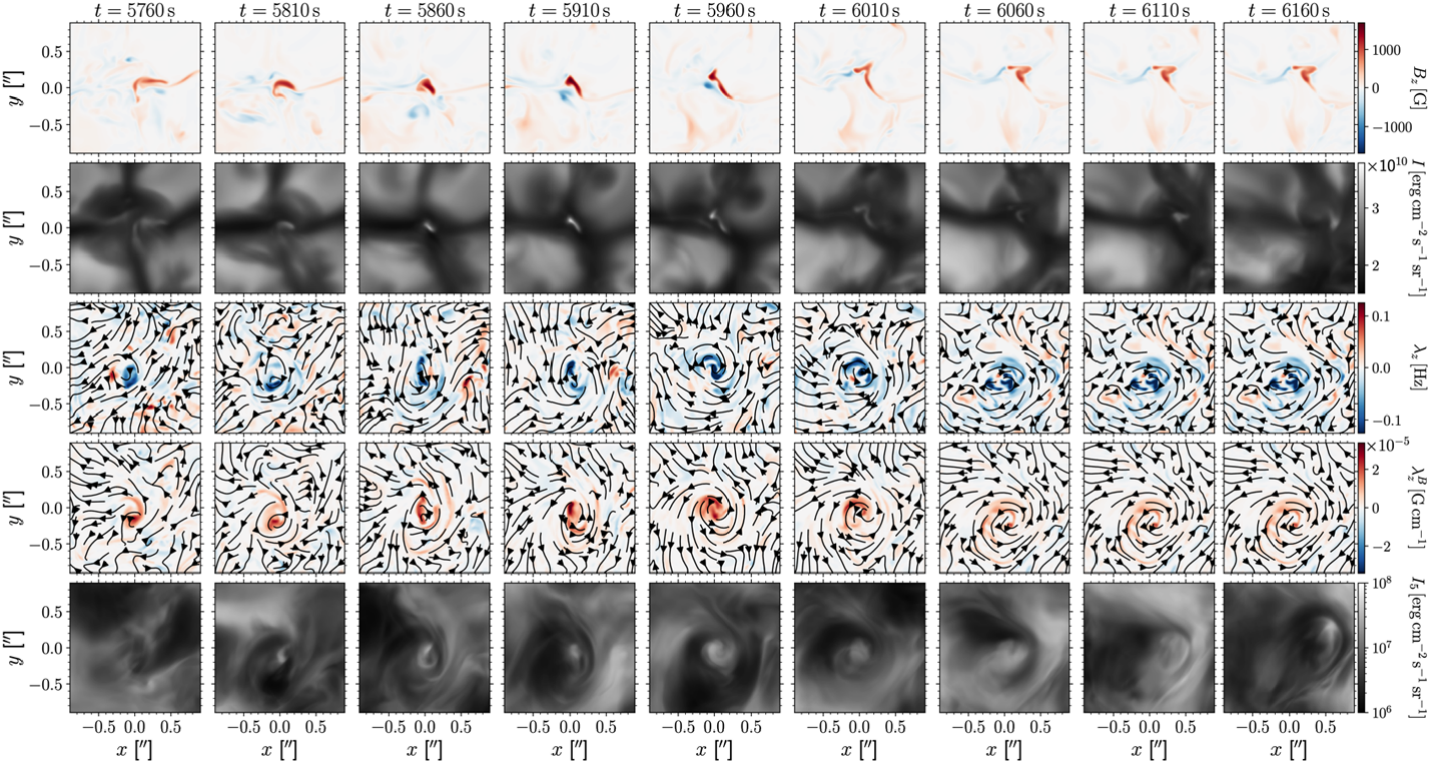
Time sequence of a simulated swirl event. Rows from top to bottom show: vertical component of the magnetic field $B_{z}$ at mean visible optical depth $\tau _{500} = 1$ (magnetogram) with red positive and blue negative polarity, continuum intensity $I$, vertical component of the swirling vector $\lambda _{z}$ at a height 700 km above $\tau _{500} = 1$, corresponding magnetic swirling strength $\lambda ^{B}_{z}$ at the same height level, bin 5 intensity $I_{5}$ as a proxy for the intensity in the core of a chromospheric spectral line. Concerning $\lambda _{z}$ and $\lambda ^{B}_{z}$, blue is clockwise and red is counterclockwise rotation. The maps for $\lambda _{z}$ and $\lambda ^{B}_{z}$ show also streamlines of the horizontal component of the velocity and the magnetic field, respectively. Credit: adapted from Battaglia et al. (2021), reproduced with permission ©ESO

Looking at the origin and propagation of typical chromospheric swirls of simulations, Battaglia et al. (2021) find two classes of events: unidirectional swirls that propagate from their magnetic footpoints with Alfvén speed to the chromosphere, and superposition of such swirl events that lead to more complex swirl patterns in the chromosphere. Figure 15 shows a time sequence of a single swirl event from a simulation with an initial homogeneous, unipolar magnetic field of a strength of 50 G and a data output cadence of 10 s. The top row represents the magnetogram near the surface of optical depth $\tau _{c} = 1$ consisting of a main component of positive (red) and a minor component of negative (blue) polarity. The maximum field strength of the main component at around time $t=5900~\text{s}$ is between 1100 and 1500 G. The second row shows the corresponding continuum intensity maps in which the main magnetic flux concentration is visible as a feeble magnetic bright point. In the third row, in blue and red colors, is the vertical component of the swirling vector, $\lambda _{z}$ (see Sect. 4.6.4 for its definition), at a height 700 km above the level of $\tau _{c} = 1$. Streamlines of the horizontal component of the velocity field are shown as well. From this, we immediately see that the rotation over the time period of 270 s, from appearance to the begin of disintegration of the swirl, is clockwise unidirectional, non-oscillatory. The maximum value of $\lambda _{z}$ is approximately 0.3 Hz in the center of the swirl and at around $t=5960~\text{s}$, corresponding to a full rotation within 42 s. The fourth row shows the magnetic swirling strength as explained in Sect. 3.3, which is a measure of the amount of twist in the magnetic field. It still refers to the reference height 700 km. Streamlines of the horizontal component of the magnetic field are shown as well. The bottom row displays the bin 5 intensity maps of the non-grey simulation, which can be taken as a proxy for the radiative intensity from chromospheric layers. It clearly displays the evolution of a chromospheric swirl similar to the observed one of Fig. 10c. Establishing a time-distance plot of $\lambda _{z}$, taking the distance to be the height in the atmosphere, it reveals that the swirl propagates with Alfvén speed and with unchanged sense of rotation from the $\tau _{c} = 1$ level up to the chromosphere. Evidently, the swirl originates from the clockwise rotation of the main component of the magnetic flux concentration. This rotation is clearly visible from the first four magnetograms in the top row of Fig. 15. Also evident from time distance plots is that the magnetic field has rotation (a torsional component) opposite to the swirling motion for an upwardly moving swirl and taken the vertical component of the magnetic field to be positive, which clearly is the property of a propagating torsional Alfvén wave (see, e.g., Priest 2014, Chap. 4.4). However, caution is indicated when working with time distance plots of fixed detection slice: neighbouring swirls of opposite sense of rotation may move in and out of the slice giving the impression of an oscillatory torsional Alfvén wave.

In fact, magnetic knots, larger than the small-scale magnetic flux concentration of Fig. 15 harbour many swirls of various sense of rotation and strength, often close together. In this case, the magnetic footpoint is not rigidly rotating as a whole, but has sub-structure of different relative motion and rotation. As the magnetic field spreads out with height in the atmosphere, so do these swirls as they propagate in the upward direction. In the chromosphere they start to merge giving raise to a complex pattern of rotational and undulatory motion. Qualitatively, the analysis of Battaglia et al. (2021) confirms the cartoon picture of Fig. 6 (Wedemeyer and Steiner 2014) but shows beyond that how unidirectional rotation is propagating with Alfvénic speed in the form of a torsional wave package from the bottom of the photosphere up into the chromosphere.

Regarding isolated swirls, Battaglia et al. (2021) did not find any cases of torsional oscillatory behaviour within a time span of about 400 s. This may hint at the bathtub effect, which is also unidirectional, as the mechanism exciting torsional Alfvén pulses. While Battaglia et al. (2021) have demonstrated the Alfvénic propagation of these pulses, their precise origin and excitation remains yet to be identified. Also, chromospheric swirls are not seen to torsionally oscillate (Wedemeyer-Böhm et al. 2012).

**Fig. 16:**
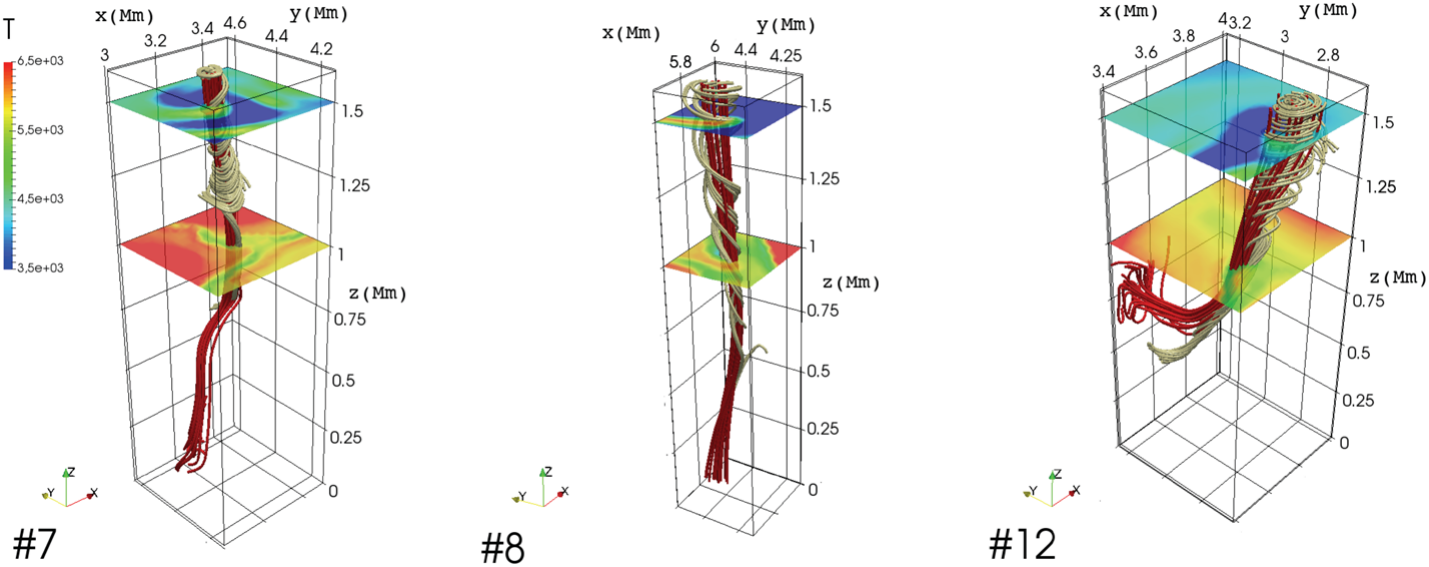
Three different vortices identified in MURaM simulation are shown. Red and dark khaki colours show respectively magnetic and velocity field lines traced within the vortex boundary. The $xy$-planes (colored by the plasma temperature) are placed at $H = 0~\text{Mm}$ (or $z = 1.0~\text{Mm}$) and $H = 0.5~\text{Mm}$ (or $z = 1.5~\text{Mm}$). From Silva et al. (2020) ©AAS. Reproduced with permission

**Fig. 17:**
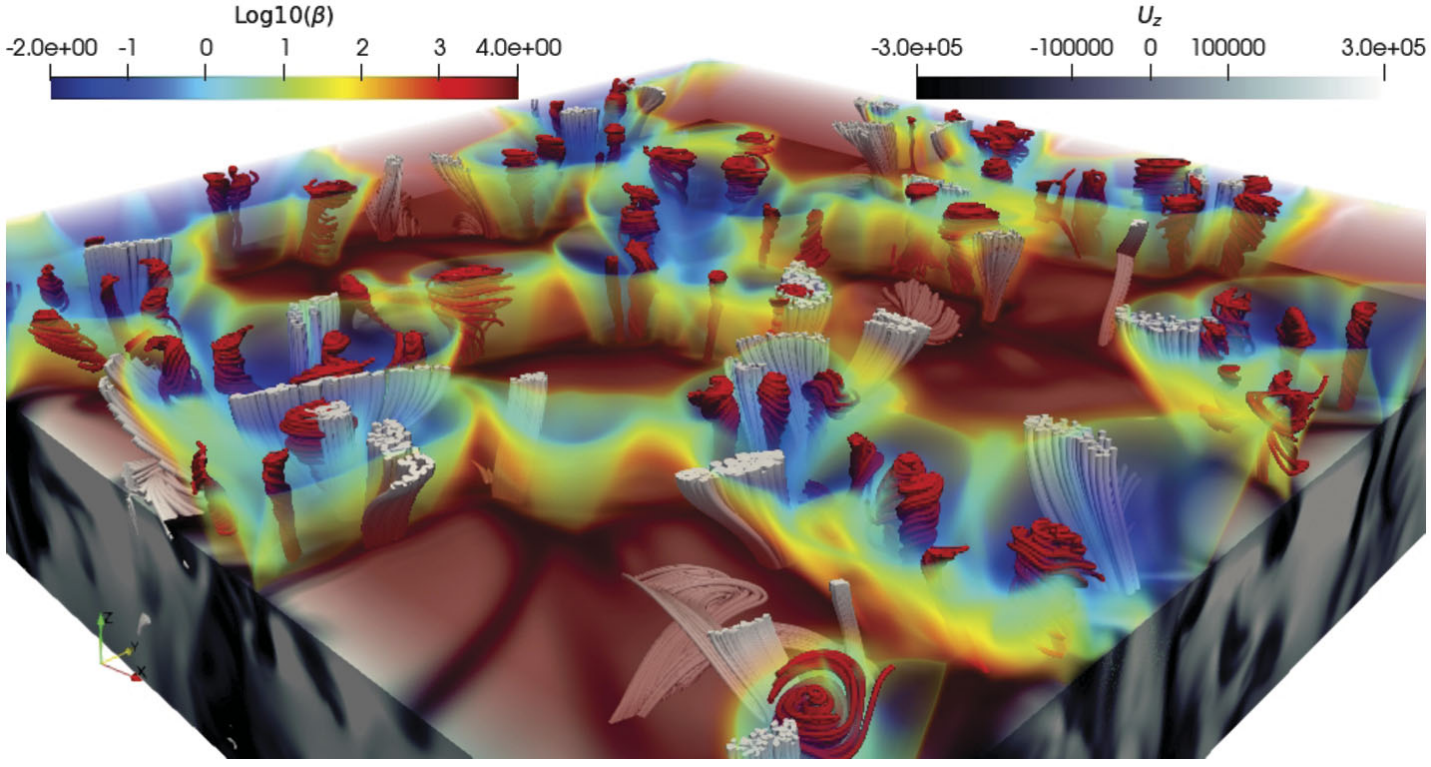
Three-dimensional representation of the photospheric and subphotospheric domain of $6 \times 6 \times 1.6~\text{Mm}^{3}$ size of a magneto-convection simulation generated with the MURaM code. The colored volume rendering gives the logarithm of plasma-$\beta $. The $K$- and $M$-vortices are colored in red and gray, respectively. The bottom part of the image, i.e., the region below the solar surface, seen as vertical sections at the domain boundaries, shows the vertical component of the velocity field in grays scales. From Silva et al. (2021) ©AAS. Reproduced with permission

**Fig. 18:**
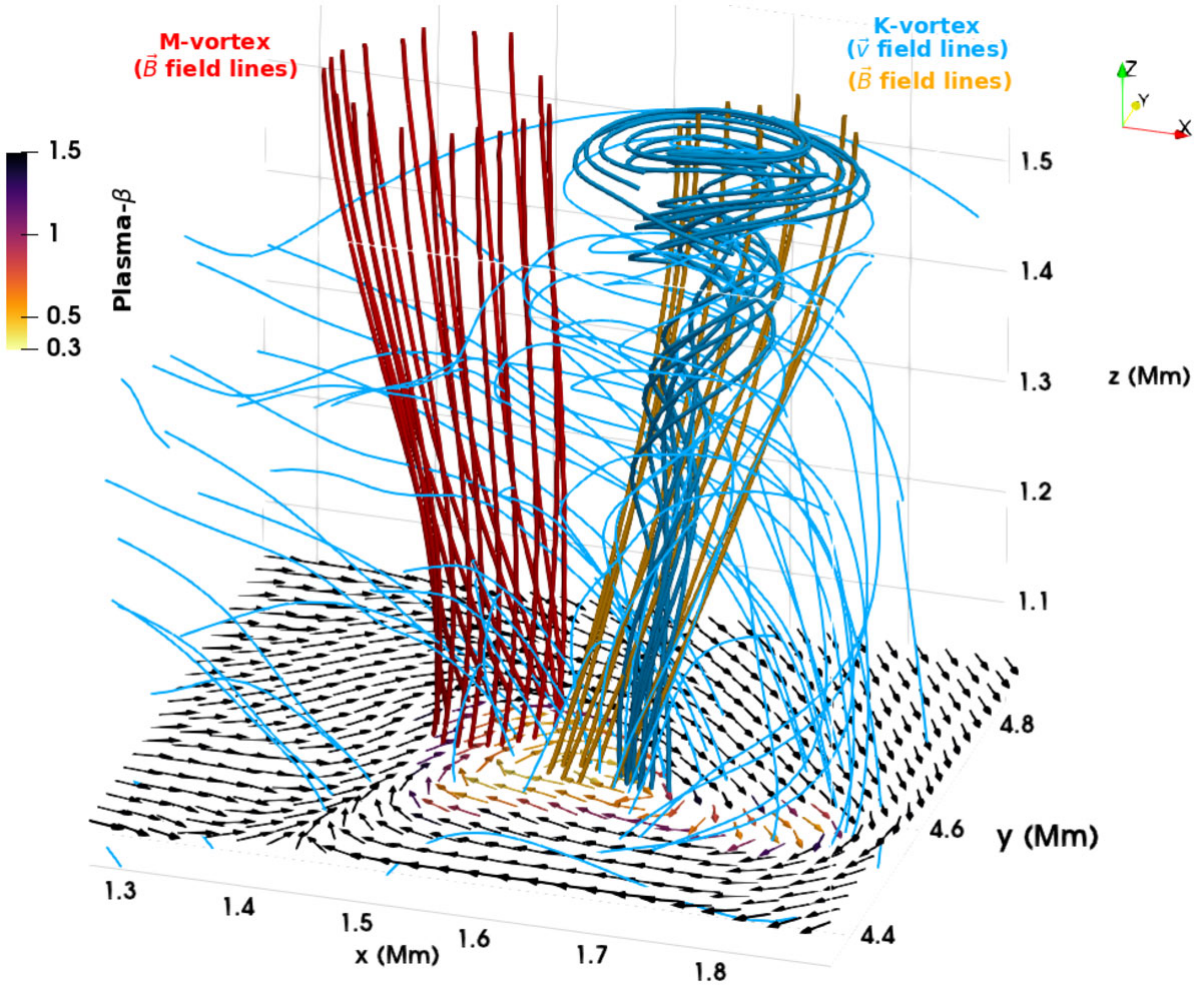
Close-up of the MURaM simulation analysed by Silva et al. (2020) at time $t=25~\text{s}$, showing two types of coherent vortical structures in the lower solar atmosphere. The blue velocity streamlines indicate fluid motion. Thicker streamlines (on the right side of the figure) represent a $K$-vortex within practically untwisted magnetic field lines, shown in orange color. The twisted magnetic field lines (in red color on the left side of the figure) represent the $M$-vortex. Note that no coherent velocity field is present at the $M$-vortex location. At the bottom of the figure, the direction of the horizontal component of the velocity vector field is indicated by arrows color-coded with the value of the local plasma-$\beta $. The $K$-vortex is formed in a region of low plasma-$\beta $ and strong rotational flow. The $M$-vortex is rooted where $\beta > 1$ and the flow has a shearing behavior. The $K$-vortex was detected using IVD, the M-vortex using IACD (see Silva et al. 2021). The figure has been courteously produced by Dr. Suzana Silva (Plasma Dynamics Group, Department of Automatic Control and Systems Engineering, The University of Sheffield, UK; e-mail: suzana.silva@sheffield.ac.uk) for the purposes of this review

Silva et al. (2020), using MURaM magnetoconvection simulations, selected three vortices located in different parts of the computational domain for the analysis of the instant three-dimensional flow and magnetic field structure. Figure 16 shows the magnetic field lines and velocity stream lines in red and gray colours, respectively. The horizontal $xy$-planes at $H=0~\text{Mm}$ and $H=0.5~\text{Mm}$ show the temperature at the corresponding geometric height, where $H=0$ corresponds to the visible solar surface of Rosseland optical depth $\tau _{R} = 1$, located 600 km below the top boundary of the computational domain. From these temperature maps it can be seen that vortical motion strongly influences the mixing of hot and cold plasma. It was also found that the vortex tangential velocity as a function of distance from the axis of the vortex can be best fitted with a cubic polynomial. Recently, Silva et al. (2021), using the same MURaM simulations, have shown that kinetic vortices ($K$-vortices) appear in regions of mainly vertical magnetic fields and do not give rise to magnetic vortices ($M$-vortices) as can be seen from Fig. 17. Figure 18 shows a close view of a $K$- and $M$-vortex from the MURaM simulation that was analysed by Silva et al. (2020). $K$-vortices mainly occur in plasma regions of low plasma-$\beta $. In these regions the magnetic tension prevents significant twisting of field lines in small-scale magnetic elements, which have strong (i.e., kilogauss) magnetic field strength. $M$-vortices, on the other hand, appear in regions of relatively weak magnetic field strengths (i.e. plasma-$\beta > 1$) and shear flows. We note that in both aforementioned studies, vortices have been identified with the IVD method described in Sect. 4.4. A differentiation between kinetic and magnetic swirls in terms of the swirling strength and the magnetic swirling strength was carried out by Battaglia (2020) and Battaglia et al. (2021).

**Fig. 19:**
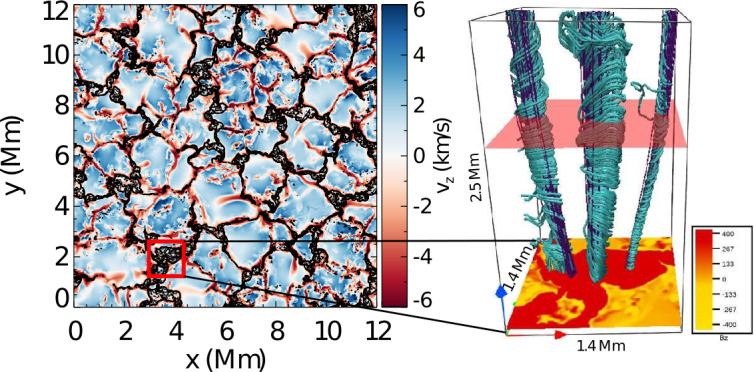
The vertical component of flow velocity at the continuum formation height (left panel) with black contours showing the vertical component of the magnetic field that ranges from 400 G to 2500 G. The red box in the left panel highlights a magnetic element whose 3D view is shown in the right panel. The bottom image of the box indicates the vertical component of the magnetic field (vertical bar) and sea green and deep purple lines correspond, respectively, to velocity streamlines and magnetic field lines at the selected vortex locations. Credit: Yadav et al. (2021), reproduced with permission ©ESO

With increasing computational capabilities smaller vortices are being detected in simulations. However, when vortices are generated due to photospheric turbulence, they are bound to exist over a range of scales (limited by the resolution and size of the computational domain of the simulation). To identify vortex motions present at the various spatial scales, Yadav et al. (2020) performed high-resolution simulations (10 km grid spacing in all three spatial directions) of a uniform solar plage region using the MURaM code. They then used the full-resolution data to detect vortices of the smallest spatial scales present in their simulations. They used the swirling strength criterion to detect vortices. Next, they degraded the high-resolution simulation data to achieve a coarser effective spatial resolution providing information about vortex flows at a comparatively larger spatial size. They reported, for the first time, vortices to exist from diameters of 50 km up to 2 Mm in simulations. Although the smallest vortices have lifetimes of less than a minute with randomly distributed direction of rotation, underlying larger vortex flows stay longer ($\sim 5~\text{min}$) and keep rotating in a fixed direction during their lifetime proving that larger vortices are not the residual effect of stronger and smaller vortices but rather true vortex flows of their own. The smallest vortices obtained in their simulations (as shown in Fig. 19) have a spatial size smaller than the current observational limits but they should become accessible in the future with improved instrumentation.

The spectropolarimetric observations of horizontal vortex tubes by Fischer et al. (2020), were accompanied by magnetohydrodynamic numerical simulations. These simulations showed cases in which the horizontal vortex tube of the granular lane is capable of recirculating magnetic flux within a granule, transporting it to the solar surface, where it causes a polarimetric signature similar to what is observed. Thereby, the magnetic topography can become quite complex, showing a core of lane-aligned magnetic field (causing mainly the linear polarization signal) being wrapped by a weaker torsional component that arises from the vortex flow (see their Fig. 3 and movie). From this, the authors suggest that horizontal vortex tubes may constitute an important component of the small-scale dynamo mechanism that supposedly acts at the quiet-Sun surface. Shallow recirculation of magnetic flux by action of vortex tubes was also found by Rempel (2018) in numerical simulations of exploding granules.

Recently, Aljohani et al. (2022) proposed a new approach to derive essential information for the analysis of MHD wave excitation and propagation on vortices surfaces. Their methodology is based on a) identification of the vortex center in 3D MuRAM magneto-convection simulations by applying the LAVD method and construction of the vortex surface boundary by using the advection of fluid elements, and b) mapping of this vortex surface onto a cylindrical envelope grid surrounding it, for the study of vortex-related plasma parameters as functions of space and time. They found that spatial and temporal changes in density and pressure occur within the whole vortical structure while changes of other physical quantities that describe the state of the plasma, dynamics, and energetics, such as temperature, plasma-$\beta $, and velocity, are more localized. The analysis of the MHD Poynting flux showed that the majority of the energy is directed in the horizontal direction (see, e.g., Silva et al. 2022). Moreover, their study indicates that the forces that drive the dynamics of plasma on the vortex surfaces, i.e. pressure and magnetic forces, are not in balance and, therefore, vortices do not rotate as rigid bodies.

### Vorticity in Jets and Eruptive Phenomena Associated with Vortices

The chromospheric region between the photosphere and corona is filled with various jets. Such jet-like phenomena, observed in a wide range of wavelengths, from the optical (e.g. $\text{H}\alpha $, Ca lines) to EUV and X-rays and usually identified as spicules on the solar limb and mottles, dynamic fibrils and “straws” on the solar disc (see review by Tsiropoula et al. 2012, for further details) represent small-scale ejections of photospheric/chromospheric material into the corona. It is unknown if they have a common physical mechanism and the relationship between these phenomena with different plasma temperatures is unclear.

Many numerical studies have shown that vorticity can be generated in jets such as spicules due to motions in the Sun’s lower atmosphere. In a study by Martínez-Sykora et al. (2013), the resulting simulated jet vorticity is due to compression at the footpoint of the jet. This compression causes the magneto baroclinic term in the equations to generate vorticity. However, simulations by González-Avilés et al. (2018, 2019) did not include magnetoconvection yet rotational motions still naturally occurred. This finding is important in the context of coronal heating, as it provides evidence that torsional (Alfvén) waves may be excited directly in the corona where they can dissipate energy rather than having to navigate the highly inhomogeneous solar atmosphere.

Kuridze et al. (2016) and Iijima and Yokoyama (2017) have shown that vortices can drive observed chromospheric jet-like features, such as spicules, with the Lorentz force of the twisted magnetic field lines playing an important role in their production. Numerical studies by Snow et al. (2018) have indicated that vortex motions at photospheric layers can generate magnetic substructures and drive shocks between merging flux tubes higher in the atmosphere. These shocks may act as a driving mechanism for jets such as spicules and also contribute to heating of the chromosphere. In this model, the independent flux tubes expand with height in the atmosphere where they eventually merge into one tube at chromospheric heights. The oppositely directed vortex driving motions of each tube acting in the photosphere, pass through each other at the merging height and generate a rotational motion, leading to thin short lived substructures which may be interpreted as jets. The interacting vortex motions also reduce the mass density at the merging height of the two tubes and generates a superposition. The continued vortex driving increases the amplitude of the superposition until it exceeds the local sound and Alfvén speeds creating shocks. We note, however, that a comprehensive study is necessary to establish any further possible relationship between vortex flows, formed inside the downflowing intergranular lane, and strong plasma upflows related to jet-like features observed higher up.

**Fig. 20:**
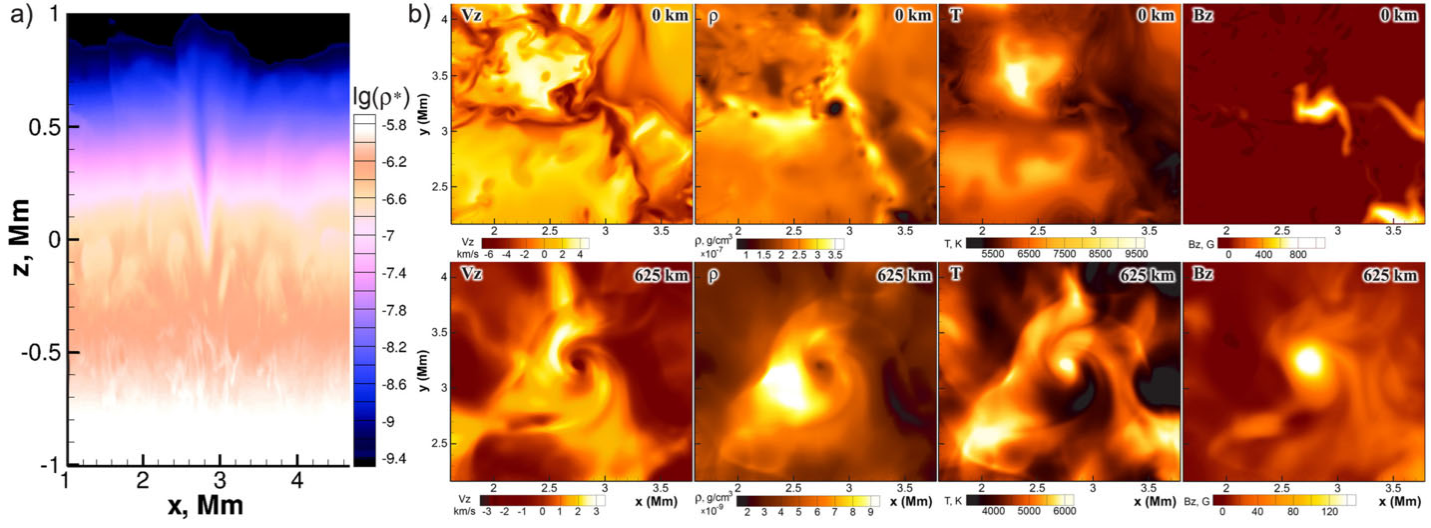
Example of a quiet-Sun simulation illustrating a complicated dynamical structure in the chromospheric layers around a vortex tube, which extends from the subsurface layers into the photosphere and chromosphere. Panel **a**) shows a vertical section of the logarithmic density. The vertical thin structure in the center with a low-density core is a vortex tube with a concentrated and twisted magnetic field, reaching into the chromosphere. It produces mass ejections and shock waves in the surrounding plasma, illustrated in panels **b**), which show from left to right the vertical velocity, density, temperature, and vertical magnetic field in two horizontal slices: at the solar surface (top row) and 625 km above the surface (bottom row). Adapted from Kitiashvili et al. (2013) ©AAS. Reproduced with permission

Recently, it has also been suggested from numerical simulations as well as from observations with IRIS, SST, and the Goode Solar Telescope (GST) that eruptive, jet-like phenomena can be driven by magnetized vortices (small-scale swirls, or “magnetic tornados”) generated in the intergranular lanes. Yurchyshyn et al. (2011) observed that intergranular jets, originating in the intergranular space surrounding granules, tend to occur at locations of granular lanes, which in turn are associated with the formation and evolution of horizontal vortex tubes within individual granules. Simulations by Kitiashvili et al. (2012b, 2013) reveal that vortex tubes can penetrate into the chromosphere and strongly affect its thermodynamic properties. As Fig. 20 demonstrates, the photospheric temperature is lower in the vortex core than the mean temperature in the near-surface layers, whereas at the base of the chromosphere, $\sim 600~\text{km}$ above the photosphere, the temperature is higher. The vortex tube expands with height, and its diameter reaches 1 Mm in the chromosphere in accordance with observations of such chromospheric helical structures (see Sect. 6). Penetration of the vortex tube into the atmosphere causes notable thermodynamic changes in the low chromosphere; it involves surrounding plasma into helical motions, resulting in additional accumulation of energy and mass. The vortex core is characterized by a relatively low gas pressure, creating strong pressure gradients. As Kitiashvili et al. (2013) have shown, the continuous build-up of kinetic energy and magnetic and gas pressure in the low atmosphere (temperature minimum layers) leads to the formation of a highly twisted ring-like structure which becomes unstable and erupts (see Fig. 20).

To identify sources of these eruptions, it is convenient to use the following form of the momentum equation:

30 $$ \frac{\partial \boldsymbol{v}}{\partial t}+( \boldsymbol{v}\cdot \nabla ) \boldsymbol{v}= \frac{\boldsymbol{J}\times \boldsymbol{B}}{c\rho}- \frac{\nabla p}{\rho}- \boldsymbol{g}, $$

where $\boldsymbol{v}$ and $\boldsymbol{B}$ are the velocity and magnetic field vector respectively, $\boldsymbol{J}$ is the electric current density, $p$ is the gas pressure, $\rho $ is the density, and $\boldsymbol{g}$ and $c$ are the gravitational acceleration and the speed of light, respectively. The left-hand side of this momentum equation describes the acceleration of a fluid particle and the right-hand side the contributions of the Lorentz force, the pressure force, and the gravitational acceleration. Comparing the different term, it was found that in the subsurface and near-surface layers the mean velocity fluctuations are correlated with the variations of the gas pressure gradient, whereas magnetic field effects have no such correlation, and the amplitude of the variations is negligible (Kitiashvili et al. 2013). In the photospheric layers, the correlation of the Lorentz force with the velocity fluctuations becomes noticeable, but still its magnitude is much smaller compared to the pressure gradients. In the layers, starting near the temperature minimum, the magnetic field effects are comparable with the pressure gradient effects or dominate. This causes flow acceleration by the Lorentz force in the mid chromosphere. Thus, the simulations of Kitiashvili et al. (2013) reveal flow patterns with high speeds, and complex thermodynamic and magnetic structures in erupting vortex tubes. Strong pressure gradients in the near-surface layers, as explained above, initiate and drive such quasi-periodic spontaneous flow eruptions that are further accelerated by the Lorentz force in the chromosphere. These flow eruptions generate Alfvénic waves and transport mass and energy into the solar atmosphere.

## Oscillations and Waves in Vortices

High-resolution observations (Sect. 6) and realistic numerical simulations (Sect. 7), suggest ubiquitous small-scale vortex tubes to play a substantial role in the dynamics of the solar atmosphere from the turbulent convective layers up to the low corona (Sect. 5). In combination with magnetic fields, they can excite, transmit, and convert various types of MHD waves and act as a waveguide, carrying motions and energy upwards. The interaction of vortex flows with magnetic flux tubes is a subject of intensive research in solar physics and maybe key to understanding the energy budget of the solar atmosphere.

In this section, we discuss observations as well as simulations and (magneto)hydrostatic models of wave excitation and propagation in vortices. Moreover, we discuss estimates of energy transfer in vortex motions and spectropolarimetric diagnostics of simulated vortex flows.

### Observational Signatures of Vortex-Related Oscillations and Waves

The oscillatory behavior of vortex flows and corresponding detection of periodic signals reflect various, often difficult to disentangle, physical processes occurring within these structures, such as rotation, expansion, transverse or swaying motions, modulation by waves and/or propagation of waves etc. Oscillations and waves have both been mainly addressed with simulations while observational evidence is rather rare. Even multi-wavelength observations give access to an only limited height span of their three-dimensional structure.

Shetye et al. (2019), using SST/CRISP spectral imaging and spectropolarimetric observations, found intensity variations with a period of 180 s, consistent with the typical 3 min acoustic oscillation in the chromosphere, and a decrease of the local oscillation periods from 180 s to 150 s that could be attributed to changes in the acoustic cavity dimension, a temperature increase, or additional magnetic effects. Their findings suggested the modulation of chromospheric oscillatory properties in the presence of swirls but indicated no respective wave excitation. Tziotziou et al. (2019) constructed two-dimensional power maps and derived dominant periods of oscillations, using a spectral analysis of SST/CRISP $\text{H}\alpha $ and Ca ii 8542 Å line profiles, $\text{H}\alpha $ Doppler velocity, and FWHM signals of a persistent 1.7 h vortex flow with significant substructure in the form of several intermittent chromospheric swirls. Significant oscillatory power has been found in the range of 3 to 5 min that peaks around 4 min and differs from the oscillatory behavior within a vortex-free quiet-Sun reference region. Their analysis also suggested the presence of two dominant motions, swaying (with periods of 200–220 s) and rotation. They found considerable oscillatory power for periods even up to 10 min in different heights, suggesting the existence of different MHD wave modes. Concerning rotation, the derived periods of $\sim 270~\text{s}$ for $\text{H}\alpha $ and $\sim 215~\text{s}$ for Ca ii 8542 Å, are both far shorter than previous estimates of $\sim 35~\text{min}$ obtained with IMaX/SUNRISE I observations by Bonet et al. (2010) but close to the peak values of swirling periods of $\sim 220~\text{s}$ at a height $z=90~\text{km}$ derived by Moll et al. (2011b) with near-surface solar convection simulations. Moreover, rotational periods seemed to increase with height in the atmosphere and decrease with radial distance from the centre of the vortex flow, implying deviations from a rigid rotation.

**Fig. 21:**
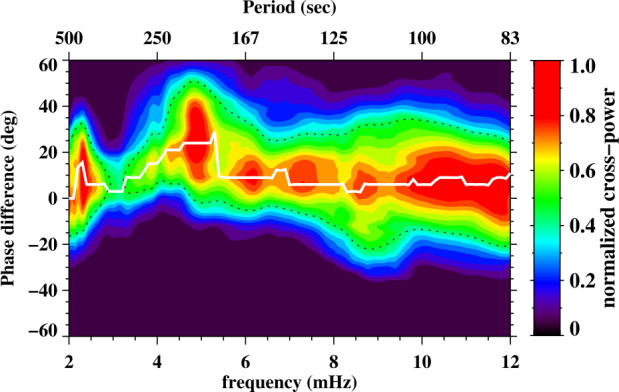
Phase differences between the $\text{H}\alpha \pm 0.26$ Å and $\text{H}\alpha $ line-centre intensity pair (known as halftone image, see Lites and Chipman 1979) within the vortex flow analysed by Tziotziou et al. (2020). Filled contours represent the cross-power distribution normalised to unity (vertical color bar) for each frequency element, white lines the position of maximum cross-power and black dashed contours indicate the 50% level of maximum cross-power that provides a measure of the scatter. The figure shows that a) dominant periods are found in the range of 3 to 5 min, b) phase differences are positive, indicative of upwards wave propagation, and c) the cut-off frequency (lowest frequency with non-zero phase difference that a wave mode can propagate) is much lower than the acoustic cut-off frequency of 5.2 mHz, suggesting the presence of MHD waves, identified as fast kink waves. Credit: adapted from Tziotziou et al. (2020), reproduced with permission ©ESO

Few observational reports of vortex-related wave signatures exist in the literature. Jess et al. (2009) have suggested the existence of torsional Alfvén waves, based on the analysis of $\text{H}\alpha $ FWHM oscillations within the magnetic field of a magnetic bright point. The analysis of multi-wavelength ROSA observations by Morton et al. (2013), complemented by relevant simulations, provided observational evidence that vortex motions of a strong photospheric magnetic flux concentration excite chromospheric torsional Alfvén and kink waves. From an analysis of Hinode/SOT and SST observations, Liu et al. (2019a) reported that prevalent intensity swirls in the solar photosphere can excite ubiquitous Alfvén pulses that propagate upwards into chromospheric layers. Liu et al. (2019b) further supported this claim by investigating the co-spatial and co-temporal rotation of photospheric velocity swirls and magnetic swirls found in photospheric simulations with the radiative MHD code Bifrost (Gudiksen et al. 2011). Recently, a wave analysis by Murabito et al. (2020) of IBIS Ca ii 8542 Å spectropolarimetric observations of two chromospheric rotating structures suggested that the combination of a slow actual rotation and a faster azimuthal phase speed pattern of a magnetoacoustic mode is responsible for the observed rotational vortex pattern. The most exhaustive observational work about waves in vortex flows has been initiated by the aforementioned meticulous oscillatory analysis by Tziotziou et al. (2019) of a persistent and complex vortex flow, suggesting the presence of various types of waves within it. Using a phase difference analysis of $\text{H}\alpha $ and Ca ii 8542 Å line profile observations and $\text{H}\alpha $ Doppler velocity and FWHM signals, Tziotziou et al. (2020) demonstrated the existence of Alfvénic type waves with phase speeds of $\sim 20\text{--}30~\text{km}\,\text{s}^{-1}$, with the dominant mode identified as fast kink waves that correspond to the observed swaying motion of 200–220 s period (see Fig. 21). They also provided observational evidence for localised Alfvénic torsional waves that are related to the dynamics of small and intermittent chromospheric swirls occurring within a larger and persistent vortex flow. Moreover, the existence of a standing wave pattern, that possibly arises from the interference of upwardly propagating kink waves with downwardly propagating (reflected at the transition region or the corona) kink waves, has been implied from an analysis of $\text{H}\alpha $ velocity-intensity phase difference maps. Shetye et al. (2019), in a similar spectral analysis of chromospheric swirls, did not provide any conclusive evidence that either the response to swirling photospheric motions or propagating Alfvénic waves can explain the observed oscillations.

### Simulations of Vortex-Related Waves and Energy Transfer

#### Acoustic Wave Excitation in Vortex Tubes

It is known that turbulent flows are a primary source of the observed oscillations in the photosphere. Numerical models support the observational evidence of near-surface acoustic sources to be located in the intergranular lanes or in their vicinity (Goode et al. 1992; Kumar 1994) and they point to an important class of acoustic sources associated with the interaction of turbulent vortex tubes. Swirling motions are naturally found in observations of the highly-turbulent solar magnetoconvection (see Sect. 7), as helical motions are a basic property of turbulent flows. Observations have shown that vortices are ubiquitous in the intergranular lanes of quiet-Sun regions (e.g. Pötzi and Brandt 2005; Bonet et al. 2008, 2010) and numerical simulations have indicated that they mainly correspond to nearly vertically-oriented vortex tubes (e.g. Brandenburg et al. 1996; Kitiashvili et al. 2011).

**Fig. 22:**
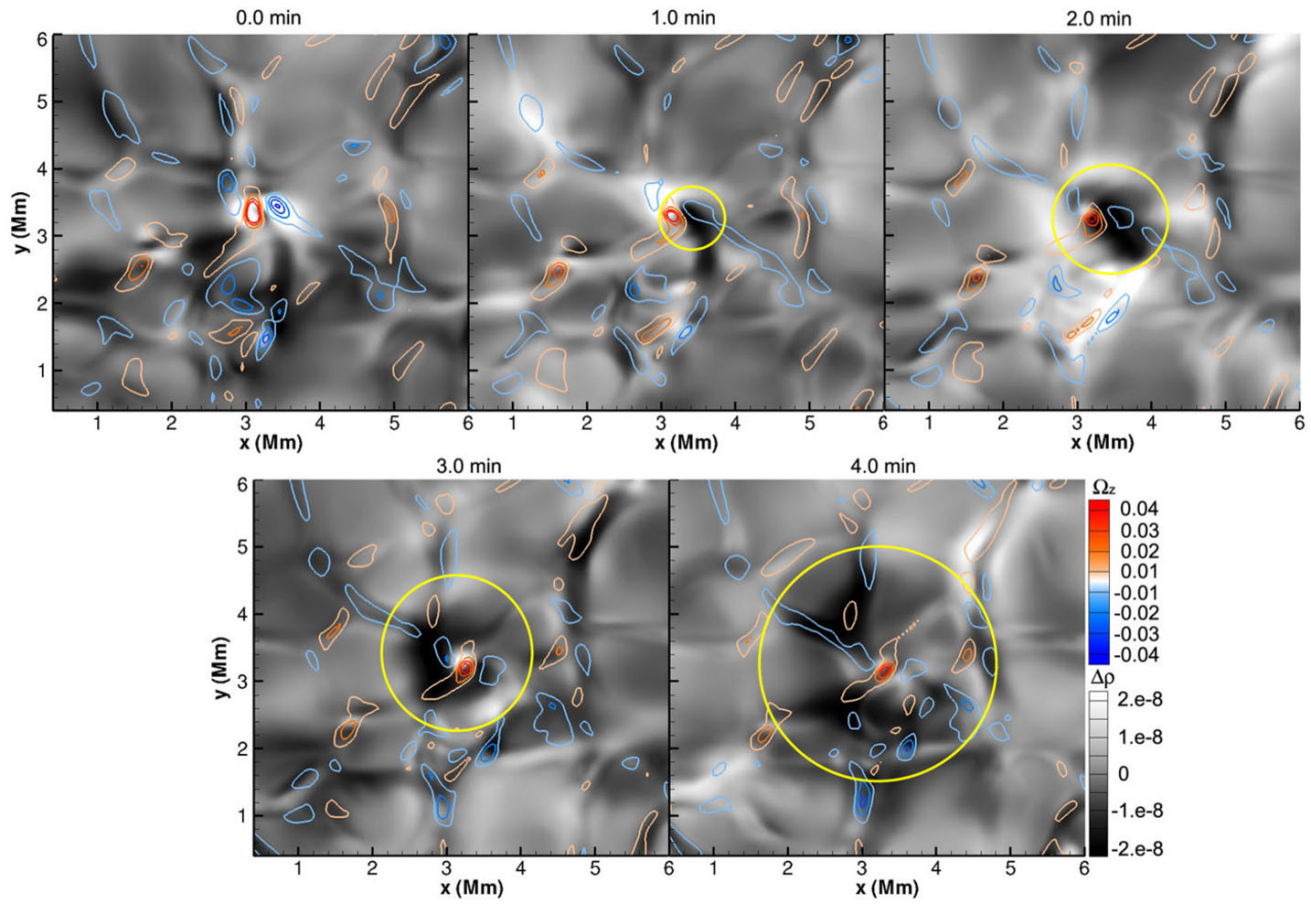
Temporal evolution of the density differences, calculated as $\rho (t_{i+1})-\rho (t_{i})$, at the solar surface with a cadence of 1 min. It shows acoustic wave excitation and radial propagation from a source, which arises from the interaction of two vortices of opposite rotation. Yellow circles indicate the wave front while red and blue contours correspond to the magnitude of the positive (clockwise) and negative (counterclockwise) vertical vorticity. Adapted from Kitiashvili et al. (2011) ©AAS. Reproduced with permission

The physical properties that describe vortices have been detailed in Sect. 7. As simulations have shown (Kitiashvili et al. 2011), vortices frequently interact with each other leading to the appearance of concentric waves that are excited approximately at the joint center of the vortex cores (see Fig. 22). Several factors, including the source structure itself, interference with waves from other sources, and the dynamics of surrounding flows define whether the wave fronts have the form of a circle or a sector. Figure 22 shows the acoustic wave excitation process in a sequence of density difference maps with a cadence of 1 min, in a horizontal slice corresponding to the solar surface of approximately optical depth unity. The approximate positions of the wave front are indicated by yellow circles. Although in simulations, this acoustic wave excitation process is very common, identifying individual excitation events is very difficult as the wave amplitude is of the same order of magnitude as the amplitude of acoustic waves emitted by other random acoustic sources, and the turbulent convection. Nevertheless, many excitation events with circular-shaped wave fronts, similar to the one shown in Fig. 22, are identified and examined. The wave speed is about $8\text{--}10~\text{km}\,\text{s}^{-1}$, but as explained in Kitiashvili et al. (2011), may vary because of possible background convective flows. In some cases, sequences of acoustic waves can be seen, when several wave fronts are excited with an interval of $\simeq 2\text{--}2.5~\text{min}$.

#### Vortex Motions and MHD Waves

**Fig. 23:**
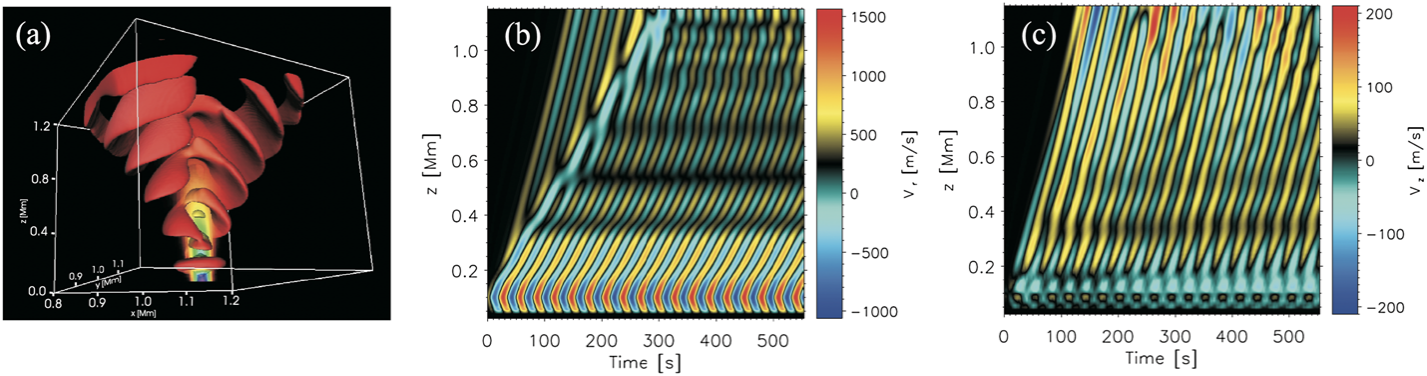
MHD wave generation by vortex motion, introduced at the base of the magnetic flux tube of a simulated solar atmosphere. (**a**) 3D rendering of the iso-surfaces of the horizontal velocity component, $v_{x}$, in the domain. (**b**) and (**c**) Time-distance diagrams of the radial ($v_{r}$) and the vertical ($v_{z}$) components of velocity, measured near the axis of the magnetic flux tube, respectively. Two types of waves with different phase speeds are measured from the time-distance diagrams: slow magneto-acoustic waves with the phase speed $V_{\mathrm{ph}}=3.8~\text{km}\,\text{s}^{-1}$, and fast magneto-acoustic waves with the phase speed $V_{\mathrm{ph}}=8.2~\text{km}\,\text{s}^{-1}$. From Fedun et al. (2011b)

Vortices can be expected to excite different Alfvénic type modes, such as kink waves, sausage modes, and torsional Alfvén waves (Fedun et al. 2011b,c; Shelyag et al. 2013; Verth et al. 2016). Likewise, twisting motions and photospheric horizontal and vertical footpoint motions of magnetic flux tubes have also been shown to trigger magneto-acoustic wave propagation (Fedun et al. 2011a). Figure 23 demonstrates such a process. In this simulation, a self-similar magnetic flux tube (see, e.g., Shelyag et al. 2009) is embedded in a hydrostatic solar-like atmosphere. A torsional driver is introduced at the base of the magnetic flux tube, which acts continuously. After some time, a stable pattern of magneto-hydrodynamic oscillations appears, which can be seen in Fig. 23(b, c), where both slow and fast magneto-acoustic oscillatory modes occur.

**Fig. 24:**
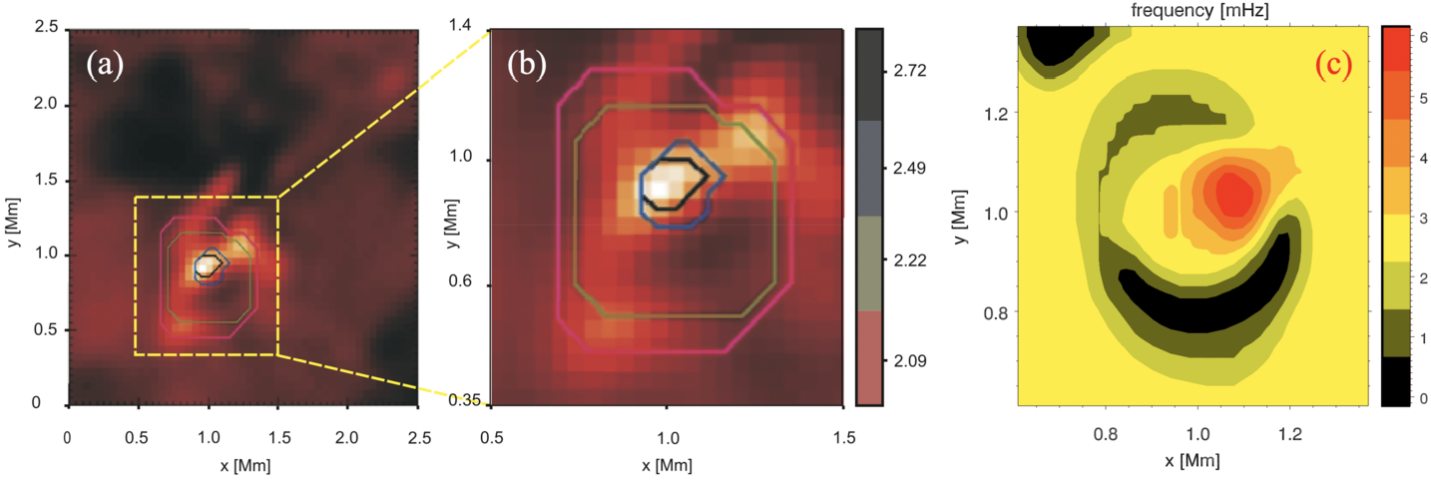
Comparison between the spatial distribution of the FWHM power frequency of $H_{\alpha}$ from the data set of Jess et al. (2009) and the spatial distribution of the Fourier transformed maximum power frequency of torsional Alfvén waves from the numerically simulated broadband vortex photospheric motion. From Fedun et al. (2011c) ©AAS. Reproduced with permission

Other simulations have addressed other aspects of the generation of waves by vortex flows. The process of acoustic waves excitation due to the interaction of vortices and their propagation (Kitiashvili et al. 2011) has been discussed in Sect. 8.2.1. Kitiashvili et al. (2013) showed that vortex-flows spontaneously triggered quasi-periodic mass eruptions (see Sect. 7.4) that can generate shocks and excite a wide variety of MHD modes and oscillations in the solar atmosphere, such as magnetoacoustic or Alfvén waves. Erdélyi and Fedun (2006, 2007) analytically investigated the generation of sausage waves both in incompressible and compressible magnetically twisted flux tubes. The excitation of different types of MHD wave modes within vortex flows, such as sausage, kink, and torsional Alfvén waves, has been simulated in an open flux tube with an analytically prescribed magnetic field structure by Fedun et al. (2011b) who used a high-frequency vortex motions as a driver at its footpoint. It was also demonstrated that the plasma structure can often act as a spatial frequency filter for torsional Alfvén waves within a magnetic flux tube that is driven by vortex motions (Fedun et al. 2011c). We note, that Jess et al. (2009) had already shown that torsional Alfvénic perturbations contribute to the non-thermal broadening of the $\text{H}\alpha $ line profile and the observed variations of its FWHM (see Fig. 24).

Musielak et al. (2007) have studied the propagation of linear torsional Alfvén waves along thin and isothermal magnetic flux tubes to demonstrate that the torsional wave propagation is not affected by any cutoff frequency. However, Musielak et al. (2010) came later to the conclusion that gradients in tube thickness and temperature can lead to cutoff frequencies for torsional tube waves. The question whether torsional tube waves are subject to a cutoff frequency or not is still not settled.

**Fig. 25:**
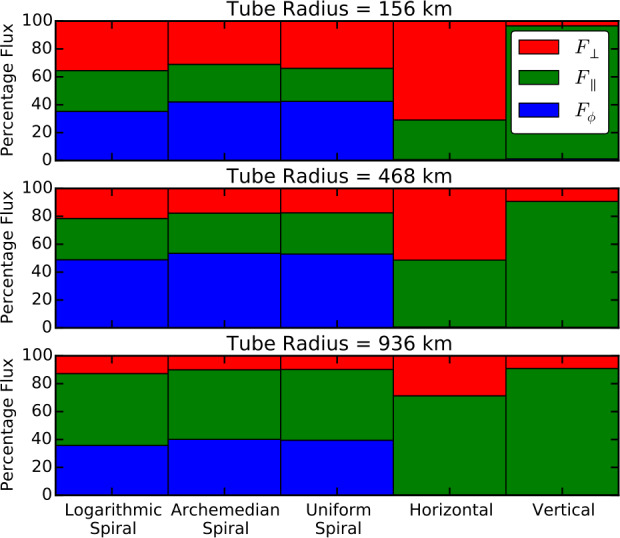
Energy-flux components in percentage of the total available energy flux for different drivers and flux surfaces. The $F_{\parallel}$, $F_{\perp}$, and $F_{\phi}$ components are respectively shown in green, red, and blue. The $F_{\parallel}$ component is generally the fast sausage mode. The $F_{\perp}$ component is almost exclusively excited by the slow kink mode and the $F_{\phi}$ component is attributed to the Alfvén mode. From Mumford et al. (2015) ©AAS. Reproduced with permission

Horizontal motions in magnetic vortices were identified as torsional Alfvénic perturbations by Shelyag et al. (2013) in MHD simulations that included a non-ideal equation of state and radiative transport. Slow and fast magnetoacoustic modes were reported in the vortex-like motion of a torsionally-driven flux tube (Vigeesh et al. 2012), while more recently Mumford et al. (2015) and Mumford and Erdélyi (2015) investigated the generation of Alfvén, torsional Alfvén, and other MHD waves (slow kink and slow or fast sausage modes), using several different photospheric drivers (such as Archimedean and logarithmic velocity spirals) in magnetic flux tubes. The authors calculated the energy flux components in percentage of the total available energy flux, for different simulated drivers at different distances from the magnetic flux tube axis. The components were taken parallel to the magnetic field, $\boldsymbol{n}_{\parallel}$, perpendicular to the magnetic flux surface, $\boldsymbol{n}_{\perp}$, and orthogonal to these, $\boldsymbol{n}_{\phi }= \boldsymbol{n}_{\parallel }\times \boldsymbol{n}_{ \perp}$, where $\boldsymbol{n}_{\parallel}$, $\boldsymbol{n}_{\perp}$, and $\boldsymbol{n}_{\phi}$ are unit vectors. The comparison between the parallel, $F_{\parallel}$, perpendicular, $F_{\perp}$, and azimuthal, $F_{\phi}$, components of the energy flux are shown in Fig. 25. For a more precise description of the flux components we refer to Vigeesh et al. (2012) and Khomenko and Cally (2012).

The excitation of Alfvén waves in short-lasting swirl events has also been investigated in Chmielewski et al. (2014) by numerically solving the ideal MHD equations with the FLASH code (Lee and Deane 2009; Lee 2013). Implementing an analytically derived model into this code, Murawski et al. (2015) simulated the propagation of torsional Alfvén and magnetoacoustic waves in a 3D twisted solar flux tube. These waves were excited by a localized pulse in the azimuthal velocity component launched at the top of the solar photosphere. Their propagation through the solar atmosphere up to the corona revealed a complex behaviour of twisted magnetic fields and flows. In a follow-up work, Murawski et al. (2018) performed 3D numerical simulations of impulsively generated swirls within an isolated flux tube and investigated the difference between launching the initial pulse at the centre of the flux tube and slightly off-centre. Two counter-rotating vortices appear inside the flux tube in the former case and a single one in the latter while fast magnetoacoustic kink waves and Alfvén waves (with $m = 1$ and $m = 0$, respectively) were generated within them.

More recently, Battaglia et al. (2021) investigated the evolution and origin of chromospheric swirls by analyzing numerical simulations with the CO^5^BOLD code and they introduced the new criterion of magnetic swirling strength that permitted the recognition of torsional perturbations in the magnetic field and their strong correlation with vortical motions. They found evidence for naturally emerging, upwardly propagating, unidirectional pulses of torsional Alfvén waves that are not oscillatory (see also Sect. 7.3 for further details).

#### Spectropolarimetric Diagnostics from Simulated Vortex Motions

Toroidal motions and vortex flows in the solar photosphere are nearly or fully incompressible motions (Shelyag et al. 2011a; Fedun et al. 2011b,c; Shelyag et al. 2012; Mumford et al. 2015), therefore causing little or non-observable photospheric continuum intensity variations or thermal variations of the shapes of photospheric absorption line profiles (Shelyag and Przybylski 2014). Line profile broadening has been suggested as a potential indicator of toroidal motions (see e.g. Van Doorsselaere et al. 2008; Jess et al. 2009; Bonet et al. 2010), however the transient nature of photospheric toroidal flows may prevent their detection through line broadening. On the other hand, using disk centre broadband observations it is possible to trace toroidal motions only on sufficiently large scales (Bonet et al. 2008). While disk centre high-resolution observations with new instruments, such as DKIST (Rast et al. 2021), potentially enables local correlation tracking techniques on scales of intergranular lanes, it is of interest to establish spectropolarimetric signatures of incompressible transient toroidal motions in the solar photosphere.

The geometry of plasma motions in photospheric magnetic flux concentrations prevents the detection of toroidal motions in Doppler shift at the solar disk centre due to absence of a LOS velocity component. However, according to the equation for the LOS velocity $v_{\mathrm{los}} = v_{z} \cos \theta + v_{h} \sin \theta $, where $\theta $ is the heliocentric angle with $\theta = 0$ corresponding to solar disk centre, the LOS velocity has a toroidal (horizontal) velocity component in off-disk centre observations. Furthermore, also the torsional magnetic field acquires a LOS component and becomes potentially visible in the Stokes-$V$ signal. Therefore, an attractive possibility is to establish the signatures of photospheric toroidal flows in spectropolarimetric observations off-disk centre.

**Fig. 26:**
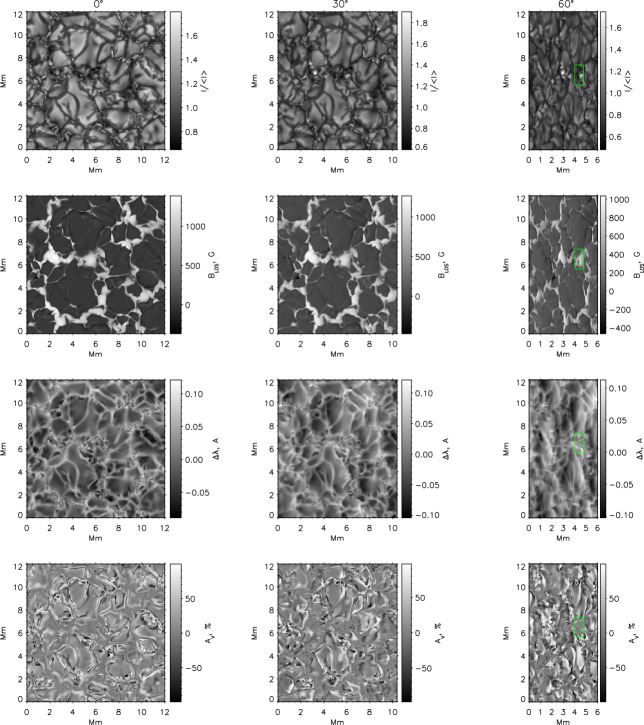
Radiative and magnetic field parameters computed for three different heliocentric angles of $\theta =0^{\circ}$, $30^{\circ}$, and $60^{\circ}$ for left, middle and right columns, respectively). Continuum intensity at 6300 Å (top row), LOS magnetic field at constant geometric level corresponding to the continuum formation height (second row), centre-of-gravity line Doppler shift (third row), and Stokes-$V$ amplitude asymmetry (bottom row) are calculated for the magnetically sensitive Fe i 6302 Å photospheric absorption line. The green rectangle in the $60^{\circ}$ plots indicate the position of a strong magnetic flux concentration. From Shelyag and Przybylski (2014) by permission of Oxford University Press on behalf of the Astronomical Society of Japan

Shelyag and Przybylski (2014) performed spectropolarimetric diagnostics of a 200 G unipolar model snapshot from the MURaM code at three different heliocentric angles ($\theta =0^{\circ}$, $30^{\circ}$, and $60^{\circ}$). Their results are given in Fig. 26, which shows the continuum intensity, the LOS magnetic field component, the Doppler shift $\varDelta _{\lambda}$, and the Stokes-$V$ profile asymmetry $A_{V}$ of the Fe i 6302 Å photospheric absorption line. As evident from the figure, toroidal motions in the magnetic flux concentrations are not visible for small observation angles (0 and $30^{\circ}$). However, at $60^{\circ}$, thin elongated grass-like structures in $\varDelta _{\lambda}$ and $A_{V}$ are identifiable within the green rectangle of Fig. 26 (see also the horizontal velocity, temperature, current density, and Doppler shift behaviour within this vortex structure shown in Fig. 2 of Shelyag and Przybylski 2014).

**Fig. 27:**
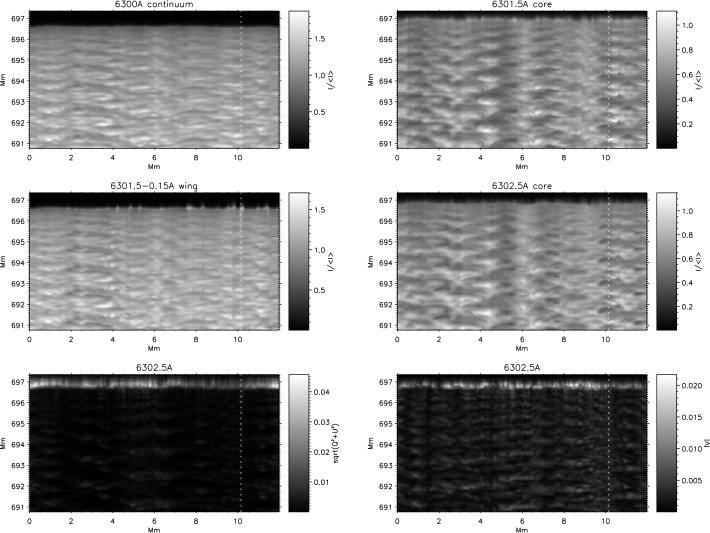
Synthetic spectropolarimetric diagnostics of solar limb observations in the spectral lines of Fe i 6301 Å and 6302 Å. Continuum, core and wing intensities (first two rows) and Stokes $\sqrt{Q^{2}+U^{2}}$ (bottom left) and Stokes-$V$ (bottom right) signals are shown for a simulated three-dimensional domain positioned at the curved solar limb. From Shelyag (2015) ©AAS. Reproduced with permission

The demonstrated dependence and aforementioned geometric considerations suggest that at the solar limb, torsional structures will be detectable with spectropolarimetry. Shelyag (2015) performed a simplified limb modelling based on the same 200 G unipolar MURaM model snapshot. The simulation fully mimicked the cylindrical curved solar limb and included the regions above and below the limb, therefore including absorption and weak emission regimes for the photospheric 6302 Å line. The radiation has been treated in one-dimensional approximation, and scattering as well as other multi-dimensional radiative transport effects, which are computationally prohibitively expensive, were not included. The line profile synthesis has been done in the LTE approximation. The results of this diagnostic procedure are shown in Fig. 27, which shows the simulated limb images of the 6300 Å continuum, the 6301.5 Å Fe i line core, the $6301.5-0.15$ Å line wing, the 6302.5 Å line core intensities, and the integrated linear Stokes signal $\sqrt{Q^{2}+U^{2}}$ and the Stokes-$V$ signal. Small-scale brightenings are easily identifiable in the $6301.5-0.15$ Å line wing images. Previously, such brightenings, together with a thin sub-arcsecond emission layer were seen in Hinode observations by Lites et al. (2010) and identified with light scattering. However, the modelling of Shelyag (2015) does not include light scattering, still it reproduces the brightenings as well as the emission layers. In this model, the brightenings are due to the Doppler-shifted limb emission of the 6301.5 Å line core. These Doppler shifts are caused by toroidal motions in magnetic field concentrations, and the emission cores are generated by the enhanced local temperature increase along the nearly horizontal ray, which crosses a localised temperature increase in the magnetic flux tubes, caused by lateral radiative heating.

Obviously, such modelling has to be treated with some caution as multiple important radiative transport effects are not included, and additional unphysical symmetries are artificially introduced due to computational limitations both in the MHD modelling part and in the radiative diagnostics. Nevertheless, the existence of a variety of features clearly linked to toroidal flows and oscillations strongly suggests further observational campaigns aiming at the solar limb with high cadence and high spatial resolution.

#### Energy Transfer in Vortex Motions

Vortex motions may be cardinal for the transfer of energy from the subsurface to the outer atmospheric layers of the Sun. Vortex-related MHD waves are considered a potential mechanism for this energy transport and the heating of the solar atmosphere with the estimated vertical Poynting flux providing a measure of electromagnetic energy flux streaming through it. Simulations by Shelyag et al. (2011a) suggested that vortex flows are the main source for a vertical Poynting flux sufficiently high to supply the necessary energy flux for the upper layers of the solar atmosphere. Wedemeyer-Böhm et al. (2012) estimated from simulations a net Poynting flux of $440~\text{Wm}^{-2}$ delivered by vortical flows in quite-Sun conditions at the transition region to the low corona. We note, that observational signatures of heating were found in IRIS Mg ii h/k spectral profiles of a chromospheric swirl co-observed with CRISP in the $\text{H}\alpha $ and Ca ii 8542 Å lines (Park et al. 2016). Estimations of energy fluxes related to Alfvén waves of up to a few hundred $\text{Wm}^{-2}$ in different layers of the solar atmosphere, were provided by Chmielewski et al. (2014) from numerical simulations. Also from simulations, Amari et al. (2015) estimated an upwardly directed Poynting flux of $\sim 300~\text{Wm}^{-2}$ at a height of 10 Mm, correlated with vortices and carried by upwardly propagating long living (30–50 min) Alfvén waves. Murawski et al. (2018) derived from their simulations energy fluxes of Alfvén waves within magnetic swirls of $\sim 10^{4}~\text{Wm}^{-2}$ in the solar chromosphere and $\sim 10^{2}~\text{Wm}^{-2}$ in the inner corona, sufficient to balance the corresponding energy losses at those heights, but they cannot account for the observed temperatures in the corona (as waves probably dissipate their energy earlier in the chromosphere). Using MHD simulations of swirls with the MURaM code, Yadav et al. (2020, 2021) suggested that most of the energy is carried by small-scale horizontal motions or even smaller vortices, therefore energy fluxes derived from low-resolution data may largely underestimate the true energy flux. They estimated Poynting flux densities related to vortices equal to 33.5, 12.5 and $7.5~\text{kWm}^{-2}$ at heights of 500, 1000 and 1500 km, respectively, above the solar surface. Finally, Battaglia et al. (2021) estimated from CO^5^BOLD simulations a mean upwardly directed Poynting flux due to chromospheric swirls of $12.8 \pm 6.5~\text{kWm}^{-2}$ at the base of the chromosphere. This flux is mostly due to superposition of small-scale swirls originating from network like magnetic patches.

**Fig. 28:**
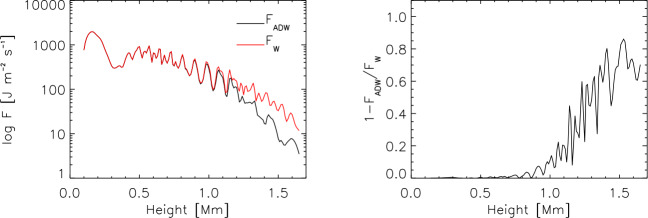
Poynting-flux absorption due to ambipolar diffusion in a magnetic flux tube embedded in the solar atmosphere. Left panel: comparison of average Poynting fluxes with ($ADW$) and without ($W$) absorption mechanism due to ambipolar diffusion. Right panel: absorption coefficient calculated from the simulation. From Shelyag et al. (2016) ©AAS. Reproduced with permission

In a theoretical work, Soler et al. (2017) investigated the reflection, transmission, and dissipation of propagating torsional Alfvén waves in expanding magnetic flux tubes and they showed that such waves of intermediate frequencies are efficiently transmitted to the corona while high-frequency waves are completely damped in the chromosphere. We note, however, that the proper estimation of the amount of upwardly propagating waves that reach the corona, and waves that are partially/entirely dissipated below it, and the way in which their dissipation takes place, are still largely unknown. Turbulence, produced through interaction of counter-propagating waves, could also play a role in the heating of the chromosphere. Furthermore, part of the wave energy transmitted across the transition region could also produce turbulence in the corona (Matthaeus et al. 1999; van Ballegooijen et al. 2011). It has also been suggested that ambipolar diffusion can be responsible for the absorption of waves in magnetic flux tubes of the solar chromosphere (Khomenko and Collados 2012). Shelyag et al. (2016), using a simplified model of a solar magnetic field concentration embedded in the solar photosphere and chromosphere, have demonstrated that ambipolar diffusion can provide sufficient absorption for maintaining the thermal structure of the solar atmosphere. In Fig. 28, the height dependence of the Poynting flux is compared for simulations with ($ADW$) and without ($W$) ambipolar diffusion. The right panel of the figure shows the absorption coefficient. Evidently, it exhibits a strong increase in the middle and upper chromospheric layers in the case of ambipolar diffusion. Further attention to ambipolar diffusion and other effects of non-ideal MHD is given in Sect. 9.

As a final account on the energetics of vortices, we note that photospheric swirling motions have consequences for the magnetic field such as twisting it and forming a helical structure with the potential of storing significant amounts of magnetic energy. Once, however, the twist exceeds a threshold value (see, e.g., Steiner et al. 1986), the system can undergo a kink instability that can lead to the release of large amounts of magnetic energy (Rappazzo et al. 2013). Rappazzo et al. (2019) numerically investigated a scenario of multiple velocity vortex drivers, co-rotating and counter-rotating with respect to each other, at the base of magnetic flux tubes in the photosphere. They found that for co-rotating vortices, the photospheric vortices generate an anti-parallel magnetic field component in the two flux tubes, leading to magnetic reconnection. Once reconnection merges the two twisted fields, the resulting dynamics are very similar to the case for a single photospheric vortex, namely that an inverse cascade of magnetic energy is established.

## Influence of Effects of a Non-ideal Plasma on Vortices

In this section we treat effects of a non-ideal solar plasma and their influence on vortices.

### Effects of a Non-ideal Plasma on Vortex Dynamics

The plasma in the lower solar atmosphere (photosphere and chromosphere) contains a large fraction of neutrals. The neutral fraction reaches 0.999 in the cooler photospheric layers around the temperature minimum, and around 0.5 in the chromosphere (here, 0 means fully ionized plasma, and 1 means neutral gas, see Ballester et al. 2018). The presence of neutrals makes the plasma properties different from that of an ideal plasma, and introduces several new effects. The effects frequently mentioned in the context of partially ionized solar plasma are the ambipolar diffusion, the modified Hall effect, and the Biermann battery effect. From these three, only the first one is directly related to the presence of neutrals. Mathematically, these non-ideal effects manifest themselves as additional terms in the generalized Ohm’s law. In the photosphere and the bottom part of the chromosphere, the so called single-fluid approximation can be applied, under which neutrals and plasma are treated as a single fluid with unique velocity, mass density, and temperature, describing both neutrals and ions. Then, the generalized Ohm’s law takes the following form (Khomenko et al. 2014):

31 $$\begin{aligned}{} [\boldsymbol{E}+ \boldsymbol{v}\times \boldsymbol{B}] =\eta \boldsymbol{j} - \frac{\mathbf{\nabla}p_{e}}{e\, n_{e}} +\eta _{H} \frac{(\boldsymbol{j} \times \boldsymbol{B})}{|\boldsymbol{B}|} - \eta _{A} \frac{\left (\boldsymbol{j} \times \boldsymbol{B} \right ) \times \boldsymbol{B} }{|\boldsymbol{B}|^{2}} , \end{aligned}$$

where $n_{e}$ is the electron number density, $e$ the electron charge, and $p_{e}$ the electron pressure. The non-ideal terms on the right hand side are the Ohmic term, the Biermann battery term, the Hall term, and the ambipolar term. The ambipolar diffusion coefficient, $\eta _{A}$, and the Hall coefficient, $\eta _{H}$, are given by the expressions

32 $$ \eta _{A} =\frac{\xi _{n}^{2}|B|^{2}}{\alpha _{n}}; \qquad \eta _{H}= \frac{|B|}{en_{e}}. $$

In these expressions, the units of both quantities are $[ml^{3}/tq^{2}]$. By $\xi _{n}=\rho _{n}/\rho $ we denote the neutral fraction, and by $\alpha _{n}$ we denote the collisional parameter. The latter takes into account collisions between electrons and ions, and ions and neutrals (see Khomenko et al. 2014, 2018; Ballester et al. 2018).

The generalized induction equation (obtained by introducing Eq. (31) into Faraday’s law, $\partial \boldsymbol{B}/\partial{t}=-\boldsymbol{\nabla}\times \boldsymbol{E}$), and the vorticity equation, Eq. (8), can be operated to come into similar forms. The induction equation becomes

33 $$\begin{aligned} \frac{\partial \boldsymbol{B}}{\partial t} = \boldsymbol{\nabla}\times ( \boldsymbol{v}\times \boldsymbol{B}) + \frac{\boldsymbol{\nabla}p \times \boldsymbol{\nabla}\rho}{\rho ^{2}} \frac{\mu m_{p}}{e}- \boldsymbol{\nabla}\times \left [ \frac{\boldsymbol{j}\times \boldsymbol{B}}{\rho}\right ] \frac{\mu _{i} m_{p}}{e\xi _{i}} + \boldsymbol{\nabla}\times (\eta _{A} \boldsymbol{j_{\perp}}). \end{aligned}$$

In this equation we denote the mean molecular weight by $\mu $, and the mean molecular weight corresponding to the ionized fluid alone by $\mu _{i}$, while $m_{p}$ is the proton mass. In the derivation of this induction equation we have assumed that the ion fraction $\xi _{i}=\rho _{i}/\rho $, $\mu $, and $\mu _{i}$ are constant in space. We have defined the current perpendicular to the magnetic field, $\boldsymbol{j}_{\perp} = [(\boldsymbol{j} \times \boldsymbol{B})]\times \boldsymbol{B}/|\boldsymbol{B}|^{2}$. Here and below, we have omitted the Ohmic term because it is irrelevant for the discussion: the Ohmic diffusion coefficient is orders of magnitude smaller than the ambipolar diffusion coefficient in the chromosphere of the Sun (Khomenko and Collados 2012).

The comparison of Eq. (8) with Eq. (33) reveals the presence of the source terms proportional to $\boldsymbol{\nabla}p \times \boldsymbol{\nabla}\rho $ in both equations (baroclinic terms). These terms are zero for a barotropic fluid, i.e., a fluid where the gradients of pressure and density are parallel to each other, as, for example is the case for an isothermal fluid. Otherwise, these source terms will be non-zero. The similarity between the baroclinic terms in both equations was used as an argument to suggest that a magnetic field can be generated by means of vorticity, even if no magnetic field exists initially in the medium. This effect was first investigated by Biermann (1950). The Biermann battery effect was suggested to provide seeds for galactic dynamos (Kulsrud and Zweibel 2008). It was also proposed to seed the magnetic field in the case of the solar small-scale dynamo (Khomenko et al. 2017).

There is a simple order of magnitude relation between the magnetic field and vorticity that can be used to evaluate the magnitude of the magnetic field produced by the battery in a unit time,

34 $$ \frac{e |\boldsymbol{B}|}{\mu m_{p}}\sim |\boldsymbol{\omega}| . $$

**Fig. 29:**
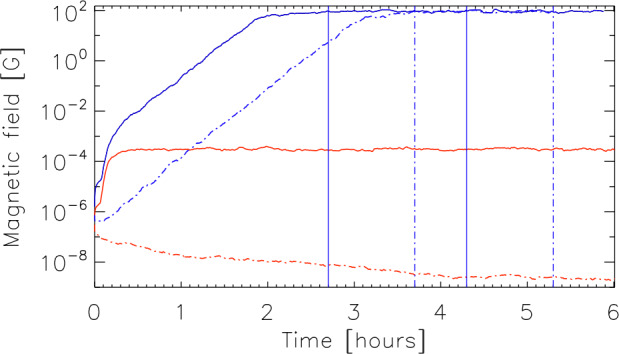
Spatially-averaged modulus of the magnetic field at the solar surface (optical depth $\tau _{5}=1$) as a function of time after the start of the action of the battery effect (solid curves) or after the introduction of the random seed (dashed curves) in the simulations of the small scale solar dynamo. Solid blue: battery seed; dashed blue: random seed; solid red: battery seed with higher numerical diffusion; dashed red: random seed with higher numerical diffusion. Credit: Khomenko et al. (2017), reproduced with permission ©ESO

For the typical values of velocities and scales at the solar surface ($\sim 10~\text{km}\,\text{s}^{-1}$, 1 Mm), an order of magnitude estimate gives $B$ of about $10^{-6}~\text{G}$. Once generated by the battery effect, the magnetic field can be amplified via the small scale solar dynamo action. The numerical simulations of a battery-seeded small scale solar dynamo by Khomenko et al. (2017) confirm the magnitude of the generated field. They have shown that the amplification is achieved very efficiently since the initial field amplitude and spatial structure are in agreement with the granulation pattern, unlike for the randomly seeded field (Rempel 2014; Kitiashvili et al. 2015). This is illustrated in Fig. 29, which shows that the saturated dynamo regime is achieved within a time period of only 2 hours when seeded with the battery field. Per se, the fields generated through the battery are non-important and way too weak to be detected. Nevertheless, one can speculate that, even if plasma emerges to the surface with extremely weak magnetic field, it will be re-populated within about an hour by close to saturated, small-scale dynamo fields thanks to the initial action of the battery effect.

By comparing the vorticity equation, Eq. (8) with the induction equation, Eq. (33), it can be noted that the Lorentz force-related term in the former is analogous to the Hall effect-related term in the latter. However, in the case of partially ionized plasma, the Lorentz force-related term is multiplied by the inverse of the ion fraction, $\xi _{i}^{-1}$. This fraction is a number typically much smaller than unity in the photosphere and the chromosphere of the Sun. Therefore, it has been concluded that the Hall effect operates at frequencies that are a factor of $\xi _{i}^{-1}$ lower compared to the case of the fully ionized plasma (Pandey and Wardle 2008; Pandey et al. 2008; Cheung and Cameron 2012; Cally and Khomenko 2015). A measure for the importance of the Hall effect is given by the so-called Hall coefficient $\epsilon =\omega /(\omega _{ci}\xi _{i})$, which relates the wave frequency to the ion-cyclotron frequency, and is scaled with $\xi _{i}^{-1}$ (Cally and Khomenko 2015). Estimates show that, for the solar case, this scaling can increase the typical time scales of the Hall effects to values as large as $10^{-2}$–$10^{-3}$ seconds. While still not resolved in observations, these time scales are close to the typical integration time scales in numerical simulations.

According to the discussion provided after Eq. (7), most of the vorticity in realistic simulations of the solar atmosphere in regions where the dynamics are dominated by the magnetic field is generated through the term containing the curl of the magnetic tension force (Shelyag et al. 2011b; Canivete Cuissa and Steiner 2020). In the same vein, recent studies show that Alfvén waves can be generated in the partially ionized solar atmosphere through the action of the Hall effect (Cally and Khomenko 2015; González-Morales et al. 2019; Raboonik and Cally 2019). The restoring force of Alfvén waves is the magnetic tension, thus, the generation of vorticity and the generation of Alfvén waves are directly related to each other. Alfvén waves in the solar atmosphere can be produced through the so-called Hall-induced mode transformation, since the out-of-plane Hall current couples magnetic fast-mode waves and Alfvén waves in the low-$\beta $ plasma. For this process to work efficiently, the fast-mode magnetic waves need to be created through the primary mode transformation from the fast-mode acoustic waves ($p$-modes), happening at heights where plasma-$\beta $ is around unity. Further up, these fast magnetic modes will couple to the Alfvén modes. The efficiency of this coupling depends on the Hall parameter $\epsilon $. Therefore, the process works the best when the maximum of $\epsilon $ is reached in the low-$\beta $ atmosphere.

**Fig. 30:**
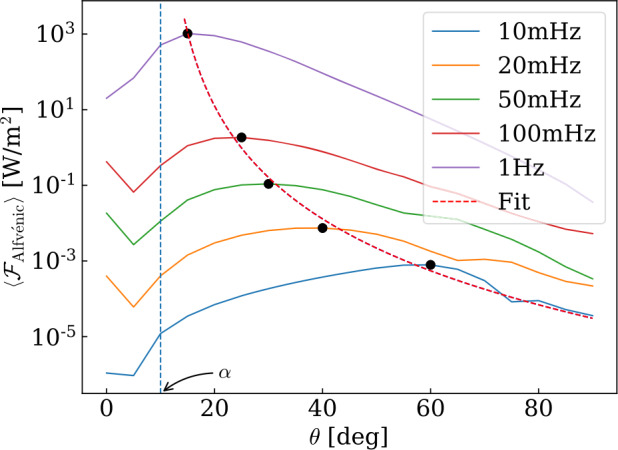
Time-averaged magnetic Poynting flux due to Alfvén waves at height 450 km as a function of magnetic field inclination with respect to the vertical direction, $\theta $, in idealized simulations of the Hall-induced Alfvén wave production. Solid colored lines are for waves with different frequencies, their maxima are marked with black dots. Notice the displacement of the maxima towards larger angles for decreasing frequencies. The dotted vertical line marks the inclination of the wave vector with respect to the vertical. From González-Morales et al. (2019) ©AAS. Reproduced with permission

González-Morales et al. (2019) have performed numerical simulations of the entire chain of mode transformations, including the Hall-induced one, and have shown that it efficiently generates Alfvén waves of 0.1–1 Hz frequency at chromospheric heights. The energy of these waves might be comparable to the chromospheric energy losses by radiation. An illustration of this process is provided with Fig. 30. It shows the magnetic energy flux contained in the Alfvén waves generated through the Hall effect in the upper photosphere as a function of the inclination angle $\theta $ of the magnetic field and for different wave frequencies. The flux exponentially increases with frequency and is largest for vertical magnetic fields. Realistic simulations of solar magneto-convection including the Hall effect are scarce, and will be described in Sect. 9.2.

The ambipolar diffusion term is only present in the induction equation, but not in the vorticity equation. This term is a dissipative term and allows to extract the energy from the magnetic field and to convert it into heat (Khomenko and Collados 2012; Martínez-Sykora et al. 2012). This can be seen by operating the total energy equation and the internal energy equation, and also considering the Poynting flux. The evolution of the total energy $e_{\mathrm{tot}}=\rho v^{2}/2 + e_{\mathrm{int}} + B^{2}/(2\mu _{0})$ is described by the following equation,

35 $$ \frac{\partial e_{\mathrm{tot}}}{\partial t} + \nabla \boldsymbol{S} = \rho \boldsymbol{v}\boldsymbol{g}, $$

with the energy flux given by

36 $$ \boldsymbol{S}=\left ( \frac{\rho v^{2}}{2} + p + e_{ \mathrm{int}} \right ) \boldsymbol{v} + \frac{\boldsymbol{E}\times \boldsymbol{B}}{\mu _{0}} + \boldsymbol{q} + \boldsymbol{F}_{R}. $$

Here, $\boldsymbol{q}$ is the heat flux, and $\boldsymbol{F}_{R}$ is the radiative energy flux. The electro-magnetic energy flux $\boldsymbol{E}\times \boldsymbol{B}/{\mu _{0}} $ is obtained via the generalized Ohm’s law,

37 $$ \boldsymbol{S}_{\mathrm{EM}}= \frac{\boldsymbol{E}\times \boldsymbol{B}}{\mu _{0}}=- \frac{(\boldsymbol{v} \times \boldsymbol{B}) \times \boldsymbol{B}}{\mu _{0}} + \eta _{H} \frac{|B|\boldsymbol{j}_{\perp}}{\mu _{0}}- \frac{\nabla p_{\mathrm{e}} \times \boldsymbol{B}}{en_{e}\mu _{0}} - \frac{\boldsymbol{B} \times \left (\eta _{A} \boldsymbol{j}_{\perp }\right )}{\mu _{0}}. $$

The first term on the right hand side is the advective term, and it can be expanded as $-[\boldsymbol{v} \times \boldsymbol{B}]\times \boldsymbol{B}= | \boldsymbol{B}|^{2}\boldsymbol{v} - (\boldsymbol{B}\boldsymbol{v}) \boldsymbol{B}$. The rest of the terms either redistribute the total energy (Hall and battery), or dissipate it (ambipolar). Considering the total energy in the volume $V$, $e_{\mathrm{tot}}$, the divergence theorem tells us that its time evolution is related to the energy flux as,

38 $$ \frac{\partial }{\partial t}\int _{V} e_{ \mathrm{tot}}\,{\mathrm{d}}V= - \boldsymbol{\nabla}\boldsymbol{S}{\mathrm{d}}V=- \int _{s} \boldsymbol{S}\,{\mathrm{d}} \boldsymbol{s}\,. $$

Then, the total energy is conserved if there is no flux across the boundaries of the domain. For an ideal plasma, the expression for the electro-magnetic flux simplifies to

39 $$ \boldsymbol{S}_{\mathrm{EM}}^{\mathrm{ideal}}= \frac{\boldsymbol{E}\times \boldsymbol{B}}{\mu _{0}}=- \frac{(\boldsymbol{v} \times \boldsymbol{B}) \times \boldsymbol{B}}{\mu _{0}}. $$

With the non-ideal terms present in Eq. (37), the magnitude of $\boldsymbol{S}_{\mathrm{EM}}^{\mathrm{ideal}}$ will vary as a consequence of the conservation of the total flux, $\boldsymbol{S}_{\mathrm{EM}}$. The influence of the ambipolar diffusion into the energy conservation is best seen from the internal energy equation,

40 $$ \frac{\partial e_{\mathrm{int}}}{\partial t} + \boldsymbol{\nabla}( e_{ \mathrm{int}} \boldsymbol{v} + \boldsymbol{q} + \boldsymbol{F}_{R}) + (p \boldsymbol{\nabla}) \boldsymbol{v} = \boldsymbol{j} [\boldsymbol{E} + \boldsymbol{v} \times \boldsymbol{B}] , $$

where the right hand side contains the work of electromagnetic forces $( \boldsymbol{j} [\boldsymbol{E} + \boldsymbol{v} \times \boldsymbol{B}] ) \approx \eta _{A}\boldsymbol{j_{\perp}}^{2}$ (the contribution of the battery term can be safely neglected). The ambipolar diffusion allows to dissipate magnetic energy and convert it into thermal energy by dissipating currents that flow perpendicular to the magnetic field. At the same time, this dissipative effect will cause the removal of some fraction of the ideal Poynting flux, $\boldsymbol{S}_{\mathrm{EM}}^{\mathrm{ideal}}$, in the plasma volume. The currents perpendicular to the magnetic field can be associated to the perturbations caused by waves with transverse velocities, i.e., to Alfvén waves. Therefore, the ambipolar effect must allow for an efficient dissipation of Alfvén waves. This should also affect vorticity through the formal relation between the magnetic field and the vorticity. Shelyag et al. (2016) performed 3D idealized numerical simulations of the Alfvén wave propagation and dissipation in a solar flux tube. This work confirmed that locations with strong transverse velocity and currents are associated with locations of enhanced temperatures. Effective absorption of Poynting flux of up to 80% was observed in these simulations. The effect of the ambipolar diffusion is more propounded in the upper chromosphere since the value of $\eta _{A}$ is maximum there.

### Effects of a Non-ideal Plasma in Realistic Simulations

There are only few, so called, realistic simulations, in which effects of a non-ideal plasma due to partial ionization of the solar plasma have been taken into account (Martínez-Sykora et al. 2012; Cheung and Cameron 2012; Martínez-Sykora et al. 2017, 2016; Khomenko et al. 2017, 2018; Martínez-Sykora et al. 2020; Nóbrega-Siverio et al. 2020). These simulations have mainly focussed on the effect of ambipolar diffusion on chromospheric heating, shock waves, flux emergence, or chromospheric structure formation. From the models cited above, only those of Khomenko et al. (2017, 2018) and González-Morales et al. (2020) were performed in 3D, which is indispensable to study vorticity. None of the simulations mentioned above specifically addresses the questions of generation and dissipation of vorticity and the influence of partial ionization effects on it. This is still a field to be developed.

The behavior of Alfvén waves and the vorticity are intimately related. Khomenko et al. (2018) and González-Morales et al. (2020) attempted to address the question of dissipation of Alfvén waves thanks to the ambipolar diffusion effect in their realistic 3D simulations of the small scale solar dynamo and magneto-convection with implanted vertical field. Their simulations reach mid-chromospheric heights, of some 1400 km above the continuum formation level. González-Morales et al. (2020) completed the study of Khomenko et al. (2018) by including the Hall effect. González-Morales et al. (2020) analyzed three simulation runs of 2 solar hours duration each, all of them developed from saturated battery-seeded dynamo from Khomenko et al. (2018). The first series only had the battery effect included; the second one had additionally the ambipolar diffusion included; and the third one with both ambipolar diffusion and the Hall effect included. Since the simulations run for a long time, the simulation snapshots cannot be compared one to one because by then, the convective pattern is different, but a statistical comparison can still be performed.

**Fig. 31:**
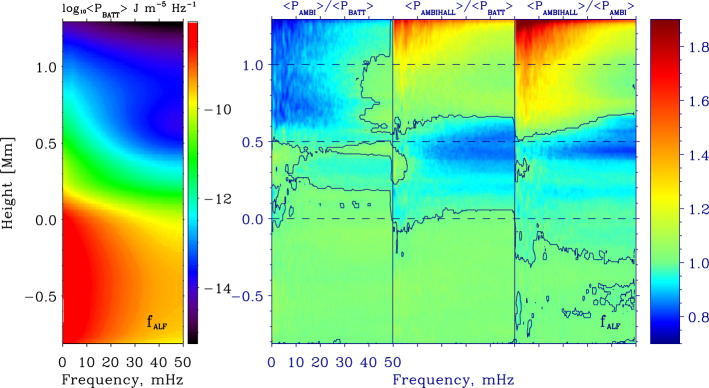
Left panel: $\log _{10}$ of power of the field-aligned Alfvénic fluctuations, $f_{\mathrm{alf}}$, defined in Eq. (41), as a function of height (vertical axis) and frequency (horizontal axis) computed from the battery excited small scale dynamo simulation of González-Morales et al. (2020). Right panels: power ratio $\langle P \rangle _{\mathrm{AMBI}}/\langle P \rangle _{\mathrm{BATT}}$, $\langle P \rangle _{\mathrm{AMBIHALL}}/\langle P \rangle _{ \mathrm{BATT}}$, and $\langle P \rangle _{\mathrm{AMBIHALL}}/\langle P \rangle _{ \mathrm{AMBI}}$ as a function of height and frequency. Black contour lines follow the locations of the power ratio equal to one. Credit: adapted from González-Morales et al. (2020), reproduced with permission ©ESO

In order to study the Alfvén waves in their simulations, González-Morales et al. (2020) followed the strategy of Cally (2017), using the argument about their incompressibility to construct the quantity

41 $$ f_{\mathrm{alf}} = \hat{\boldsymbol{e}}_{\parallel }\cdot \nabla \times \boldsymbol{v}, $$

where $\hat{\boldsymbol{e}}_{\parallel}$ is the unit vector aligned to the magnetic field. This quantity, $f_{\mathrm{alf}}$, describes an incompressible field-aligned perturbation, characteristic for Alfvén waves in regions of both low and high plasma-$\beta $. Note that $f_{\mathrm{alf}}$ would be zero for a pure kink mode in 2D slab geometry. Also, given the complex and dynamic nature of the simulated magnetic structure, it is very hard to separate individual pure wave modes when constructing the quantity $f_{\mathrm{alf}}$. González-Morales et al. (2020) constructed horizontally averaged power spectra of $f_{\mathrm{alf}}$ as a function of frequency and height, and compared these maps for the three simulation runs labeled as “BATT” (for pure MHD), “AMBI” (when ambipolar diffusion is present), and “AMBIHALL” (when both ambipolar & Hall are present). Figure 31 shows the comparison.

The power ratio $\langle P \rangle _{\mathrm{AMBI}}/\langle P \rangle _{\mathrm{BATT}}$ of the quantity $f_{\mathrm{alf}}$ on Fig. 31, demonstrates that, depending on the frequency, there can be an up to 20–30% decrease in the power of the Alfvénic fluctuations at heights above 600 km, which can be interpreted as an “absorption” or dissipation of the Alfvén waves due to the effect of ambipolar diffusion. Notice that ambipolar dissipation acts at rather low heights in their 3D dynamo simulations, where the plasma is still hot and dense. This physical situation is quite different from the one described in the 2D magnetic flux emergence models by Nóbrega-Siverio et al. (2020). The latter work finds that non-equilibrium ionization of Hydrogen affects the neutral fraction in the cold, rarefied bubbles that are produced by nearly adiabatic plasma expansion, leading to a higher ionization fraction that would correspond to these low temperatures under LTE conditions. Unlike that, the ambipolar heating in the model by González-Morales et al. (2020) does not occur in cold and rarefied areas, but rather it happens near the magnetic canopies. According to calculations provided in Khomenko et al. (2018, their Fig. 11), the extra energy released by ambipolar dissipation in these heights is mostly spent on increasing the plasma temperature rather than on Hydrogen ionization. Therefore, LTE computation of the ionization fraction in the models by González-Morales et al. (2020) is a good approximation and does not influence the conclusions presented in Fig. 31.

Figure 31 (second of the right panels) also shows that the corresponding ratio of $\langle P \rangle _{\mathrm{AMBIHALL}}$ and $\langle P \rangle _{\mathrm{BATT}}$ has an excess of about 40–60% at heights above 1 Mm, which is attributed to the Hall effect. The latter is confirmed by analyzing the rightmost panel of Fig. 31, which compares the power maps of $\langle P \rangle _{\mathrm{AMBIHALL}}$ and $\langle P \rangle _{\mathrm{AMBI}}$, which differs from the middle panel only by the presence of the Hall effect. The power of $f_{\mathrm{alf}}$ is about a factor 2 larger in the simulations with the Hall effect at the top of the domain.

The excess of incompressible Alfvénic perturbation due to the Hall effect in González-Morales et al. (2020) is particularly interesting, but it needs to be further explored with simulations of different magnetic field configurations. We mention two other works, Martínez-Sykora et al. (2012) and Cheung and Cameron (2012), where the Hall effect has been included in 2.5D magneto-convection simulations. In 2.5D simulations of magneto-convection under conditions of a sunspot umbra, Cheung and Cameron (2012) revealed the presence of a small out-of-plane component of the magnetic field (about 5 G strength) and velocity ($\sim 100~\text{m}\,\text{s}^{-1}$), as a consequence of the Hall effect. This component does not appear in the absence of the Hall effect. It should be noted, however, that the Hall effect is intrinsically 3D, and modelling its action is better achieved in complete 3D setups.

The Hall effect could, in principle, help increasing Alfvénic wave power in the upper layers of the solar atmosphere thanks to the mechanism of fast to Alfvén mode conversion described in Sect. 9.1. Another possibility is that the Hall effect indirectly helps bringing Alfvén waves higher up by modifying the magnetic structure of the atmosphere. Khodachenko and Zaitsev (2002) suggested the formation of strong flux tubes thanks to the Hall effect. This mechanism works in locations with converging flow of partially ionized plasma. In the solar upper photosphere and low chromosphere, the situation is such that the electrons are bound to the magnetic field while the ions are dragged by neutrals through collisions. Therefore, a charge separation occurs. This charge separation creates an electric current that is able to intensify the magnetic field. The current produced by this mechanism reaches its limiting values when the ions and neutrals flow with the same speed. In the absence of any other additional force, the balance is to be achieved between the Lorentz force and the collisional force, created by the relative motion of ions and neutrals.

42 $$ \boldsymbol{j}\times \boldsymbol{B}=m_{\mathrm{i}} n_{ \mathrm{i}} \nu _{\mathrm{in}}( \boldsymbol{v}_{\mathrm{i}}- \boldsymbol{v}_{\mathrm{n}}), $$

where $\boldsymbol{v}_{\{{\mathrm{i},\mathrm{n}}\}}$ is, respectively, the ion or neutral velocity. This balance limits the field strength that is possible to be created through this mechanism. The intensification is likely to begin in the upper layers and propagates downwards. It is not yet clear if the mechanism of Khodachenko and Zaitsev (2002) is present in simulations: it requires further exploration.

**Fig. 32:**
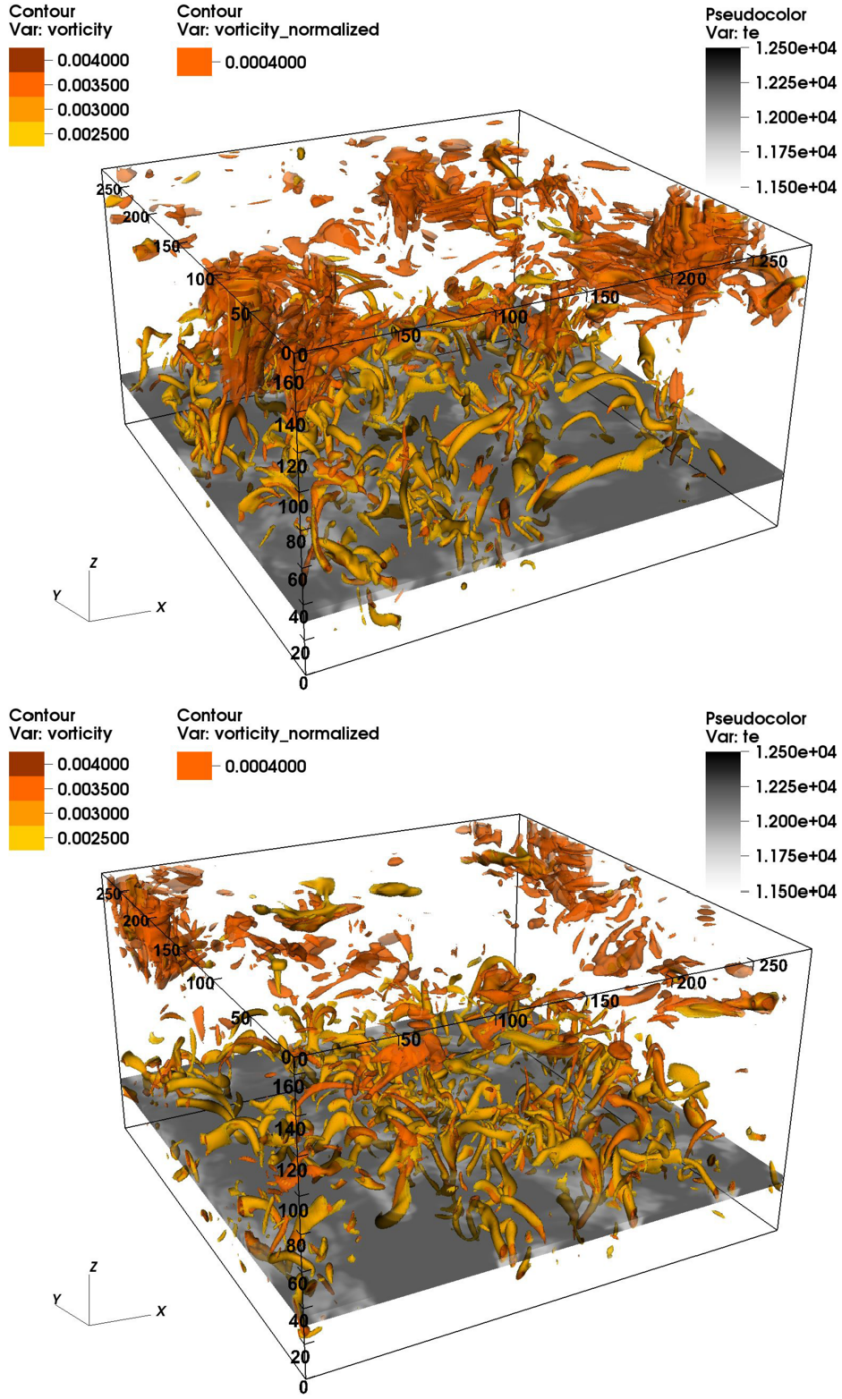
3D rendering of the $Q$-criterion in simulations of the small scale dynamo from González-Morales et al. (2020). Top panel: AMBIHALL case; bottom panel: AMBI case. The simulation has run for 1.5 solar hours after the Hall effect was introduced. Yellow contours indicate the locations of all the vortices ($Q$ above a certain value, see the legend). The orange contours indicate the locations with magnetic vortices ($Q$ normalized to plasma-$\beta $). Corrected figure from Khomenko et al. (2021)

In order to study the influence of the ambipolar and the Hall effect on vorticity, Khomenko et al. (2021) computed the proxy for vorticity from the three simulation series of González-Morales et al. (2020) using the Q-criterion, see Sect. 4.6.2. Figure 32 provides a 3D rendering of the $Q$-criterion in the AMBIHALL and AMBI simulations of González-Morales et al. (2020). It shows the location where the $Q$-criterion is above a certain threshold. One can observe that strong vorticity is always present at locations with low temperature below the photosphere in converging intergranular flows. These are also the locations of strong magnetic field concentrations. Nevertheless, it has to be kept in mind that the field strength in the small scale dynamo simulations is rather weak, limited to the equipartition between the kinetic and the magnetic energy. The plasma-$\beta $ is mostly above 1, except for the locations of a few stronger magnetic field concentrations at chromospheric heights. In order to highlight the influence of the magnetic field, Fig. 32 shows contours of $Q$ normalized by plasma-$\beta $ (orange). The comparison of the panels reveals strong vorticity associated with strong magnetic field concentrations in the AMBIHALL case. These strong vortices appear soon after the Hall effect is introduced in the simulation, and have visibly longer duration than the non-magnetic counterparts. Deeper analysis of these simulations has to be done in the future.

In conclusion, the first analysis of realistic 3D simulations including non-ideal effects reveals their potential to provide mechanisms for the generation of Alfvén waves and, possibly, vortex dissipation, but simultaneously also the potential of bringing powerful vorticity to the chromosphere. These conclusions are based on a single set of models. Significant effort is needed in the future to provide an ultimate answer on the influence of effects of a non-ideal, partially ionized solar plasma on the vorticity.

## Summary and Discussion

**Fig. 33:**
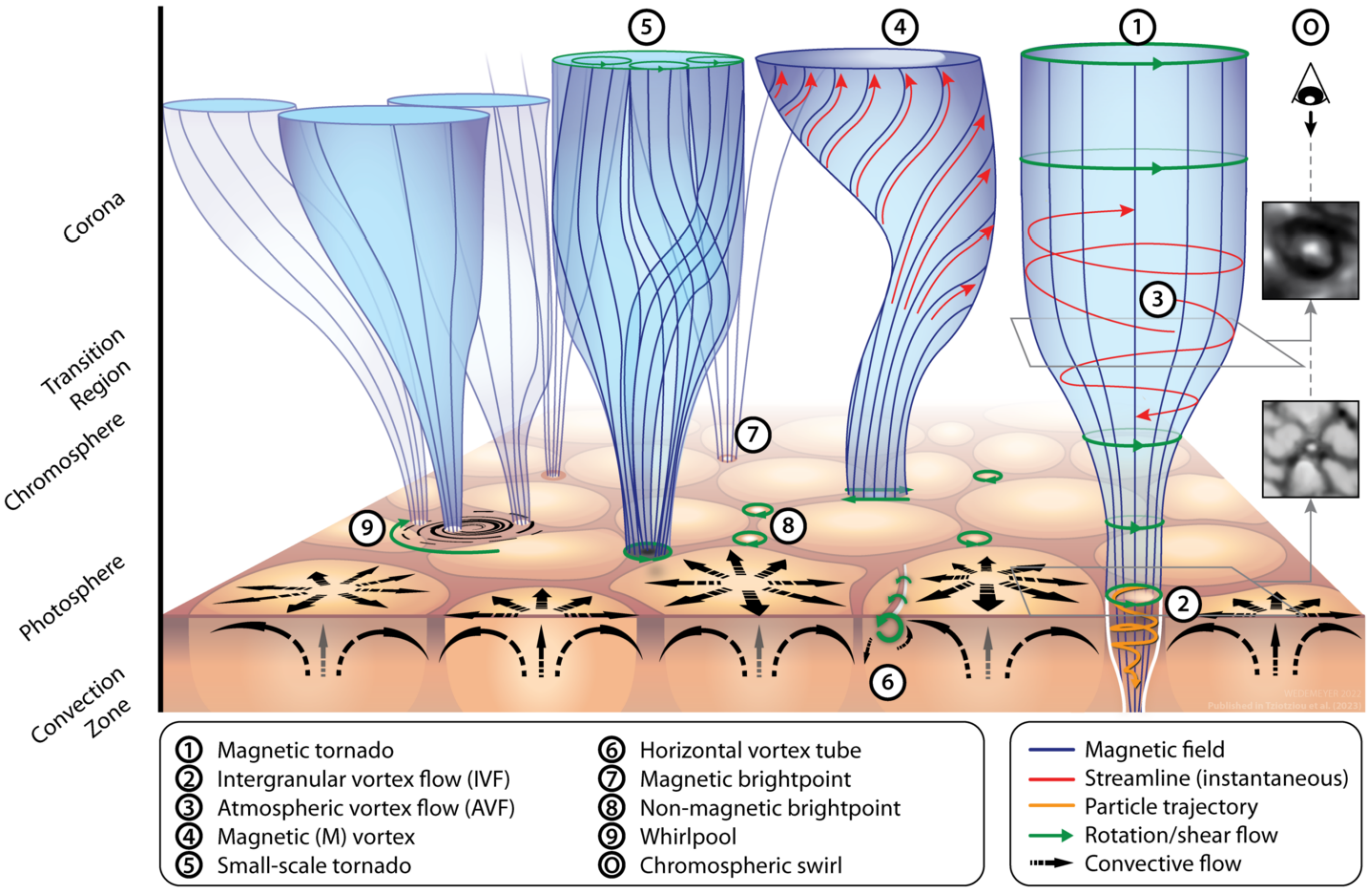
Illustration of different types of vortex flows and related phenomena in the solar atmosphere. The granulation pattern is indicated schematically in shades of brown, while the black arrows represent the corresponding (convective) flow field for the foremost granules. Magnetic field lines and instantaneous streamlines are drawn as blue and red lines, respectively. Rotation of magnetic flux structures is indicated with green arrows. Please refer to the legend for a list of the depicted phenomena. The figure has been produced by Sven Wedemeyer for the purposes of this review

In this review, we have explored the physical properties of vortex flows of mainly granular scales, as derived from observations, numerical simulations, and theoretical studies. They occur in abundance in the solar atmosphere. Such, vortex flows likely play a vital role in governing the dynamics of the solar chromosphere, particularly in the quiet Sun and coronal hole environments, and in transferring mass and energy between the photosphere and the corona. A rich tapestry of vortex behaviour exists at the very limits of present-day observational capabilities, explored in detail only recently with the aid of advanced instrumentation of ground-based solar observatories. Three-dimensional numerical simulations suggest that vortex flows may be pivotal in our understanding of the heating of the solar corona and in the initiation of a multitude of MHD waves and flows. Various observational and numerical manifestations of such phenomena have been mentioned and discussed in this review (e.g. Sects. 2, 6 and 7). They result from different drivers, such as true rotation due to the conservation of angular momentum, plasma moving in a twisted structure, rotating magnetic structure, etc., and often exhibit substructure related to their complex dynamics or the presence of, usually, Alfvénic type waves. Figure 33 tries to schematically encapsulate some of our present knowledge about vortex flows of granular scales in different atmospheric heights up to the low corona. It highlights the action of convective motions in the formation of intergranular vortex flows (IVFs) in the surface layers of the convection zone and the photosphere and the consequent formation of different atmospheric vortex flows (AVFs). It also sketches the role that photospheric shear flows play in the formation of twisted magnetic tornadoes and the action of photospheric rotational flows in the formation of kinetic tornadoes and whirlpools. Furthermore, it depicts the internal magnetic structure of different vortices and the motion of the plasma.

**Fig. 34:**
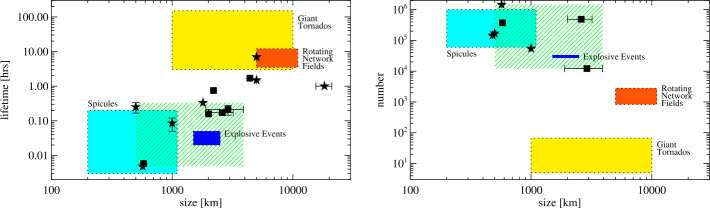
Comparison between characteristic sizes, lifetimes, and total numbers of different small-scale and rotating events on the Sun, such as spicules (cyan area; Tsiropoula et al. 2012), explosive events (blue area; Teriaca et al. 2004; Curdt et al. 2012), and rotating network fields (orange area; Zhang and Liu 2011), with vortex flows and giant tornadoes (yellow area; Wedemeyer et al. 2013b). Stars and squares (with appropriate error bars where applicable) depict, respectively, photospheric and chromospheric vortex observations (see Table 5). The green-shaded area represents observational property constrains for vortices, derived from observations where all three compared properties were reported. See Wedemeyer et al. (2013b) for an earlier version of this plot

Vortex flows of granular scales are a relatively new addition to the zoo of dynamic phenomena of the quiet-Sun atmosphere (see Fig. 9 of Wedemeyer et al. 2016, for a schematic overview of the quiet-Sun zoo, including vortex flows and torsional motions). On the solar surface and the atmosphere above, there also exist various other rotating structures (e.g. spicules) and small-scale phenomena (e.g. explosive events) whose investigation falls beyond the scope of this review. Figure 34 provides a comparison of sizes, lifetimes, and estimated total numbers of various such phenomena on the Sun, with the vortex flows discussed in this review, including giant tornadoes discussed in Sect. 6.4. The figure demonstrates that size-wise the discussed vortex flows are in the middle range of all these phenomena, number-wise they are in the higher-end of respective populations while their lifetimes are in the lower-end of respective lifetimes. We note that Wedemeyer et al. (2013b), who produced an earlier version of this figure, suggested a linear correlation between investigated sizes and numbers of individual structures, with the exception of giant tornadoes that are linked to large-scale structures (legs of prominences). However, such a linear trend seems rather inconclusive and lacks a plausible physical interpretation.

Simulations suggest that the lower solar atmosphere should be awash with twisted and swirling flows and magnetic fields, by the very fact that convective processes will naturally exhibit counter-streaming flows and by default vortices should form through the conservation of angular momentum. Yet, vortex flows have only recently started to attract significant attention and this is primarily so because of two reasons. Firstly, their observable manifestation is difficult to interpret because of complicated upwards and downwards motion in the vertical direction, as well as, complicated motion in the horizontal direction with relevant velocity measurements heavily affected by the achievable spatial resolution. Swirl interactions, make the overall picture appear even more chaotic. Secondly, swirl substructure remains unresolved even at the limits of today’s most advanced ground-based solar telescopes. With the dawn of bigger and more powerful telescopes such as DKIST (Rast et al. 2021) and the future European Solar Telescope (EST; Matthews et al. 2016; Quintero Noda et al. 2022), we can expect an exciting new window to open up into these matters. Another complicating factor is that vortex phenomena observed with different diagnostics got labelled with different names (e.g., magnetic tornado, small-scale tornado, chromospheric swirl, swirling downdraft, photospheric intensity vortex, whirlpool etc.) although they may in reality be one and the same phenomenon. Yet, some of these vortex phenomena are formed in different atmospheric layers and under different conditions, others can be explained within the same physical framework that often involves the twisting of magnetic fields, at the heart of which is either a plasma or magnetic vortex.

This review also addressed methods for the detection of solar vortices, applicable to both observational and numerical datasets. Observationally, the majority of detection methods that are designed to identify and track vortices (see Sect. 4), requires reconstruction of the flow velocities, which is often achieved with the help of an LCT technique applied to the image plane. Caution is advised when interpreting vortex signatures within FLCT velocity maps derived from intensity observations. These velocity vectors encode both spatial and temporal variations in the observed intensity, which furthermore depends on radiation transport effects. Therefore, they can potentially be different from the true plasma velocity. Tests with synthetic observations have demonstrated that FLCT approaches, used for recovering the flow velocities and consequently the detection of vortices, can be representative of the true plasma velocity under certain conditions. Hence, there can be some confidence in the detection of large number densities of vortices in the photosphere. More recently, LAVD, which adopts Lagrangian geometry to identify coherent structures and isolates vortices from their respective backgrounds, has been effectively applied to three-dimensional MHD simulations. An observational adaptation of this method could potentially yield interesting results in discerning individual swirls from the chaotic background of the chromosphere. To this end, morphological methods could also prove a powerful tool for chromospheric vortex detection. Overall, there is a need to develop more advanced methods of vortex detection, to more reliably account for the large number densities of vortices already detected in the photosphere and for the less-abundant, complex swirl structures in the chromosphere.

It is worth-noting that there is so far no conclusive observational evidence for the chromospheric vortex counter-part of photospheric intensity vortices and this certainly requires further exploration. Also missing are limb observations of small-scale vortex flows, which would allow for a side view of them and thus for a better analysis of their vertical structure. Three-dimensional simulations of expanding and twisting magnetic flux tubes, anchored in the surface layers of the convection zone and permeating through the photosphere and chromosphere, suggest the presence of three-dimensional vortex structures transcending multiple atmospheric layers up to the low corona. Therefore, observational signatures of such structures should be present co-spatially and co-temporally in both the photosphere and chromosphere.

In this review, we have also outlined the theoretical properties of vorticity in the framework of the MHD equations and its role within radiation MHD simulations, with the aim to reveal the relationship and difference between magnetic and plasma (i.e., kinetic) vorticity and the relevant dynamics of vortices in different layers of the solar atmosphere. It has been proposed in the case of the so-called magnetic tornadoes, that the rotation of the magnetic structure in the chromosphere is caused by a kinetic vortex that acts on the flux-tube basis, which roots in the convection zone/photosphere, leading to a swirling plasma response, which again is a kinetic vortex. However, the manifestation of the observed swirl is sensitive to the plasma-$\beta $. In different magnetic environments, the subtle relationship between magnetic and kinetic vorticity reverses and magnetic vorticity dictates the plasma flow, meaning that highly twisted magnetic fields embedded in the chromosphere, can direct the plasma flow into an approximately field-aligned rotational manner, thereby appearing as a swirl. Deciphering observations to find out which of these physical scenarios dominates requires the knowledge of the magnetic field in the chromosphere. The role of partial ionisation effects also becomes significant for solar vortices. More investigations are required to better understand the respective roles of ambi-polar diffusion, the battery term, and Hall effects in diffusing energy across spiralling magnetic fields.

Pertinent to the dynamical analysis of vortex flows is the generation, propagation, and dissipation of waves within them and consequently of the momentum and energy transfer to higher layers of the solar atmosphere. Despite the substantial number of relevant hydrostatic models and magnetoconvection simulations leading to considerable progress in understanding the excitation, properties, and dynamics of different wave modes within vortices, only sporadic works exist in the literature about counterpart observational signatures of waves and their dynamics. The concise observational analysis of waves is affected by the lack of simultaneous high-resolution observations in different heights that would cover a wide range of the solar atmosphere, and more importantly, by the absence of relevant magnetic field measurements that would permit its three-dimensional reconstruction. Moreover, the lack of tools for the precise chromospheric spectral line synthesis with proper multilevel-atom radiative transfer and spectropolarimetry in three-dimensional space makes the direct comparison of wave signatures between simulations and observations presently difficult to achieve.

### Similarities and Differences Between Vortex Phenomena and the Vortex Nomenclature

As already mentioned, there exist similarities between the different phenomena listed in Sect. 2. For example, given the size scales, typical lifetimes, and flow velocities, it is conceivable that solar tornadoes and giant tornadoes are one and the same phenomenon. This subject, however, to the condition that giant tornadoes are indeed a true vortical mass flow, which was found not necessarily to be the case (Schmieder et al. 2017b). Likewise, magnetic tornadoes and magnetized vortex tubes may be one and the same phenomenon. Furthermore, chromospheric swirls, which are analogous to small scale swirls are reported to be the observable chromospheric signature of magnetic tornadoes, suggesting that small-scale swirls can also be associated with magnetic tornadoes. Commonalities can also be found between the numerical studies of magnetic swirls and magnetic vortices, both having substantial twist in their magnetic field with the plasma exhibiting vortical motion when flowing along the magnetic field. This is different from magnetic tornadoes, which are thought to be straight, imperceptibly twisted, quasi statically rotating flux tubes that introduce a rotation in the plasma within them, appearing as a plasma vortex flow.

Similarities also exist between “whirlpools” (observed in the visible continuum) and “rotating magnetic network fields (EUV Cyclones)”. Despite the fact that they refer to different heights in the solar atmosphere and therefore are not the same phenomenon, they both are associated with the collective motion of photospheric bright points serving as tracers, which is different from the vortex motion associated with a single bright point. On the other hand, “small-scale tornadoes” are unique and distinguishable from small-scale swirls or chromospheric swirls (observed with the same instrument and thus with the same angular resolution) because small-scale tornadoes lack the association of observable photospheric bright points, contrary to chromospheric swirls. Also, they have relatively long lifetimes and a notable collective behaviour of swirling sub-structures in the form of individual swirls, embodied within the overall vortex flow. Also, there is no clear link between either small-scale swirls or chromospheric swirls with photospheric intensity vortices. So far, there are no observations of magnetic twist within swirls in the chromosphere and no small-scale swirl has been observed at the solar limb for a comparison with giant tornadoes at the limb. Ideally, one would like to group various manifestations of vortices into a few classes based upon simple attributes like lifetime, size-scale, and other observables. However, this turns out to be a difficult task because the fundamental physics can be different for otherwise similar phenomena and the driving forces appear to be dominated by magnetic fields in some cases, whereas the plasma flow dictates the formation and driving of vortices in others. Another distinguishing factor can be the collective interaction between multiple vortical motions versus the action of a single vortex within a magnetic flux concentration.

Concerning the nomenclature, it is obviously up to the respective paper author(s) to decide on the proper, often descriptive and appealing name of the observed or simulated vortical pattern. Given, however, the existing large naming diversity, with different names often used for similar objects, we provide below some very general nomenclature recommendations. As a general rule, we recommend the use of the term “swirl”, especially for observational studies, with the term “vortex” reserved for swirl-like appearing objects with clear evidence (e.g., from local correlation tracking or in numerical simulations) of a vortical plasma flow. The term tornado should be reserved for vortical flows that extend over large distances in height and it should be cautioned that the physics is different from tornadoes in the terrestrial atmosphere. Descriptive naming referring to the atmospheric layer in which the structure resides, e.g. “photospheric swirl/vortex” or “chromospheric swirl/vortex” is acceptable but it should be bared in mind that the described event is likely to transcend multiple atmospheric layers, not necessarily accessible through the used observational diagnostics. Furthermore, delineations of the name “swirl/vortex/tornado” into “small-scale swirl/vortex/tornado” (i.e., for cross-sectional widths less than a couple of granules) or “large-scale swirl/vortex tornado” (i.e., for cross-sectional widths greater than a few granules) can be used to describe the size of the object. Obviously, the attribute “magnetic” should only be used in the unambiguous presence of magnetic fields together with the swirl or vortex. Therefore, an observed phenomenon should only be labelled as “small-scale/large-scale magnetic swirl/vortex/tornado” when there is compelling evidence that the magnetic field governs the dynamics, otherwise, when the plasma dictates the dynamics, this attribute should be omitted.

### Beyond the Sun

As a final remark, we note that the generation of vortex flows due to the conservation of angular momentum is an integral part of all hydrodynamic flows in both non-magnetic or weakly magnetised stratified media. Therefore, magnetic and kinetic vortices are expected to occur in the surface convection layers of stars other than the Sun. Examples of vortex flows in stellar atmospheres have been found in 3D numerical simulations of M-dwarf models with $T_{\mathrm{eff}} = 3240~\text{K}$, $\log g = 4.5$, and an initial vertical magnetic field strength of $|B_{0}| = 100~\text{G}$ (Wedemeyer et al. 2013a) and of a red giant star (Wedemeyer et al. 2017) with $T_{\mathrm{eff}} = 4010~\text{K}$, $\log g = 1.5$, but no magnetic field ($|B_{0}| = 0~\text{G}$). The magnetic M-dwarf simulation formed magnetic tornadoes, whereas the purely hydrodynamic red giant simulation revealed characteristically similar photospheric vortex flows. Swirling downdrafts that form non-magnetic bright points (see Table 2) have also been detected in magnetic field-free 3D radiation hydrodynamic simulations of stellar atmospheres from spectral types K8V to F5V, most conspicuously in the model of K2V (Salhab et al. 2018). These examples underline that the formation of vortex flows is a natural consequence of stellar surface convection.

We note that vortices also occur in the gas giant planetary and exoplanetary systems, e.g., at the poles of Jupiter as was discovered by NASA’s Juno mission. However, in these cases, it is probably the Coriolis force, hence their rotation, that plays a central role rather than simply angular momentum conservation of a converging, perturbed flow as in the case of stars.

## Outlook

Many more swirl observations need to be conducted to validate or falsify predictions from simulations, in particular concerning the connectivity of vortex tubes across different atmospheric layers. The limb counterpart of small-scale swirls has not yet been observed. To date, all observed small-scale swirls have been detected relatively close to the solar disk centre. There, the swirl cross-section is best visible and the prevailing spiral features provide best evidence for the existence of a vortex. However, as it is demonstrated in Sect. 8.2.3 with results from computational modelling, small-scale vortices have observable signatures near or at the limb. Their observational identification and confirmation amongst the forest of spicules is a challenge yet to be tackled. Limb observations can potentially provide profound insights into the multi-layered character of vortices.

One of the most important characteristic of vortex flows is the twist or rotation of the magnetic field, particularly in the chromosphere. However, so far, no magnetic field signatures have been detected in the chromosphere of small-scale swirls. Are the magnetic fields of chromospheric swirls indeed rotating, as simulations suggest and takes this rotation place in conjunction with the apparent plasma ring fragments or not? Addressing this question will shed light on the relative significance of magnetic vorticity vs plasma vorticity in the driving and the formation of swirls. Measurements of the magnetic fields in different heights of the atmosphere and, if possible, reconstruction of its three-dimensional structure within a vortex flow, would allow for a proper investigation of the excitation, propagation, and dissipation of the various wave modes.

Ideally, we would like to observe the three-dimensional structure of vortex tubes and the corresponding magnetic field. This is, however, very ambitious for mainly two reasons. First, one would need to combine observations from largely different aspect angles for a stereoscopic reconstruction. In principle, this is possible from space-based observatories at different locations such as IRIS and PHI or SPICE on Solar Orbiter, or from Solar Orbiter in combination with a ground based solar observatory. However, second, very high spatial resolution is needed to observe small-scale swirls, which is typically not available from solar space observatories. Best chances are probably offered by combining observations with Solar Orbiter with high-resolution ground-based facilities such as DKIST, GREGOR, or SST but also with the upcoming SUNRISE III mission. Looking further ahead, the Multi-slit Solar Explorer (MUSE) and the Solar-C EUVST will be available.

Still missing is the co-spatially and co-temporally matching of chromospheric swirls with their photospheric counterpart. Since the formation of IVFs (due to solar convection) is thought to be a prerequisite for the formation of AVFs/magnetic tornadoes, while the latter require further conditions to be fulfilled for their formation, it seems to be logical that AVFs should occur less frequent than IVFs (see Sect. 5). Simulations indicate that the formation of AVFs involve the existence of a co-local magnetic field, yet to be observationally resolved for a better understanding of how and when a photospheric swirl acquires a chromospheric counterpart. Knowledge of the magnetic environment is significant in view of explaining why small-scale swirls predominantly appear in the quiet Sun and in coronal hole environments, and seemingly not in active regions. On the other hand, vortical motions can also be genuinely generated in the chromosphere or corona without connection to the photosphere and it is not clear whether magnetic field is involved in their generation or not. Also, the generation of AVFs via IVFs must be critically reviewed; other mechanisms, predominantly magnetic in nature, cannot be excluded yet.

Pertinent to the formation of IVFs is a missing, systematic study on vorticity generation, on how vorticity transport occurs in the solar upper convection zone and the photosphere, and consequently on the relative budget between locally-generated and transported vorticity from deeper layers into the photosphere, as already mentioned at the end of Sect. 5.

It is still unknown whether or not small-scale swirls exist at the foot-point of a giant tornado. Given that giant tornadoes (i.e. large-scale swirls by our definition) are mostly observed at the limb, this is a good place to start searching for limb signatures of small-scale swirls. Exploring the relationship between small-scale swirls and giant tornadoes should lead to a better understanding of the multi-scale nature of vortex flows. Furthermore, up today, only a few observational studies exist on horizontal vortex tubes that have been inferred from combined observations and simulations to exist at the edges of granules. Their further polarimetric observation promises to shed light on the shallow re-circulation of magnetic fields and the working of the near surface small-scale dynamo.

Regarding numerical simulations, advances in 3D radiation MHD simulations, incorporating the physics of the chromosphere, transition region, and corona and the physics of partially ionised plasma, are required to track the energy transport from the subsurface layers to the outer atmosphere. We note that the dependence of the small-scale vortex formation and its dynamics on the atmospheric magnetic environment (field strength and topology) has not been extensively studied with simulations yet. A systematic study of the effects of numerical resolution, subgrid diffusivity, and turbulence models on small-scale vortex structure, formation, and evolution is also lacking and would be worthwhile an effort to be taken. Likewise, a systematic study on the connectivity between photospheric and chromospheric swirls and their height dependent behavior is still to be performed, although this was done for single exemplary cases. It needs to be further explored how important deviations from ideal MHD are for vortex flows. New methods are to be developed for the analysis of numerical simulations of vortices to shed light on the physics of the driving and the formation of swirls, and their potential impact in the atmosphere above. For instance, what type of MHD waves can be excited by vortex motions occurring on different spatial scales? The tension inherited through the twisting of magnetic fields under the action of a vortex is a perfect mechanism for the generation of torsional Alfvén waves, as well as, many other oscillatory modes characteristic of magnetic flux tubes. What role do each of these wave modes play at different spatial scales in different atmospheric layers? What is their net contribution to the total energy budget and what is their heating signatures? The energy transfer processes in a partially ionised plasma have not yet been explored in sufficient detail. The respective roles of vortex flows and MHD waves in the energy budget requires further study as well as the three-dimensional wave mode structure generated by vortex tubes. To date, there is no comprehensive numerical model, which incorporates the energy transfer in vortices ranging from their initiation in the convection zone/photosphere to the dissipation in the chromosphere/corona. A first attempt in this direction is the recent simulation by Breu et al. (2022). However, there also exists the possibility that vortices do not originate in the photosphere or the subsurface layers but that they may be generated in the outer atmosphere. What are the formation mechanisms in this case? The formation of vortices may be quite different when comparing small-scale swirls with giant tornadoes, which are typically associated with prominences and filamentary channels.

Increasing interest attracted lately the idea of erupting vortex tubes as a possible solar origin of magnetic switchbacks that are observed by the Parker Solar Probe. In this review, we restricted ourselves to small-scale vortical flows of spatial scales of less than a couple of granules. We think that the switchbacks observed by the Parker Solar Probe originate from much larger scale phenomena. Also, coronal loops harbour vortical flows as recent simulations by Breu et al. (2022) suggest. One could speculate that such coronal loop swirls could be connected to the switch-back phenomenon. Whether these coronal loop swirls are connected to photospheric swirls or not is still an open question.

In many ways the study of vortex flows in the lower solar atmosphere is only now coming to fruition, heralded by a new generation of ground-based instrumentation and high performance computing capability. To gain a deeper insight into the interplay between magnetic twist and plasma vorticity of vortex flows in the solar atmosphere, we ultimately require observations of much higher spatial and temporal resolution and of much superior spectropolarimetric sensitivity than is available today. Simultaneous polarimetric observations in different layers of the atmosphere are essential too. Fortunately, a new generation of observations are on the horizon from both advanced ground-based (e.g., DKIST) and space-based (e.g., Solar Orbiter) observatories and more is anticipated within the coming decade from forthcoming facilities, such as EST. The next generation of high-resolution imaging spectropolarimeters (such as VTF at DKIST) and integral field units (such as image slicers, lenslet arrays, or fiber optical devices) can be expected to greatly enhance our understanding of the spatial/temporal variability of three-dimensional motions in vortices and of their three-dimensional magnetic properties, at high temporal cadence and simultaneously in the photosphere and chromosphere. As demonstrated in this review, much has been explored but there is still more to be answered, to the benefit of not only the field of solar and stellar physics but also of other scientific domains that deal with the nature and physics of vortices.

## Footnotes

1 With solar surface we mean here and in the following the surface of optical depth unity, which also marks the transition from mainly convective to mainly radiative energy transport.

2 Straight line segments joining any two points in the region lie entirely within the region.

3 According to Haller (2001) “a material surface is a smooth, evolving surface of particles”.
